# Sulfoxonium Ylides in Aminocatalysis: An Enantioselective Entry to Cyclopropane-Fused Chromanol Structures

**DOI:** 10.1021/acs.orglett.2c02204

**Published:** 2022-07-20

**Authors:** Giorgiana Denisa Bisag, Pietro Pecchini, Michele Mancinelli, Mariafrancesca Fochi, Luca Bernardi

**Affiliations:** † Department of Industrial Chemistry “Toso Montanari”, Center for Chemical Catalysis - C^3^, and INSTM RU Bologna, Alma Mater Studiorum - University of Bologna, V. Risorgimento 4, 40136 Bologna, Italy

## Abstract

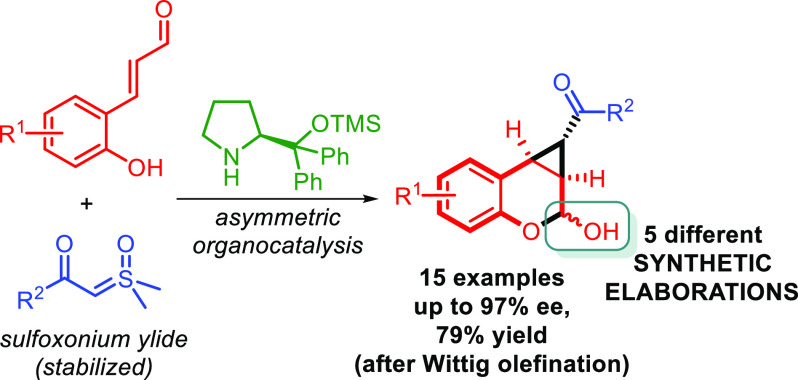

The 1,1a,2,7b-tetrahydrocyclopropa[*c*]chromene, arising from fusion of chromane and cyclopropane rings is the core of medicinally relevant compounds. Engaging sulfoxonium ylides in enantioselective aminocatalytic reactions for the first time, a convenient entry to this scaffold is presented. Several ring-fused derivatives were obtained in moderate-to-good yields and enantioselectivities and with perfect diastereoselectivity at the cyclopropane, using an α,α-diphenylprolinol aminocatalyst. The versatility of the hemiacetal moiety in the products was leveraged to effect various synthetic manipulations.

The cyclopropane ring is present in numerous pharmacologically active compounds. The fame of this ring in medicinal chemistry is not only due to the strain of the cycle, which reserves a reactivity somewhat similar to an olefin, but also to the presence of C–H bonds shorter and stronger than those of common alkanes. Furthermore, the coplanarity of the three carbon atoms makes the reactivity displayed by cyclopropane truly unique.^1^ In this context, the specific tricyclic 1,1a,2,7b-tetrahydrocyclopropa[*c*]chromene framework, arising from fusion of chromane and cyclopropane rings, is the core of several medicinally relevant compounds (Scheme 1a). Examples include 8-carboxy-7-sulfonamido derivatives **I**, whose activity against methionyl aminopeptidase 2 suggests their use in the treatment of liver disorders and obesity,^2^ urea **II** (MIV-160), a reverse transcriptase inhibitor studied for anti-HIV therapy,^3^ and carboxylic acid **III**, a member of a series of fused cyclopropane derivatives agonists of G-protein coupled receptor 40 (GP40) and potentially useful in the treatment of type 2 diabetes.^4^ Furthermore, “cyclopropanochroman” natural products, such as radulanins I–K (**IV–VI**), have been isolated from liverwort extracts in racemic or enantiopure form.^5^ Radulanin K from *Radula javanica* has shown to inhibit the release of superoxide anion radical from guinea pig macrophage.^6^

**Scheme 1:**
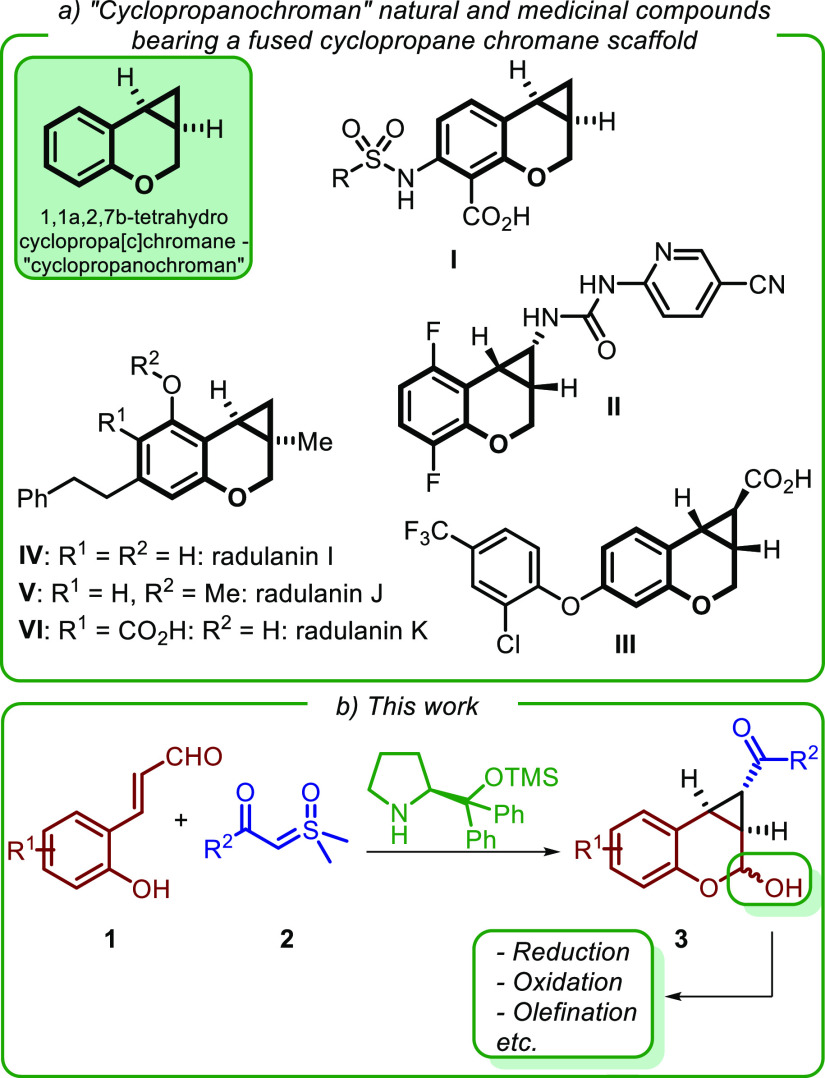
(a) Natural and Medicinally Relevant Compounds Embedding the 1,1a,2,7b-TH-Cyclopropa[*c*]chromene Framework; (b) This Work: enantioselective Access to This Scaffold via Aminocatalytic Cyclopropanation of Enals **1** with Sulfoxonium Ylides **2**

In the frame of our interest in asymmetric aminocatalysis^7^ and sulfoxonium ylide chemistry,^8^ we herein report an enantioselective access to cyclopropane-fused chromanol derivatives **3** via aminocatalytic Corey–Chaykovsky-type cyclopropanation^9^ of 2′-hydroxycinnamaldehydes **1** with stabilized sulfoxonium ylides **2** (Scheme 1b). Aminocatalytic cyclopropanation reactions of other α,β-unsaturated aldehydes have been reported. In this context, examples of Corey–Chaykovsky-type reactions are relatively rare and restricted to α-keto sulfonium ylides,^9c−9f^ while cyclopropanations with α-halo(di)carbonyl compounds, 1-bromonitroalkanes, and activated benzyl halides (e.g., 2,4-dinitrobenzyl chloride) are more abundant.^10^ The latter group of reactions is generally performed with Jørgensen–Hayashi type catalysts,^11^ whose simplest congener proved to be effective in our case too (Scheme 1b). This reaction represents the first example of utilization of sulfoxonium ylides in asymmetric aminocatalysis^12^ and affords the tricyclic ring-fused derivatives **3** with very good stereocontrol. Importantly, the connectivity and relative stereochemistry of these compounds match the core of GP40 agonist **III** (Scheme 1a). Lastly, besides providing an alternative, and enantioselective, approach to this scaffold,^13,14^ this methodology affords adducts (**3**) carrying a hemiacetal functionality, which can be leveraged as a synthetic handle enabling access to a variety of compounds.

During our initial studies on the reaction between 2′-hydroxycinnamaldehyde **1a** and sulfoxonium ylide **2a** under the promotion of a common Jørgensen–Hayashi catalyst^11^ (Table 1), we noticed an immediate color change by mixing aldehyde **1a** with the secondary amine catalyst in CDCl_3_. Such a color change can be attributed to the formation of a stable and nucleophilic hemiaminal adduct.^15^ In order to revert this hemiaminal to an electrophilic iminium ion species, presumably *E*-configured,^16^ 20 mol% of benzoic acid co-catalyst was added followed by the nucleophilic sulfoxonium ylide **2a**. To our delight, we observed the formation of the desired chromanol derivative **3aa**,^17^ which was derivatized by Wittig olefination into the corresponding **4aa**, obtained as a highly prevalent *E*-isomer for isolation and determination of the enantiomeric excess. Immediately, we understood that the reaction was characterized by promising results in terms of yield and enantioselectivity. Indeed, when the reaction was performed under these standard conditions, 50% yield and 88% enantioselectivity were achieved (entry 1). Furthermore, regarding the chirality centers of the cyclopropane ring, the diastereoselectivity of the reaction appeared to be complete. Because of the short reaction time, we decided to decrease the concentration of the reaction medium, which resulted in a cleaner reaction profile and increased values of yield and enantiomeric excess of product **4aa** (entry 2). Next, we continued the optimization reaction using different co-catalysts and, among all the results (see also the SI), the reaction with acetic acid gave product **4aa** with slightly better enantioselectivity, albeit longer reaction time (entry 3). At this stage, we decided to explore the buffer system AcONa/AcOH. When the reaction was performed with equal amounts of acetic acid and sodium acetate, an increment of the yield was achieved, while the enantioselectivity decreased (entry 4). Then, when the reaction was performed with different relative amounts of the acid and its conjugate base, two different behaviors were observed. With an excess of sodium acetate, the yield of product **4aa** decreased again while its enantiomeric excess increased slightly (entry 5). Running the reaction with more acetic acid than sodium acetate improved the yield, but the enantioselectivity dropped (entry 6). Surprisingly, we found that when the reaction was performed with sodium acetate as the only co-catalyst both the yield and enantioselectivity of product **4aa** increased (entry 7). Our current understanding is that the acidity of 2′-hydroxycinnamaldehyde **1a** is enough to form sufficient amounts of the reactive iminium ion for the reaction to proceed. Meanwhile, sodium acetate might be helpful for scavenging more acidic species which could be harmful to the acid-sensitive sulfoxonium ylide. Indeed, a reaction performed without additives afforded product **4aa** in comparably high enantiomeric excess but lower yield (entry 8). Having chosen sodium acetate as the best additive, we ascertained that the results in chloroform (entry 9) are in line with results obtained so far in the corresponding deuterated solvent. Interestingly, a reaction performed using a sulfonium, instead of sulfoxonium, ylide did not afford product **3aa** under these reaction conditions. Furthermore, cinnamaldehyde was found to be unreactive toward sulfoxonium ylide **2a**, even when the Jørgensen–Hayashi catalyst was combined with acid co-catalysts. Thus, 2′-hydroxycinnamaldehydes showcase a distinct reactivity compared to their simpler nonhydroxylated counterparts,^16^ at least for this reaction.

**Table 1:**
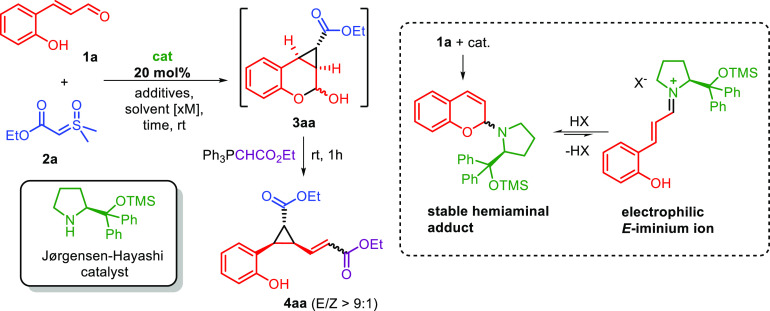
Representative Optimization Results^a^

entry	solvent (M)	time (h)	co-catalysts (mol%)	yield of **4aa**^b^ (%)	ee of **4aa**^c^ (%)
1	CDCl_3_ (0.5)	1	PhCOOH (20)	50	88
2	CDCl_3_ (0.1)	2	PhCOOH (20)	57	95
3	CDCl_3_ (0.1)	12	AcOH (20)	52	96
4	CDCl_3_ (0.1)	12	AcONa (20) + AcOH (20)	74	79
5	CDCl_3_ (0.1)	12	AcONa (20) + AcOH (10)	65	82
6	CDCl_3_ (0.1)	12	AcONa (10) + AcOH (20)	75	74
7	CDCl_3_ (0.1)	12	AcONa (20)	67	97
8	CDCl_3_ (0.1)	12		41	96
9	CHCl_3_ (0.1)	12	AcONa (20)	65	97

a Reaction conditions: **1a** (0.1 mmol), **2a** (0.15 mmol), catalyst (0.02 mmol), additive, solvent, rt. Then phosphorus ylide, rt, 1 h.

b Isolated yield after column chromatography.

c Determined by CSP (chiral stationary phase) HPLC analysis after column chromatography.

We then moved to evaluate the generality of the reaction after having verified that the reaction can be carried out with similar results on a 1 mmol scale (Scheme 2). The variation of the sulfoxonium ylide **2** reported in Scheme 2 showed that both short-chain and long-chain ester substituents are very well tolerated, giving products **4ab** and **4ac** with comparable results in terms of yield and very good enantioselectivity. In addition, bulky substituents such as the isobutyl or the *tert*-butyl group on the ester moiety give products **4ad** and **4ae**, respectively, in good yields and with high enantiomeric excesses. Similarly, the use of an allylic or a benzylic ester did not significantly affect either the yield or the enantioenrichment of products **4af** and **4ag**. Next, the sulfoxonium ylide **2h** with a ketone instead of an ester substituent was tested. Product **4ah** was obtained in a lower yield, possibly due to the less nucleophilic nature of this ylide, but with high enantiomeric excess. Finally, using a different phosphorus ylide, compound **4′ab** with two methyl esters was prepared, and its relative and absolute configurations were determined as 1*R*,2*R*,3*S* by means of NOESY-1D NMR and the electronic circular dichroism (ECD) method (see the SI). This assignment, fully in line with the proposed pathway,^17^ was extended by analogy to all products **4**.

**Scheme 2:**
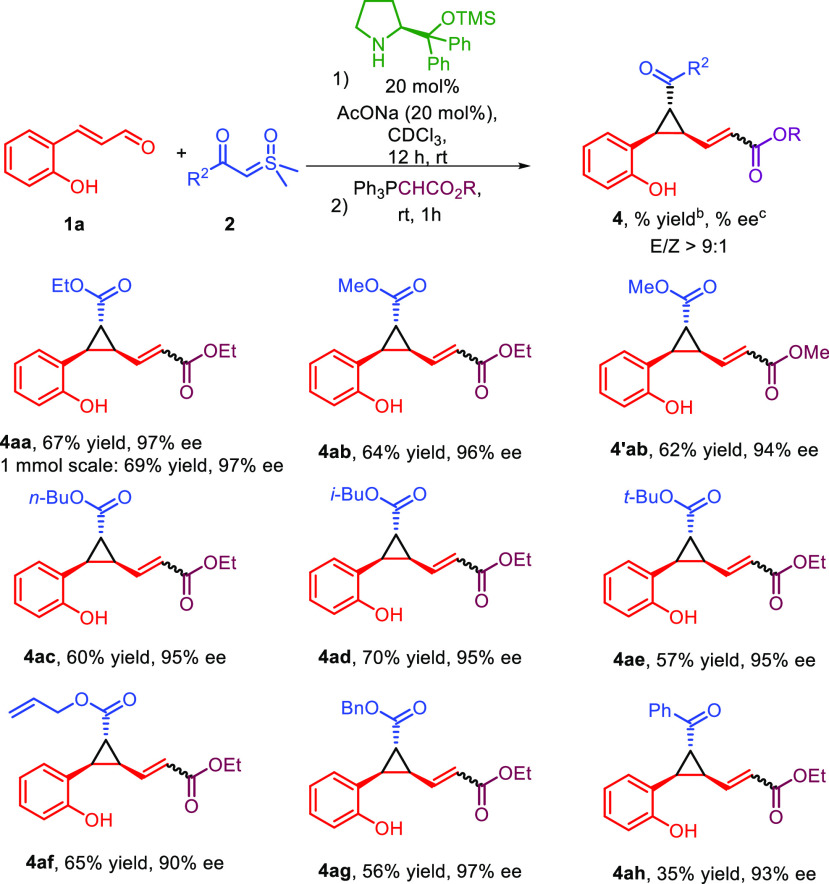
Sulfoxonium Ylide **2** Substrate Scope Conditions: **1a** (0.1 mmol), **2** (0.15 mmol), catalyst (0.02 mmol), AcONa (0.02 mmol), CDCl_3_ (1 mL), rt, 12 h. Isolated yield after column chromatography. Determined by CSP (chiral stationary phase) HPLC analysis after column chromatography.

We then explored the reactivity of sulfoxonium ylide **2a** with different 2′-hydroxycinnamaldehydes **1b**–**g**, and the results are reported in Scheme 3. A 4′-methyl substituent gave product **4ba** in good yield and high enantiomeric excess, while the same group at the 5′ position led to product **4ca** in a lower yield but still high enantioselectivity. A more electron-donating substituent like a methoxy group at different positions was also tolerated, delivering products **4da**, **4fa**, and **4ga** in moderate to good yields and good enantiomeric excesses. Interestingly, product **4fa** bears an oxygenated substituent at the same position of the aryloxy group of GP40 agonist **III** (Scheme 1). Finally, using an electron-withdrawing substituent like a chlorine atom led to the corresponding product **4ea** with good results.

**Scheme 3:**
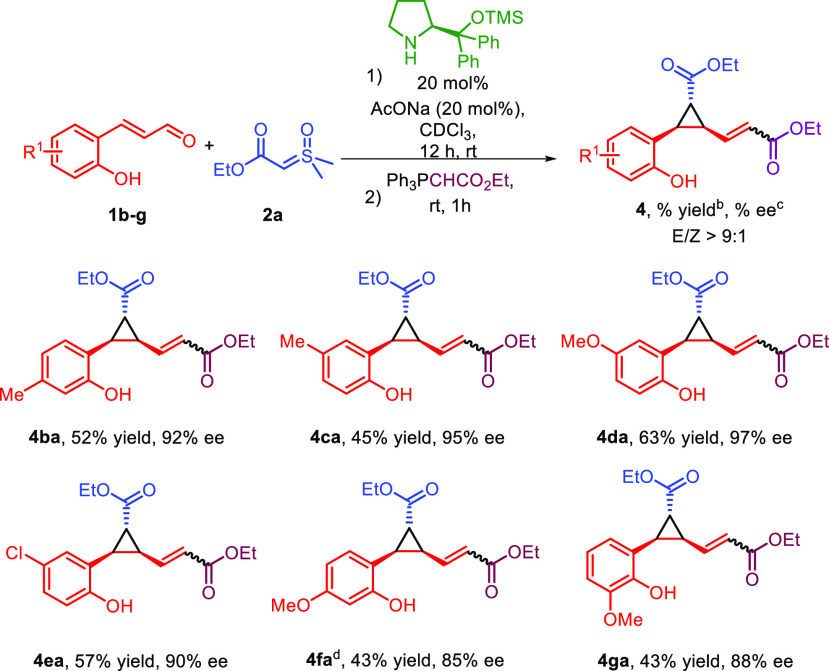
2′-Hydroxycinnamaldehyde **1** Substrate Scope Conditions: **1b**–**g** (0.1 mmol), **2a** (0.15 mmol), catalyst (0.02 mmol), AcONa (0.02 mmol), CDCl_3_ (1 mL), rt, 12 h. Isolated yield after column chromatography. Determined by CSP (chiral stationary phase) HPLC analysis after column chromatography. 1 equiv of NaOAc was used.

As mentioned in the introduction, the backbone of the catalytic products is present in numerous natural and medicinal compounds. For this reason, we moved to explore their synthetic versatility (Scheme 4). When **3aa** was treated with PCC, the hemiacetal group could be oxidized to deliver coumarin **5aa** in moderate yield. The readily obtained methyl acetal of **3aa** could be smoothly reduced to the corresponding chromane **6aa** using triethylsilane in the presence of BF_3_·OEt. Using sodium borohydride, the fleeting aldehydic function could instead be converted into a primary alcohol, obtaining product **7aa** in very good yield. Protocols combining the catalytic reaction and these reductions or oxidations in one-pot^18^ fashion were also implemented (see the Supporting Information). Using these streamlined and convenient methods, product **5aa** was obtained with comparable yield, while **6aa** and **7aa** were afforded with lower yield values. Product **4aa** resulting from Wittig olefination of **3aa** was subjected to an intramolecular diastereodivergent oxa-Michael reaction.^19^ When the reaction was performed with bifunctional catalysts derived from pseudoenantiomeric *Cinchona* alkaloids, it was possible to direct the diastereoselectivity of the reaction either toward the *cis*-**8aa** or the *trans*-**8aa** derivative. The intrinsic diastereomeric relationship between the transitions states leading to the *cis*-**8aa** and to the *trans*-**8aa** isomer justifies the requirement of different (i.e., not enantiomeric) catalytic structures for the two reactions (see the Supporting Information).^20^

**Scheme 4:**
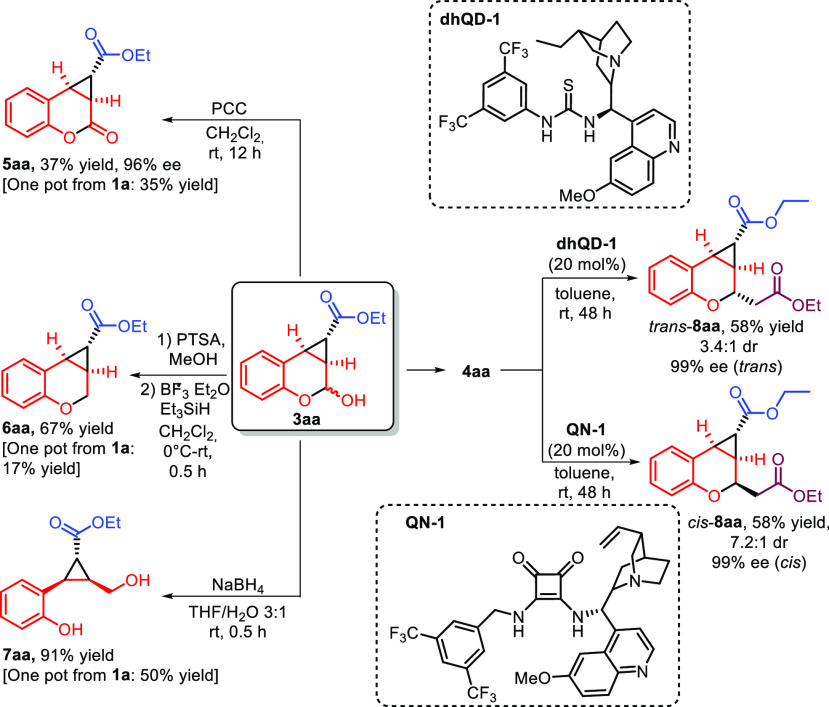
Synthetic Elaborations

In conclusion, we have developed a catalytic enantioselective reaction between 2′-hydroxycinnamaldehydes **1** and stabilized sulfoxonium ylides **2**, affording cyclopropane-fused chromane derivatives **3** in moderate yields and excellent enantioselectivities. Besides the evident relevance of the scaffold of these products in medicinal compounds, the presence of a versatile hemiacetal moiety allowed us to perform various synthetic elaborations. Disclosing the first utilization of sulfoxonium ylides under aminocatalytic conditions, these results add an important piece to the still poorly disclosed puzzle of asymmetric organocatalysis with sulfoxonium ylide substrates.^8b,12^

## Supporting Information Available

Additional optimization studies, catalytic cycle, determination of the relative and absolute configuration of products **4′ab** and **8aa**, experimental section, copies of NMR and IR spectra and HPLC traces (PDF)
