# Supplementary material for: Sulfoxonium Ylides in Aminocatalysis: An Enantioselective Entry to Cyclopropane-Fused Chromanol Structures
Source: Org Lett. 2022 Jul 20;24(29):5468–73. doi: 10.1021/acs.orglett.2c02204 (PMC9344464; doi:10.1021/acs.orglett.2c02204)

# Supporting Information

# Sulfoxonium Ylides in Aminocatalysis: An Enantioselective Entry to Cyclopropane-Fused Chromanol Structures

*Giorgiana Denisa Bisag, Pietro Pecchini, Michele Mancinelli, Mariafrancesca Fochi,* and Luca Bernardi**

Department of Industrial Chemistry "Toso Montanari", Center for Chemical Catalysis - $C^3$, and INSTM RU Bologna, Alma Mater Studiorum - University of Bologna, V. Risorgimento 4, 40136 Bologna (Italy).

E-mail: mariafrancesca.fochi@unibo.it; luca.bernardi2@unibo.it

## Table of contents

Optimization of reaction conditions for product **4aa**: additional results--------------------------------S1

Proposed reaction pathway for the catalytic reaction-----------------------------------------------------S4

Additional experiments for the diastereodivergent oxa-Michael reaction **4aa** → **8aa**----------------S5

General methods and materials---------------------------------------------------------------------------S7

Starting Materials-------------------------------------------------------------------------------------------S8

Synthesis of products **4**: general procedure and characterization-----------------------------------------S9

Synthetic elaborations---------------------------------------------------------------------------------------S17

Determination of the relative configuration of compounds **4'ab** and **3ab**------------------------------S22

Determination of the relative configuration of compounds *cis*-**8aa** and *trans*-**8aa**--------------------S26

Determination of the absolute configuration of compounds **4'ab** and **3ab**-----------------------------S28

Copies of $^1$H and $^{13}$C NMR spectra of products **4-8**--------------------------------------------------S34

Copies of HPLC traces of products **4-8**-----------------------------------------------------------------S54

Copies of IR spectra of products **4-8**---------------------------------------------------------------------S72

## Optimization of reaction conditions for product 4aa: additional results

Different catalysts were tested in the reaction between 2'-hydroxy cinnamaldehyde **1a** and stabilized sulfoxonium ylide **2a** and the results are reported in Table S1. Only the reaction performed with catalyst **A** gives product **4aa** with promising results in terms of yield and enantioselectivity (entry 1). Indeed, a little change on the catalyst's backbone such as a methyl as O-protecting group or 3,5-$CF_3$ as substituents on the two aromatic rings leads to obtaining product **4aa** with a lower value of yield and enantioselection (entries 2 and 3). While, performing the reaction with imidazolidinone **D** as catalyst, product **4aa** was present in the reaction mixture only in traces and in a racemic form.

*Table S1. Catalyst screening*[a]

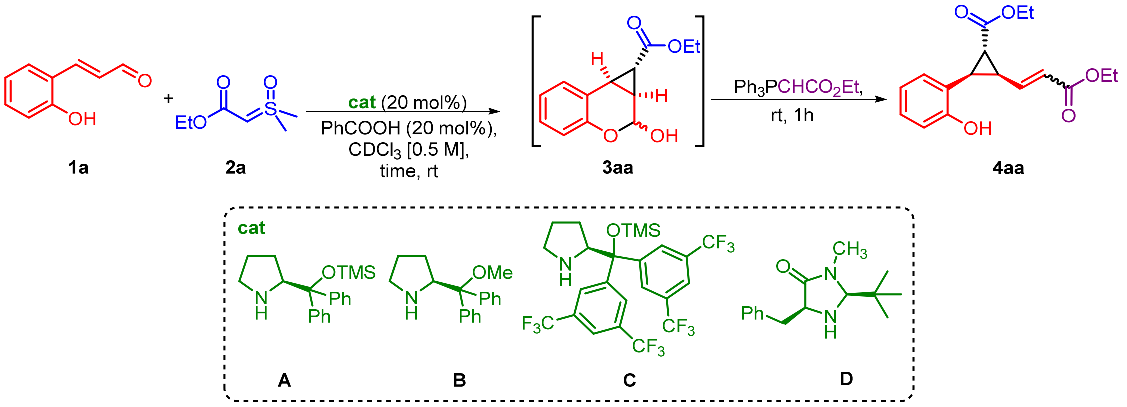


| entry | cat | ee [%][b] | Yield [%][c] | Time [h] |
|---|---|---|---|---|
| **1** | **A** | 88 | 50 | 1 |
| **2** | **B** | 7 | 27 | 12 |
| **3** | **C** | 52 | 22 | 12 |
| **4** | **D** | *rac* | 9 | 12 |

[a] Reaction conditions: **1a** (0.1 mmol, 1 equiv.), **2a** (0.15 mmol, 1.5 equiv.), PhCOOH (0.02 mmol, 20 mol%) catalyst **A-D** (0.02 mmol, 20 mol%) and $CDCl_3$ (200 µL), rt, 1-12 h. [b] Enantiomeric excess determined by CSP-HPLC. [c] Yield determined after chromatographic column on silica gel.

Once the right catalyst for the reaction was identified, a solvent screening was performed and the results are reported in Table S2. Numerous solvents were tested, but product **4aa** was obtained only by performing the reaction in toluene or in halogenated solvents (entries 1-3). Indeed, performing the reaction in THF, MTBE or EtOAc only starting materials were present in the reaction mixture without traces of product **4aa** (entries 4-6). Performing the reaction in toluene (entry 2) product **4aa** was obtained with a lower value of yield and enantiomeric excess, compared to $CDCl_3$. While using dichloromethane, comparable results in terms of yield and enantio-selection were obtained (entry 3). Given the convenience of using a deuterated solvent during the optimization process, and the good results obtained, we decided to use deuterated chloroform as solvent for the next screening.

*Table S2. Solvent screening*[a]

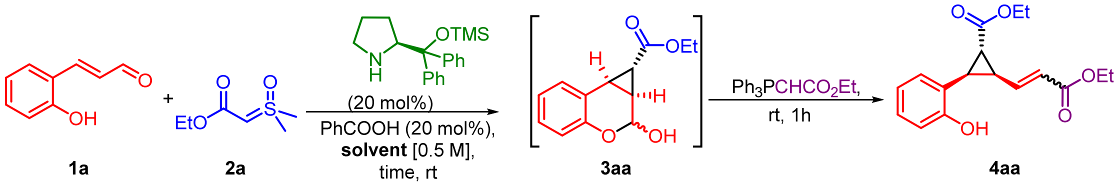


| entry | solvent | ee [%][b] | Yield [%][c] | Time [h] |
|---|---|---|---|---|
| **1** | **$CDCl_3$** | 88 | 50 | 1 |
| **2** | **PhMe** | 63 | 37 | 12 |
| **3** | **DCM** | 85 | 50 | 1.5. |
| **4** | **THF** | / | / | 12 |
| **5** | **MTBE** | / | / | 12 |
| **6** | **EtOAc** | / | / | 12 |

[a] Reaction conditions: **1a** (0.1 mmol, 1 equiv.), **2a** (0.15 mmol, 1.5 equiv.), PhCOOH (0.02 mmol, 20 mol%) catalyst **A** (0.02 mmol, 20 mol%) and solvent (200 µL), rt, 1-12 h. [b] Enantiomeric excess determined by CSP-HPLC. [c] Yield determined after chromatographic column on silica gel.

To improve both the enantiomeric excess and the yield of the product **4aa** we investigated the dilution of the reaction (Table S3). We found that performing a more diluted reaction (0.1 M, entry 2 instead of 0.5 M entry 1), product **4aa** can be obtained with higher values of both yield and enantio-selection.

*Table S3. Concentration*[a]

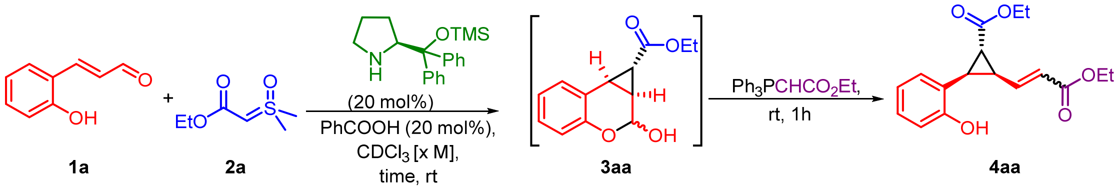


| entry | Concentration [M] | ee [%][b] | Yield [%][c] | Time [h] |
|---|---|---|---|---|
| **1** | **0.5** | 88 | 50 | 1 |
| **2** | **0.1** | 95 | 57 | 2 |

[a] Reaction conditions: **1a** (0.1 mmol, 1 equiv.), **2a** (0.15 mmol, 1.5 equiv.), PhCOOH (0.02 mmol, 20 mol%) catalyst **A** (0.02 mmol, 20 mol%) and $CDCl_3$ (200 or 1000 µL), rt, 1-2 h. [b] Enantiomeric excess determined by CSP-HPLC. [c] Yield determined after chromatographic column on silica gel.

In the end, we moved to evaluate the influence of the additives as co-catalysts in the reaction between aldehyde **1a** and sulfoxonium ylide **2a**. We found that performing the reaction with CSA as additive, product **4aa** can be obtained with a very high value of enantiomeric excess but a lower yield (entry 2). Moving on to evaluate the different acidity of benzoic acid derivatives, we understood that performing the reaction with a rather acidic benzoic acid, *p*-$NO_2$-benzoic acid, it is possible to improve the yield of product **4aa**, compared to the simple benzoic acid, while the enantiomeric excess experiences a considerable decrease (entry 3). Performing the reaction with a less acidic benzoic acid, *p*-MeO-benzoic acid, both the value of yield and enantioselectivity remain unchanged (entry 4). In the end, we decided to test an aliphatic acid (AcOH) but no variation of the yield or enantiomeric excess was verified (entry 5). Taking into consideration the achieved result, but considering that the

acidity of the additive could compromise the stability of the sulfoxonium ylide **2a** we decided to perform the same experiment but using sodium acetate as additive. In this case, an increase of yield was observed (entry 6). The beneficial effect of sodium acetate on the yield of the reaction was confirmed by an experiment performed without additives (entry 7). We then tried alternative bases, such as tertiary amines, as additives in the reaction. As shown in entries 8-11, use of these bases in either catalytic or stoichiometric amounts led to poorer results, so sodium acetate was chosen as co-catalyst for the reaction.

*Table S4. Additives screening*[a]

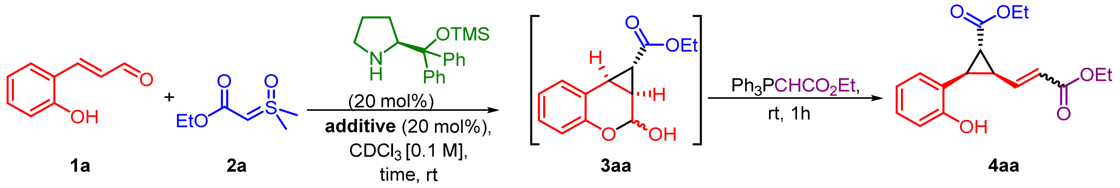


| entry | additive | ee [%][b] | Yield [%][c] | Time [h] |
|---|---|---|---|---|
| **1** | **PhCOOH** | 95 | 57 | 2 |
| **2** | **CSA** | 98 | 36 | 1 |
| **3** | **p-NO$_2$ benzoic acid** | *72* | 63 | 2 |
| **4** | **p-MeO benzoic acid** | 96 | 50 | 12 |
| **5** | **AcOH** | 96 | 52 | 12 |
| **6** | **AcONa** | 96 | 67 | 12 |
| **7** | **-** | 96 | 41 | 12 |
| **8** | **Et$_3$N** | 97 | 42 | 12 |
| **9** | **Et$_3$N (1 equiv.)** | 97 | 29 | 12 |
| **10** | ***i*-Pr$_2$EtN** | 93 | 40 | 12 |
| **11** | ***i*-Pr$_2$EtN (1 equiv.)** | 94 | 25 | 12 |

[a] Reaction conditions: **1a** (0.1 mmol, 1 equiv.), **2a** (0.15 mmol, 1.5 equiv.), additive (0.02 mmol, 20 mol%) catalyst **A** (0.02 mmol, 20 mol%) and $CDCl_3$ (1000 μL), rt, 1-12 h. [b] Enantiomeric excess determined by CSP-HPLC. [c] Yield determined after chromatographic column on silica gel.

## Proposed reaction pathway for the catalytic reaction

Scheme S1 summarizes our current hypothesis on the reaction pathway. Condensation of the cinnamaldehyde with the catalyst, under acidic conditions, affords a reactive iminium ion in equilibrium with its more stable (and unreactive) hemiaminal form. Attack of the sulfoxonium ylide on the less hindered rear face of the iminium ion results in an enamine intermediate. The enamine can displace DMSO by attacking with its rear face in a S*N*2-like reaction. The perfect diastereoselectivity observed in all cases can be ascribed either to a highly diastereoselective attack of the ylide to the imium ion, or to a reversible attack to the iminium ion followed by a selectivity determining DMSO displacement step. Hydrolysis releases the catalyst and an aldehyde product with 2,3-*trans* configuration. Epimerization α to the aldehyde function can be expected to be facile in this species. Hemiacetalization traps the *cis*-isomer giving stable compound **3aa**.

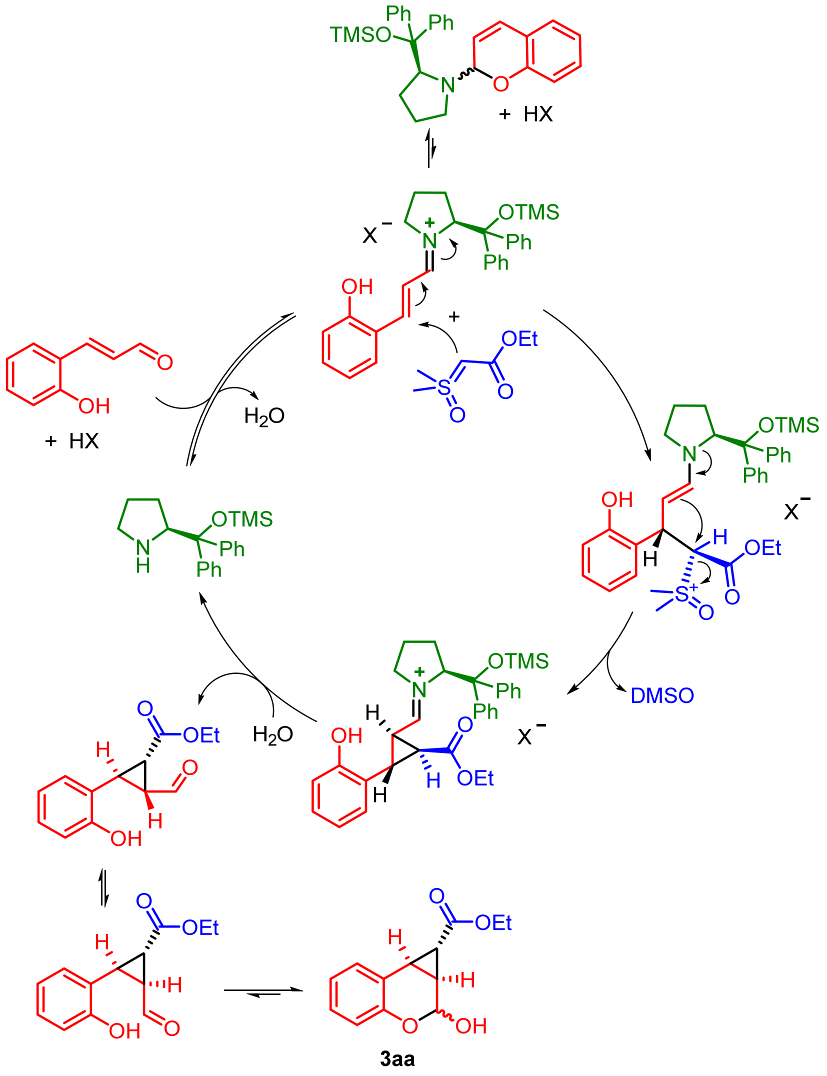


**Scheme S1**

## Additional experiments for the diastereodivergent oxa-Michael reaction 4aa → 8aa

The intramolecular oxa-Michael reaction delivering **8aa** from **4aa** was found to proceed smoothly under basic promotion. Since an achiral catalyst/promoter such as $Et_3N$ delivered the product with low diastereomeric ratio (1.5 : 1 favoring *trans*-**8aa**), few chiral bifunctional catalysts derived from *Cinchona* alkaloids were tried in the reaction in toluene at RT (Scheme S2), in order to develop a selective, and possibly diastereo-divergent, process. This class of catalysts is known to be very effective in a related oxa-Michael reaction.[1]

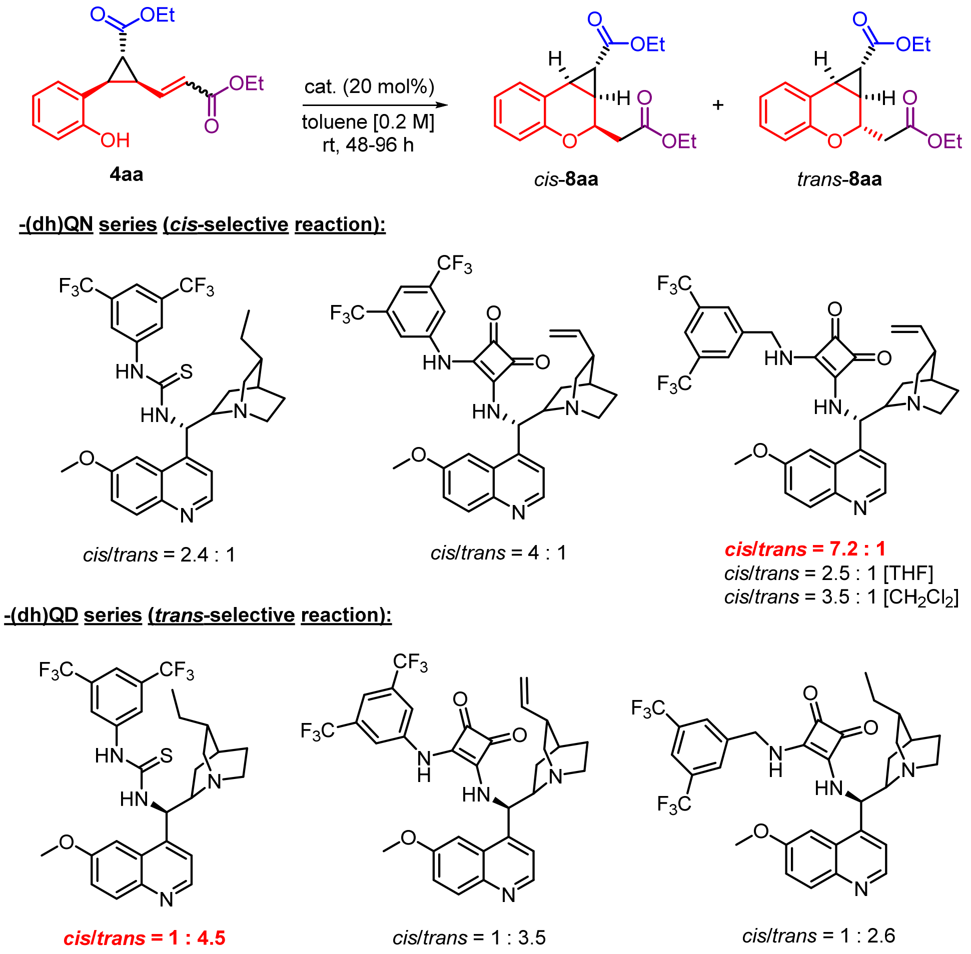


**Scheme S2**

Catalysts from the quinine series were found to promote the selective formation of the *cis*-**8aa** isomer, with a benzylic squaramide derivative outperforming other structures. Solvents other than toluene did not provide any improvement. Quinidine derived catalysts were indeed able to steer the reaction towards the *trans*-**8aa** isomer. However, in this case the catalyst providing the best result was found to be a thiourea derivative. The requirement of non-pseudoenantiomeric catalysts for a diastereo-

---

[1] Zhu, D.-X.; Liu, J.-G.; Xu, M.-H. *J. Am. Chem. Soc.* **2021**, *143*, 8583.

divergent process of this type can be rationalized considering that the transition states leading to *cis*-**8aa** and *trans*-**8aa** are intrinsically diastereomeric, and thus do not necessarily require enantiomeric catalysts for their stabilization/promotion.[2]

[2] a) Lotter, D.; Castrogiovanni, A.; Neuburger, M.; Sparr, C. *ACS Cent. Sci.* **2018**, *4*, 656. b) Corti, V.; Riccioli, R.; Martinelli, A.; Sandri, S.; Fochi, M.; Bernardi, L. *Chem. Sci.* **2021**, *12*, 10233.

## General methods and materials

**General Methods**. $^{1}H$ and $^{13}C$ NMR spectra were recorded on a Varian Mercury 300, 400 or Inova 600 spectrometer. Chemical shifts (δ) are reported in ppm relative to residual solvents signals for $^{1}H$ and $^{13}C$ NMR.[3] $^{13}C$ NMR were acquired with $^{1}H$ broad-band decoupled mode. NOE spectra were recorded using the DPFGSE-NOE sequence,[4] using a mixing time of 2.80 s and "rsnob" 50 Hz wide selective pulses. ECD spectra were recorded on a Jasco J-810 instrument. High Resolution Mass Spectra (HRMS) were recorded on a Waters Xevo Q-TOF spectrometer. ESI spectra were recorded on a micromass LCT spectrometer using electrospray (ESI) ionization technique. Compounds **4ba-ga** are rather unstable and could not be subjected to HRMS analysis, but only to low resolution ESI-MS (faster access). Optical rotations were measured on a Perkin Elmer 241 Polarimeter provided with a sodium lamp and are reported as follows: $[\alpha]_{\lambda}^{T\,(°C)}$ (c = g/100 mL, solvent). The enantiomeric excess of the products (ee) were determined by chiral stationary phase HPLC (Daicel Chiralpak OJ-H or AD-H or IC columns), using a UV detector operating at 254 nm. Infrared (ATR) spectra were recorded on a Perkin Elmer Spectrum Two FT-IR spectrometer equipped with an ATR probe. Signals are reported as strong (s), medium (m), and weak (w). Melting points (uncorrected) were determined with a Stuart Scientific SMP3 apparatus. Purification of reaction products was carried out by flash chromatography (FC) on silica gel (230-400 mesh) or by gravimetric chromatography using 70-230 mesh silica. The absolute and relative configuration of the products was determined on compounds **4'ab** and **3ab** (see dedicated section), and assigned by analogy to the remaining compounds. The relative configuration at the cyclopropane of known compound **6aa**, derived from **3aa**, is in line with this assignment.

**Materials**. Analytical grade solvents and commercially available reagents were used as received, unless otherwise stated. Catalyst **A** was purchased from Fluorochem and used as received. Reference racemic products **4** for CSP HPLC analysis were prepared using an equimolar mixture of (*R*)-**A** and (*S*)-**A** as catalyst. Catalysts **QN-1** and **dhQD-1** were prepared according to the literature.[5]

[3] Gottlieb, H. E.; Kottlyar, V.; Nudelman, A. *J. Org. Chem.* **1997**, *62*, 7512.
[4] (a) Stonehouse, J.; Adell, P.; Keeler, J.; Shaka, A. J. *J. Am. Chem. Soc.* **1994**, *116*, 6037. (b) Stott, K.; Stonehouse, J.; Keeler, J.; Hwang, T. L.; Shaka, A. J. *J. Am. Chem. Soc.* **1995**, *117*, 4199. (c) Stott, K.; Keeler, J.; Van, Q. N.; Shaka, A. J. *J. Magn. Reson.* **1997**, *125*, 302. (d) Van, Q. N.; Smith, E. M.; Shaka, A. J. *J. Magn. Reson.* **1999**, *141*, 191.
[5] Wang, Y.; Milikiewicz, K. L.; Kaufman, M. L.; He, L.; Landmesser, N. G.; Levy, D. V.; Allwein, S. P.; Christie, M. A.; Olsen, M. A.; Nelville, C. J.; Muthukumaran, K. *Org. Process Res. Dev.* **2017**, *21*, 408; Cassani, C; Martín-Rapún, R.; Arceo, E.; Bravo, F.; Melchiorre, P. *Nat. Protoc.* **2013**, *8*, 325; Malerich, J. P.; Hagihara, K.; Rawal, V. H. *J. Am. Chem. Soc.* **2008**, *130*, 14416.

# Starting Materials

## 2’-hydroxycinnamaldehydes 1

2’-hydroxycinnamaldehydes **1**, reported below, were prepared according to literature procedure.[6]

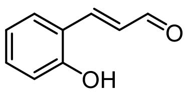
**1a**

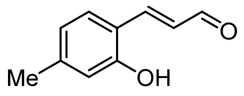
**1b**

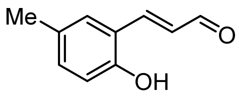
**1c**

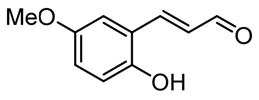
**1d**

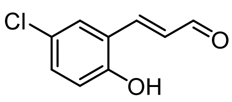
**1e**

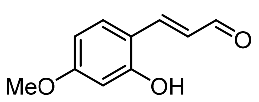
**1f**

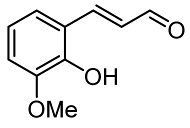
**1g**

## Sulfoxonium ylides 2

Sulfoxonium ylides **2**, reported below, were prepared according to literature procedure.[7]

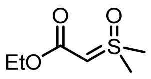
**2a**

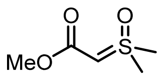
**2b**

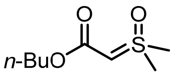
**2c**

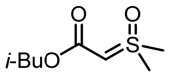
**2d**

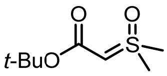
**2e**

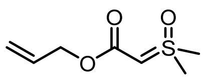
**2f**

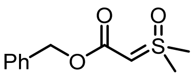
**2g**

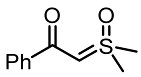
**2h**

[6] Ackrill, T. D.; Sparkes, H. A.; Wills, C. L. *Org. Lett.* **2015**, *17*, 3884-3887.
[7] Bisag, G. D.; Ruggieri, S.; Fochi, M.; Bernardi, L. *Adv. Synth. Catal.* **2021**, *363*, 3053-3059.

## Synthesis of products 4: general procedure and characterization

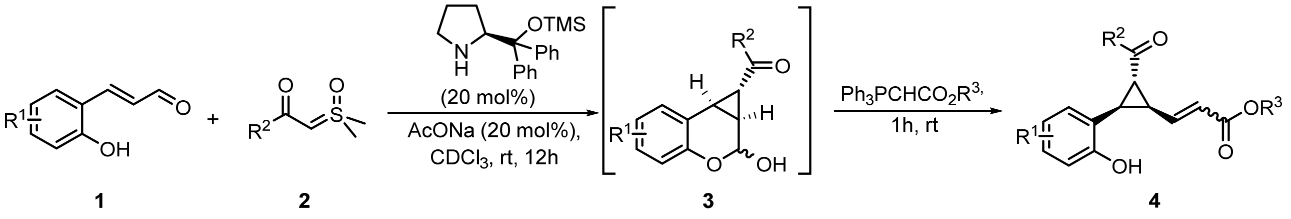

In a small vial equipped with a magnetic stirring bar, aldehyde **1** (0.1 mmol, 1 equiv.) and sulfoxonium ylide **2** (0.15 mmol, 1.5 equiv.) were added to a $CDCl_3$ (1 mL) solution of catalyst (*S*)-**A** (0.02 mmol, 0.20 equiv., 6.5 mg) and AcONa (0.02 mmol, 0.20 equiv., 1.6 mg). The resulting mixture was stirred at room temperature for 12 h. Subsequently, the appropriate phosphorous ylide (0.3 mmol, 3 equiv.) was added and the resulting mixture was stirred at room temperature for 1 h. Next, the solvent was eliminated under vacuum and directly purified by flash column chromatography on silica gel affording compounds **4** as E/Z mixtures. In some cases, a fraction containing compounds **4** as single E-isomers was collected and used for the characterization. In all cases, the E-**4** isomer was highly prevalent over its Z-**4** counterpart (estimated ratio >9:1).

**Ethyl (1*R*,2*S*,3*R*)-2-(3-ethoxy-3-oxoprop-1-en-1-yl)-3-(2-hydroxyphenyl)cyclopropane-1-carboxylate 4aa**

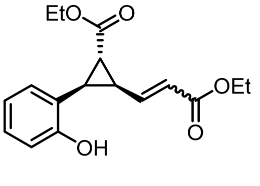

**4aa**

The general procedure was followed using aldehyde **1a**, sulfoxonium ylide **2a**, and ethyl 2-(triphenyl-$\lambda^5$-phosphaneylidene)acetate. Flash column chromatography ($CH_2Cl_2$/Acetone = 200:1) afforded a fraction containing **4aa** as E/Z mixture, and a fraction containing pure E-**4aa** as colorless oils (overall 67% yield, 20.4 mg). Performing the reaction on 1 mmol scale, that is, using 148.2 mg of substrate **1a** (1.0 mmol), 246.3 mg of ylide **2a** (1.5 mmol), 65.1 mg of catalyst (*S*)-**A** (0.20 mmol), 16.4 mg of sodium acetate (0.20 mmol) in 10 mL of $CDCl_3$ as solvent for the catalytic reaction, and 1.045 g of phosphorous ylide (3 mmol) for the Wittig reaction, product **4aa** was obtained in 69% overall yield (210.0 mg, 0.69 mmol) and 97% ee. E-**4aa** isomer: $[\alpha]_D^{25}$ = +20 (c = 0.5, $CH_2Cl_2$) for 97% ee. **IR** (ATR) ν(max) =3390 (br, m) 1714 (s) 1687 (s) 1176 (s) $cm^{-1}$. **$^1$H NMR** (400 MHz, $CDCl_3$, +25 °C) δ = 7.18 – 7.07 (m, 2H), 6.88 (t, J = 7.5 Hz, 1H), 6.78 (dd, J = 8.0, 1.2 Hz, 1H), 6.22 (dd, J = 15.5, 10.3 Hz, 1H), 6.02 (d, J = 15.5 Hz, 1H), 5.35 (s, 1H), 4.22 (q, J = 7.1 Hz, 2H), 4.10 (q, J = 7.1 Hz, 2H), 2.98 (dd, J = 9.2, 5.7 Hz, 1H), 2.61 (ddd, J = 10.4, 9.2, 4.4 Hz, 1H), 2.37 (dd, J = 5.7, 4.3 Hz, 1H), 1.31 (t, J = 7.1 Hz, 3H), 1.21 (t, J = 7.1 Hz, 3H). **$^{13}$C NMR** (101 MHz, $CDCl_3$, +25 °C) δ = 172.0, 166.0, 155.0, 144.9, 129.8, 128.9, 122.7, 120.9, 120.7, 115.5, 61.3, 60.3, 30.4, 28.4, 27.8, 14.25, 14.21. **HRMS** (ESI) m/z: $[M + Na]^+$ Calcd for $C_{17}H_{20}O_5Na$ 327.1203; Found 327.1196. **HPLC**: OJ-H (*n*-hexane/*i*-PrOH 90:10, 0.75 mL/min) $t_{min}$ = 15.1, min, $t_{maj}$ = 17.9 min.

**Methyl (1*R*,2*S*,3*R*)-2-(3-ethoxy-3-oxoprop-1-en-1-yl)-3-(2-hydroxyphenyl)cyclopropane-1-carboxylate 4ab**

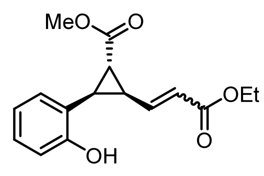


**4ab**

The general procedure was followed using aldehyde **1a**, sulfoxonium ylide **2b**, and ethyl 2-(triphenyl-$\lambda^5$-phosphaneylidene)acetate. Flash column chromatography ($CH_2Cl_2$/Acetone = 200:1) afforded a fraction containing E/Z mixture of **4ab** as colorless oil (64% yield, 18.6 mg) with 96% ee. E/Z-**4ab**: **IR** (ATR) ν(max) =3392 (br, m) 1710 (s) 1691 (s) 1248 (s) 1167 (s) 1139 (s) $cm^{-1}$. **$^1$H NMR** (600 MHz, $CDCl_3$, +25 °C) [signals of the E-isomer] δ = 7.14 (ddd, J = 8.1, 7.4, 1.7, Hz, 1H), 7.10 (d, J = 7.7 Hz, 1H), 6.87 (t, J = 7.5 Hz, 1H), 6.77 (dd, J = 8.0, 1.1 Hz, 1H), 6.20 (dd, J = 15.5, 10.4 Hz, 1H), 6.01 (d, J = 15.5 Hz, 1H), 5.12 (s, 1H), 4.09 (q, J = 7.1 Hz, 2H), 3.76 (s, 3H), 2.97 (dd, J = 9.1, 5.7 Hz, 1H), 2.61 (ddd, J = 10.3, 9.2, 4.3 Hz, 1H), 2.36 (dd, J = 5.7, 4.4 Hz, 1H), 1.20 (t, J = 7.1 Hz, 3H). **$^{13}$C NMR** (151 MHz, $CDCl_3$, +25 °C) [signals of the E-isomer] δ = 172.4, 165.9, 154.9, 144.7, 129.8, 128.9, 122.8, 120.8, 120.7, 115.5, 60.3, 52.3, 30.4, 28.4, 27.5, 14.2 **HRMS** (ESI) m/z: $[M + Na]^+$ Calcd for $C_{16}H_{18}O_5Na$ 313.1046; Found 313.1043; $[M + K]^+$ Calcd for $C_{16}H_{18}KO_5$ 329.0786; Found 329.0780. **HPLC**: IC (*n*-hexane/*i*-PrOH 80:20, 1 mL/min) E-isomer: $t_{min}$ = 7.7 min, $t_{maj}$ = 9.4 min.

**Methyl (1*R*,2*R*,3*S*)-2-(2-hydroxyphenyl)-3-(3-methoxy-3-oxoprop-1-en-1-yl)cyclopropane-1-carboxylate 4'ab**

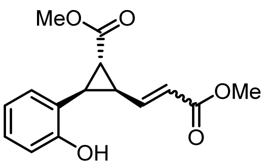


**4'ab**

The general procedure was followed using aldehyde **1a**, sulfoxonium ylide **2b,** and methyl 2-(triphenyl-$\lambda^5$-phosphaneylidene)acetate. Flash column chromatography ($CH_2Cl_2$/Acetone = 200:1) afforded a fraction containing E/Z mixture of **4'ab** as colorless oil in 62% yield (17.1 mg) with 94% ee. E/Z-**4'ab**: **IR** (ATR) ν(max) =3400 (br, m) 1719 (s) 1694 (s) 1251 (s) 1160 (s) 1139 (s) $cm^{-1}$. **$^1$H NMR** (600 MHz, $CDCl_3$, +25 °C) [signals of the E-isomer] δ = 7.15 (td, *J* = 7.8, 1.6 Hz, 1H), 7.10 (dd, *J* = 6.3, 4.8 Hz, 1H), 6.90 – 6.85 (m, 1H), 6.78 (dd, *J* = 8.0, 1.1 Hz, 1H), 6.20 (dd, *J* = 15.5, 10.4 Hz, 1H), 6.01 (d, *J* = 15.4 Hz, 1H), 5.09 (s, 1H), 3.76 (s, 3H), 3.63 (s, 3H), 3.01 – 2.94 (m, 1H), 2.61 (ddd, *J* = 10.2, 9.1, 4.3 Hz, 1H), 2.36 (dd, *J* = 5.7, 4.4 Hz, 1H). **$^{13}$C NMR** (151 MHz, $CDCl_3$, +25 °C) [signals of the E-isomer] δ = 172.3, 166.3, 154.9, 145.0, 129.9, 128.9, 122.4, 120.83, 120.81, 115.5, 52.3, 51.5, 30.4, 28.5, 27.6. **HRMS** (ESI) m/z: $[M + Na]^+$ Calcd for $C_{15}H_{16}O_5Na$ 299.0890; Found 299.0886; $[M + K]^+$ Calcd for $C_{15}H_{16}KO_5$ 315.0629; Found 315.0637. **HPLC**: ADH (*n*-hexane/*i*-PrOH 90:10, 0.75 mL/min) E-isomer: $t_{maj}$ = 18.2 min, $t_{min}$ = 19.6 min.

**Butyl (1*R*,2*S*,3*R*)-2-(-3-ethoxy-3-oxoprop-1-en-1-yl)-3-(2-hydroxyphenyl)cyclopropane-1-carboxylatecarboxylate 4ac**

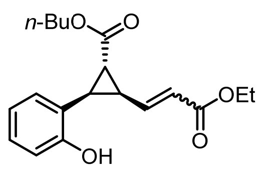


**4ac**

The general procedure was followed using aldehyde **1a**, sulfoxonium ylide **2c,** and ethyl 2-(triphenyl-$\lambda^5$-phosphaneylidene)acetate. Flash column chromatography ($CH_2Cl_2$/Acetone = 200:1) afforded a fraction containing **4ac** as E/Z mixture, and a fraction containing pure E-**4ac** as colorless oils (overall 60% yield, 19.9 mg) with 95% ee. E-**4ac** isomer: $[\alpha]_D^{25}$ = +28 (c = 0.5, $CH_2Cl_2$) for 95% ee. **IR** (ATR) ν(max) =3398 (br, m) 1718 (s) 1693 (s) 1248 (s) 1168 (s) 1139 (s) $cm^{-1}$.**$^1$H NMR** (600 MHz, $CDCl_3$, +25 °C) δ = 7.17 – 7.06 (m, 2H), 6.87 (td, J = 7.5, 1.2 Hz, 1H), 6.77 (dd, J = 8.1, 1.2 Hz, 1H), 6.21 (dd, J = 15.5, 10.4 Hz, 1H), 6.01 (d, J = 15.4 Hz, 1H), 5.29 (s, 1H), 4.15 (t, J = 6.7 Hz, 2H), 4.09 (q, J = 7.1 Hz, 2H), 2.96 (dd, J = 9.1, 5.7 Hz, 1H), 2.60 (ddd, J = 10.3, 9.1, 4.3 Hz, 1H), 2.36 (dd, J = 5.7, 4.3 Hz, 1H), 1.72 – 1.56 (m, 2H), 1.46 – 1.34 (m, 2H), 1.20 (t, J = 7.1 Hz, 3H), 0.94 (t, J = 7.4 Hz, 3H). **$^{13}$C NMR** (101 MHz, $CDCl_3$, +25 °C) δ = 172.1, 166.0, 155.0, 144.9, 129.8, 128.9, 122.7, 120.9, 120.7, 115.5, 65.2, 60.3, 30.6, 30.3, 28.4, 27.8, 19.1, 14.1, 13.7. **HRMS** (ESI) m/z: $[M + Na]^+$ Calcd for $C_{19}H_{24}O_5Na$ 355.1516; Found 355.1508. **HPLC**: OJ-H (*n*-hexane/*i*-PrOH 90:10, 0.75 mL/min) $t_{min}$ = 10.6 min, $t_{maj}$ = 12.4 min.

**Isobutyl (1*R*,2*S*,3*R*)-2-(-3-ethoxy-3-oxoprop-1-en-1-yl)-3-(2-hydroxyphenyl)cyclopropane-1-carboxylate 4ad**

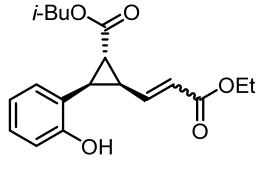


**4ad**

The general procedure was followed using aldehyde **1a**, sulfoxonium ylide **2d**, and ethyl 2-(triphenyl-$\lambda^5$-phosphaneylidene)acetate. Flash column chromatography ($CH_2Cl_2$/Acetone = 200:1) afforded a fraction containing **4ad** as E/Z mixture, and a fraction containing pure E-**4ad** as colorless oils (overall 70% yield, 23.2 mg) with 95% ee. E-**4ad** isomer: $[\alpha]_D^{25}$ = +27 (c = 0.5, $CH_2Cl_2$) for 95% ee. **IR** (ATR) ν(max) =3396 (br, m) 1718 (s) 1693 (s) 1247 (s) 1163 (s) 1139 (s) $cm^{-1}$. **$^1$H NMR** (600 MHz, $CDCl_3$, +25 °C) δ = 7.17 – 7.05 (m, 2H), 6.87 (td, J = 7.5, 1.2 Hz, 1H), 6.77 (dd, J = 8.1, 1.2 Hz, 1H), 6.22 (dd, J = 15.5, 10.4 Hz, 1H), 6.01 (d, J = 15.4 Hz, 1H), 5.30 (s, 1H), 4.09 (q, J = 7.1 Hz, 2H), 3.93 (d, J = 6.6 Hz, 2H), 2.97 (dd, J = 9.2, 5.7 Hz, 1H), 2.61 (ddd, J = 10.4, 9.1, 4.4 Hz, 1H), 2.37 (dd, J = 5.7, 4.4 Hz, 1H), 2.01-1.97 (m, 1H), 1.20 (t, J = 7.1 Hz, 3H), 0.95 (d, J = 6.7 Hz, 6H). **$^{13}$C NMR** (101 MHz, $CDCl_3$, +25 °C) δ = 172.0, 166.0, 155.0, 144.9, 129.8, 128.9, 122.7, 120.9, 120.7, 115.5, 71.4, 60.3, 30.3, 28.4, 27.8, 27.7, 19.1, 14.1. **HRMS** (ESI) m/z: $[M + Na]^+$ Calcd

for $C_{19}H_{24}O_5Na$ 355.1516; Found 355.1515. **HPLC**: OJ-H (*n*-hexane/*i*-PrOH 90:10, 0.75 mL/min) $t_{min}$ = 8.9 min, $t_{maj}$ = 10.0 min.

***tert*-Butyl (1*R*,2*S*,3*R*)-2-(-3-ethoxy-3-oxoprop-1-en-1-yl)-3-(2-hydroxyphenyl)cyclopropane-1-carboxylate 4ae**

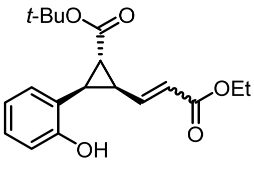


**4ae**

The general procedure was followed using aldehyde **1a**, sulfoxonium ylide **2e**, and ethyl 2-(triphenyl-$\lambda^5$-phosphaneylidene)acetate. Flash column chromatography ($CH_2Cl_2$/Acetone = 200:1) afforded a fraction containing **4ae** as E/Z mixture, and a fraction containing pure E-**4ae** as colorless oils (overall 57% yield, 19.0 mg) with 95% ee. E-**4ae** isomer: $[\alpha]_D^{25}$ = +25 (c = 0.4, $CH_2Cl_2$) for 95% ee. **IR** (ATR) ν(max) =3423 (br, m) 1710 (s) 1698 (s) 1254 (s) 1141 (s) $cm^{-1}$. **$^1$H NMR** (600 MHz, $CDCl_3$, +25 °C) δ = 7.17 – 7.06 (m, 2H), 6.87 (td, *J* = 7.5, 1.2 Hz, 1H), 6.78 (dd, *J* = 7.9, 1.2 Hz, 1H), 6.20 (dd, *J* = 15.5, 10.4 Hz, 1H), 6.00 (d, *J* = 15.5 Hz, 1H), 5.29 (s, 1H), 4.08 (q, *J* = 7.1 Hz, 2H), 2.89 (dd, *J* = 9.1, 5.7 Hz, 1H), 2.53 (ddd, *J* = 10.4, 9.2, 4.4 Hz, 1H), 2.28 (dd, *J* = 5.8, 4.4 Hz, 1H), 1.48 (s, 9H), 1.19 (t, *J* = 7.1 Hz, 3H). **$^{13}$C NMR** (101 MHz, $CDCl_3$, +25 °C) δ = 171.0, 166.0, 155.0, 145.1, 129.8, 128.8, 122.6, 121.0, 120.6, 115.5, 81.6, 60.2, 30.0, 28.8, 28.1, 28.0, 14.2. **HRMS** (ESI) m/z: $[M + Na]^+$ Calcd for $C_{19}H_{24}O_5Na$ 355.1516; Found 355.1514. **HPLC**: OJ-H (*n*-hexane/*i*-PrOH 90:10, 0.75 mL/min) $t_{min}$ = 7.1 min, $t_{maj}$ = 7.8 min.

**Allyl (1*R*,2*S*,3*R*)-2-(3-ethoxy-3-oxoprop-1-en-1-yl)-3-(2-hydroxyphenyl)cyclopropane-1-carboxylate 4af**

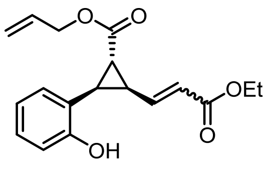


**4af**

The general procedure was followed using aldehyde **1a**, sulfoxonium ylide **2f**, and ethyl 2-(triphenyl-$\lambda^5$-phosphaneylidene)acetate. Flash column chromatography ($CH_2Cl_2$/Acetone = 200:1) afforded a fraction containing **4af** as E/Z mixture, and a fraction containing pure E-**4af** as colorless oils (overall 65% yield, 20.5 mg) with 90% ee. E-**4af** isomer: $[\alpha]_D^{25}$ +31 (c = 0.3, $CH_2Cl_2$) for 90% ee. **IR** (ATR) ν(max) =3393 (br, m) 1715 (s) 1691 (s) 1249 (s) 1160 (s) 1139 (s) $cm^{-1}$. **$^1$H NMR** (600 MHz, $CDCl_3$, +25 °C) δ = 7.19 – 7.06 (m, 2H), 6.87 (td, *J* = 7.5, 1.2 Hz, 1H), 6.77 (dd, *J* = 8.0, 1.2 Hz, 1H), 6.21 (dd, *J* = 15.5, 10.3 Hz, 1H), 6.01 (d, *J* = 15.5 Hz, 1H), 5.94 (ddt, *J* = 17.1, 10.4, 5.8 Hz, 1H), 5.39 – 5.31 (m, 1H), 5.27 (dq, *J* = 10.4, 1.2 Hz, 1H), 5.23 (s, 1H), 4.65 (dt, *J* = 5.9, 1.4 Hz, 2H), 4.09 (q, *J* = 7.1 Hz, 2H), 2.99 (dd, *J* = 9.2, 5.7 Hz, 1H), 2.62 (ddd, *J* = 10.3, 9.1, 4.3 Hz, 1H), 2.39 (dd, *J* = 5.7, 4.4 Hz, 1H), 1.20 (t, *J* = 7.1 Hz, 3H). **$^{13}$C NMR** (101 MHz, $CDCl_3$, +25 °C) δ = 171.7, 165.9, 154.9, 144.7, 131.8, 129.8, 128.9, 122.8, 120.8, 120.7, 118.7, 115.5, 65.9, 60.3, 30.5,

28.5, 27.7, 14.1. **HRMS** (ESI) m/z: [M + Na]$^+$ Calcd for $C_{18}H_{20}O_5Na$ 339.1203; Found 339.1210. **HPLC**: OJ-H (*n*-hexane/*i*-PrOH 90:10, 0.75 mL/min) $t_{min}$ = 15.3 min, $t_{maj}$ = 19.1 min.

### Benzyl (1*R*,2*S*,3*R*)-2-(3-ethoxy-3-oxoprop-1-en-1-yl)-3-(2-hydroxyphenyl)cyclopropane-1-carboxylate 4ag

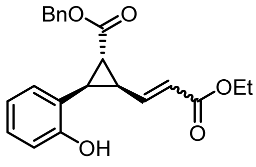


**4ag**

The general procedure was followed using aldehyde **1a**, sulfoxonium ylide **2g** and ethyl 2-(triphenyl-λ$^5$-phosphaneylidene)acetate. Flash column chromatography ($CH_2Cl_2$/Acetone = 200:1) afforded a fraction containing **4ag** as E/Z mixture, and a fraction containing pure E-**4ag** as colorless oils (overall 56% yield, 10.5 mg) with 97% ee. E-**4ag** isomer: $[\alpha]_D^{25}$ = +11 (c = 0.4, $CH_2Cl_2$) for 97% ee. **IR** (ATR) ν(max) =3388 (br, m) 1714 (s) 1690 (s) 1248 (s) 1161 (s) 1138 (s) cm$^{-1}$. **$^1$H NMR** (600 MHz, $CDCl_3$, +25 °C) δ = 7.41 – 7.29 (m, 5H), 7.17 – 7.06 (m, 2H), 6.86 (td, *J* = 7.5, 1.2 Hz, 1H), 6.76 (dd, *J* = 8.1, 1.1 Hz, 1H), 6.20 (dd, *J* = 15.5, 10.3 Hz, 1H), 6.00 (d, *J* = 15.5 Hz, 1H), 5.19 (br s, 2H), 4.08 (q, *J* = 7.1 Hz, 2H), 3.00 (dd, *J* = 9.2, 5.7 Hz, 1H), 2.63 (ddd, *J* = 10.4, 9.2, 4.3 Hz, 1H), 2.42 (dd, *J* = 5.7, 4.3 Hz, 1H), 1.19 (t, *J* = 7.1 Hz, 3H). **$^{13}$C NMR** (101 MHz, $CDCl_3$, +25 °C) δ = 171.8, 165.9, 154.9, 144.7, 135.5, 129.8, 128.9, 128.6, 128.4, 128.3, 122.9, 120.8, 120.7, 115.5, 67.1, 60.3, 30.5, 28.6, 27.7, 14.1. **HRMS** (ESI) m/z: [M + Na]$^+$ Calcd for $C_{22}H_{22}O_5Na$ 389.1359; Found 389.1357. **HPLC**: OJ-H (*n*-hexane/*i*-PrOH 90:10, 0.75 mL/min) $t_{min}$ = 27.3 min, $t_{maj}$ = 38.7 min.

### Ethyl 3-((1*S*,2*R*,3*R*)-2-benzoyl-3-(2-hydroxyphenyl)cyclopropyl)acrylateacrylate 4ah

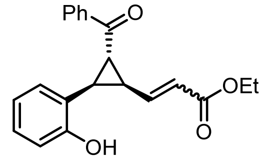


**4ah**

The general procedure was followed using aldehyde **1a**, sulfoxonium ylide **2h**, and ethyl 2-(triphenyl-λ$^5$-phosphaneylidene)acetate. Flash column chromatography ($CH_2Cl_2$/Acetone = 200:1) afforded a fraction containing **4ah** as E/Z mixture as colorless oil (35% yield, 12.4 mg) with 93% ee. E/Z-**4ah** mixture: **IR** (ATR) ν(max) =3431 (br, m) 1700 (s) 1661 (s) 1253 (s) 1140 (s) cm$^{-1}$. **$^1$H NMR** (600 MHz, $CDCl_3$, +25 °C) [signals of the E-isomer] δ = 8.09 – 8.05 (m, 2H), 7.65 – 7.57 (m, 1H), 7.55 – 7.48 (m, 2H), 7.20-7.17 (m, 2H), 6.91 (td, *J* = 7.5, 1.2 Hz, 1H), 6.82 (dd, *J* = 8.5, 1.2 Hz, 1H), 6.41 (dd, *J* = 15.5, 10.5 Hz, 1H), 6.06 (d, *J* = 15.5 Hz, 1H), 5.24 (s, 1H), 4.12 (q, *J* = 7.1 Hz, 2H), 3.38 (dd, *J* = 5.6, 4.2 Hz, 1H), 3.23 (dd, *J* = 8.9, 5.6 Hz, 1H), 2.81 (ddd, *J* = 10.6, 8.9, 4.2 Hz, 1H), 1.29 – 1.15 (m, 3H). **$^{13}$C NMR** (101 MHz, $CDCl_3$, +25 °C) [signals of the E-isomer] δ = 197.1, 166.1, 155.0, 145.2, 137.3, 133.4, 130.0, 129.0, 128.8, 128.3, 122.7, 121.3, 120.8, 115.7, 60.3, 33.5, 32.2, 30.7, 14.2. **HRMS** (ESI) m/z: [M + Na]$^+$ Calcd for $C_{21}H_{20}O_4Na$ 359.1254; Found

359.1253. **HPLC**: OJ-H (*n*-hexane/*i*-PrOH 90:10, 0.75 mL/min, E-isomer) $t_{min}$ = 30.7 min, $t_{maj}$ = 42.6 min.

**Ethyl (1*R*,2*S*,3*R*)-2-(3-ethoxy-3-oxoprop-1-en-1-yl)-3-(2-hydroxy-4-methylphenyl)cyclopropane-1-carboxylate 4ba**

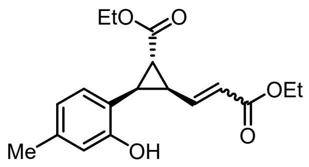


**4ba**

The general procedure was followed using aldehyde **1b**, sulfoxonium ylide **2a**, and ethyl 2-(triphenyl-$\lambda^5$-phosphaneylidene)acetate. Flash column chromatography ($CH_2Cl_2$/Acetone = 200:1) afforded a fraction containing **4ba** as E/Z mixture, and a fraction containing pure E-**4ba** as colorless oils (overall 52% yield, 16.5 mg) with 92% ee. E-**4ba** isomer: $[\alpha]_D^{25}$ +38 (c = 0.3, $CH_2Cl_2$) for 92% ee. **IR** (ATR) ν(max) =3396 (br, m) 1715 (s) 1693 (s) 1251 (s) 1173 (s) 1139 (s) $cm^{-1}$. **$^1$H NMR** (400 MHz, $CDCl_3$, +25 °C) δ = 7.02 – 6.93 (m, 1H), 6.69 (dt, *J* = 7.7, 1.1 Hz, 1H), 6.61 (s, 1H), 6.22 (dd, *J* = 15.5, 10.4 Hz, 1H), 6.02 (d, *J* = 15.5 Hz, 1H), 5.06 (s, 1H), 4.28 – 4.18 (m, 2H), 4.11 (q, *J* = 7.1 Hz, 2H), 2.93 (dd, *J* = 9.1, 5.6 Hz, 1H), 2.58 (ddd, *J* = 10.4, 9.1, 4.3 Hz, 1H), 2.33 (dd, *J* = 5.7, 4.3 Hz, 1H), 2.28 (s, 3H), 1.31 (t, *J* = 7.1 Hz, 3H), 1.22 (t, *J* = 7.1 Hz, 3H). **$^{13}$C NMR** (101 MHz, $CDCl_3$, +25 °C) δ = 172.0, 166.0, 154.7, 145.0, 139.1, 129.6, 122.7, 121.5, 117.7, 116.2, 61.3, 60.2, 30.4, 28.2, 27.8, 21.1, 14.25, 14.22. **MS** (ESI) m/z: $[M + Na]^+$ 341. **HPLC**: IC (*n*-hexane/*i*-PrOH 80:20, 1 mL/min) $t_{min}$ = 7.1 min, $t_{maj}$ = 8.8 min.

**Ethyl (1*R*,2*S*,3*R*)-2-(3-ethoxy-3-oxoprop-1-en-1-yl)-3-(2-hydroxy-5-methylphenyl)cyclopropane-1-carboxylate 4ca**

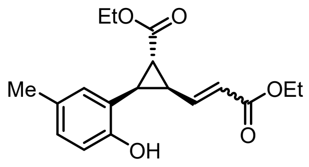


**4ca**

The general procedure was followed using aldehyde **1c**, sulfoxonium ylide **2a**, and ethyl 2-(triphenyl-$\lambda^5$-phosphaneylidene)acetate. Flash column chromatography ($CH_2Cl_2$/Acetone = 200:1) afforded a fraction containing E/Z mixture of **4ca** as colorless oil (overall 45% yield, 14.3 mg) and 95% ee. E/Z-**4ca**: **IR** (ATR) ν(max) =3433 (br, m) 1703 (s) 1700 (s) 1251 (s) 1180 (s) 1138 (s) $cm^{-1}$. **$^1$H NMR** (600 MHz, $CDCl_3$, +25 °C) [signals of the E-isomer] δ = 6.98 – 6.86 (m, 2H), 6.67 (d, *J* = 8.1 Hz, 1H), 6.20 (dd, *J* = 15.5, 10.4 Hz, 1H), 6.03 (dd, *J* = 23.6, 15.5 Hz, 1H), 4.94 (s, 1H), 4.26 – 4.15 (m, 2H), 4.09 (q, *J* = 7.1 Hz, 2H), 3.00 – 2.89 (m, 1H), 2.58 (ddd, *J* = 10.5, 9.1, 4.3 Hz, 1H), 2.35 (dd, *J* = 5.7, 4.3 Hz, 1H), 2.24 (s, 3H), 1.33 – 1.26 (m, 3H), 1.20 (t, *J* = 7.1 Hz, 3H). **$^{13}$C NMR** (101 MHz, $CDCl_3$, +25 °C) [signals of the E-isomer] δ = 172.0, 165.9, 152.6, 144.9, 130.3, 129.9, 129.3, 122.7, 115.4, 61.3, 60.2, 30.4, 28.5, 27.7, 20.5, 14.27, 14.23. **MS** (ESI)

m/z: $[M + Na]^+$ 341. **HPLC**: IC (*n*-hexane/*i*-PrOH 80:20, 1 mL/min) E-isomer: $t_{min}$ = 6.9 min, $t_{maj}$ = 8.6 min.

**Ethyl (1*R*,2*S*,3*R*)-2-(-3-ethoxy-3-oxoprop-1-en-1-yl)-3-(2-hydroxy-5-methoxyphenyl)cyclopropane-1-carboxylate 4da**

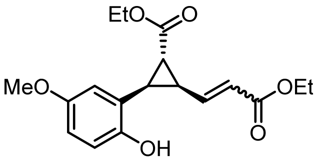

**4da**

The general procedure was followed using aldehyde **1d**, sulfoxonium ylide **2a**, and ethyl 2-(triphenyl-$\lambda^5$-phosphaneylidene)acetate. Flash column chromatography ($CH_2Cl_2$/Acetone = 200:1) afforded a fraction containing E/Z mixture of **4da** as colorless oil (overall 63% yield, 21.1 mg), and 97% ee. E/Z-**4da**: **IR** (ATR) ν(max) =3409 (br, m) 1714 (s) 1695 (s) 1251 (s) 1199 (s) 1176 (s) $cm^{-1}$. **$^1$H NMR** (600 MHz, $CDCl_3$, +25 °C) [signals of the E-isomer] δ = 6.80 – 6.60 (m, 3H), 6.23 (dd, *J* = 15.5, 10.3 Hz, 1H), 6.03 (d, *J* = 15.5 Hz, 1H), 4.82 (s, 1H), 4.22 (qd, *J* = 7.2, 0.6 Hz, 2H), 4.11 (q, *J* = 7.1 Hz, 2H), 3.75 (s, 3H), 2.99 – 2.93 (m, 1H), 2.60 (ddd, *J* = 10.4, 9.2, 4.3 Hz, 1H), 2.34 (dd, *J* = 5.7, 4.4 Hz, 1H), 1.31 (t, *J* = 7.1 Hz, 3H), 1.22 (t, *J* = 7.1 Hz, 3H). **$^{13}$C NMR** (101 MHz, $CDCl_3$, +25 °C) [signals of the E-isomer] δ = 171.8, 165.9, 153.5, 148.9, 144.7, 122.9, 121.9, 116.3, 115.6, 113.8, 61.3, 60.3, 55.8, 30.3, 28.6, 27.8, 14.25, 14.22. **MS** (ESI) m/z: $[M + Na]^+$ 357. **HPLC**: IC (*n*-hexane/*i*-PrOH 80:20, 1 mL/min) E-isomer: $t_{min}$ = 10.2 min, $t_{maj}$ = 11.7 min.

**Ethyl (1*R*,2*R*,3*S*)-2-(5-chloro-2-hydroxyphenyl)-3-(-3-ethoxy-3-oxoprop-1-en-1-yl)cyclopropane-1-carboxylate 4ea**

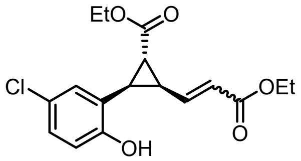

**4ea**

The general procedure was followed using aldehyde **1e**, sulfoxonium ylide **2a**, and ethyl 2-(triphenyl-$\lambda^5$-phosphaneylidene)acetate. Flash column chromatography ($CH_2Cl_2$/Acetone = 200:1) afforded a fraction containing **4ea** as E/Z mixture, and a fraction containing pure E-**4ea** as pale yellow oils (overall 57% yield, 19.3 mg) with 90% ee. E-**4ea**: $[\alpha]_D^{25}$ = +38 (c = 0.3, $CH_2Cl_2$) for 90% ee. **IR** (ATR) ν(max) = 3418 (br, m) 1704 (s) 1249 (s) 1179 (s) 1140 (s) $cm^{-1}$. **$^1$H NMR** (600 MHz, $CDCl_3$, +25 °C) [signals of the E-isomer] δ = 7.15 – 7.04 (m, 2H), 6.73 – 6.66 (m, 1H), 6.17 (dd, *J* = 15.4, 10.3 Hz, 1H), 6.01 (d, *J* = 15.5 Hz, 1H), 5.50 (s, 1H), 4.25-4.19 (m, 2H), 4.10 (q, *J* = 7.1 Hz, 2H), 2.92 (dd, *J* = 9.2, 5.7 Hz, 1H), 2.59 (ddd, *J* = 10.3, 9.1, 4.4 Hz, 1H), 2.34 (dd, *J* = 5.7, 4.4 Hz, 1H), 1.31 (t, *J* = 7.1 Hz, 3H), 1.21 (t, *J* = 7.1 Hz, 3H). **$^{13}$C NMR** (151 MHz, $CDCl_3$, +25 °C) [signals of the E-isomer] δ = 171.7, 166.0, 153.7, 144.2, 129.7, 128.7, 125.3, 123.2, 122.8, 116.8, 61.5, 60.4, 30.2, 28.1, 27.5, 14.2, 14.1. **MS** (ESI) m/z: $[M(^{35}Cl) + Na]^+$ 361, $[M(^{37}Cl) + Na]^+$ 363. **HPLC**: IC (*n*-hexane/*i*-PrOH 80:20, 1 mL/min) E-isomer: $t_{min}$ = 5.1 min, $t_{maj}$ = 5.9 min.

**Ethyl (1R,2S,3R)-2-(3-ethoxy-3-oxoprop-1-en-1-yl)-3-(2-hydroxy-4-methoxyphenyl)cyclopropane-1-carboxylate 4fa**

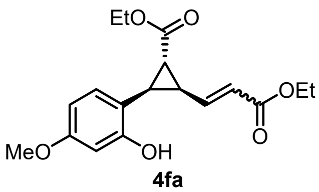

4fa

The general procedure was followed using aldehyde **1f**, sulfoxonium ylide **2b** and ethyl 2-(triphenyl-$\lambda^5$-phosphaneylidene)acetate and 1 equiv. of NaOAc (8.2 mg). Flash column chromatography ($CH_2Cl_2$/Acetone = 200:1) afforded a fraction containing E/Z mixture of **4fa** as yellow oil (43% yield, 14.4 mg), and 85% ee . E-**4fa**: $[\alpha]_D^{25}$ = +23 (c = 0.4, $CH_2Cl_2$) for 85% ee. **IR** (ATR) ν(max) = 3393 (br, m) 1715 (s) 1695 (s) 1162 (s) $cm^{-1}$. **$^1$H NMR** (600 MHz, $CDCl_3$, +25 °C) [signals of the E-isomer] δ = 7.01 (dd, *J* = 8.4, 0.9 Hz, 1H), 6.44 (dd, *J* = 8.5, 2.5 Hz, 1H), 6.38 (d, *J* = 2.5 Hz, 1H), 6.23 (dd, *J* = 15.5, 10.3 Hz, 1H), 6.02 (d, *J* = 15.5 Hz, 1H), 5.75 (s, 1H), 4.26 – 4.16 (m, 2H), 4.16 – 4.06 (m, 2H), 3.76 (s, 3H), 2.88 (dd, *J* = 8.9, 5.5 Hz, 1H), 2.57 (ddd, *J* = 10.4, 9.0, 4.3 Hz, 1H), 2.31 (dd, *J* = 5.6, 4.3 Hz, 1H), 1.31 (t, *J* = 7.1 Hz, 3H), 1.22 (t, *J* = 7.1 Hz, 3H). **$^{13}$C NMR** (151 MHz, $CDCl_3$, +25 °C) [signals of the E-isomer] δ = 171.9, 165.9, 160.3, 155.8, 144.8, 130.6, 122.7, 113.0, 106.2, 101.7, 61.3, 60.2, 55.3, 30.2, 27.9, 27.8, 14.24, 14.20. **MS** (ESI) m/z: $[M + Na]^+$ 357. **HPLC**: AD-H (*n*-hexane/*i*-PrOH 90:10, 1 mL/min) E-isomer: $t_{maj}$ = 16.7 min, $t_{min}$ = 22.8 min.

**Ethyl (1*R*,2*S*,3*R*)-2-(3-ethoxy-3-oxoprop-1-en-1-yl)-3-(2-hydroxy-3-methoxyphenyl)cyclopropane-1-carboxylate 4ga**

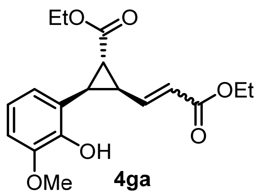

4ga

The general procedure was followed using aldehyde **1g**, sulfoxonium ylide **2a**, and ethyl 2-(triphenyl-$\lambda^5$-phosphaneylidene)acetate. Flash column chromatography ($CH_2Cl_2$/Acetone = 200:1) afforded a fraction containing E/Z mixture of **4ga** as colorless oil (overall 43% yield 14.4 mg) and 88% ee. E/Z-**4ga**: **IR** (ATR) ν(max) = 3427 (br, m) 1711 (s) 1272 (s) 1176 (s) $cm^{-1}$. **$^1$H NMR** (600 MHz, $CDCl_3$, +25 °C) [signals of the E-isomer] δ = 6.81 – 6.73 (m, 2H), 6.73 – 6.66 (m, 1H), 6.26 (dd, *J* = 15.5, 10.4 Hz, 1H), 5.99 (d, *J* = 15.4 Hz, 1H), 5.74 (s, 1H), 4.21 – 4.15 (m, 2H), 4.08 (q, *J* = 7.1 Hz, 2H), 3.86 (s, 3H), 3.06 (dd, *J* = 9.3, 5.8 Hz, 1H), 2.58 (ddd, *J* = 10.5, 9.4, 4.4 Hz, 1H), 2.38 (dd, *J* = 5.8, 4.4 Hz, 1H), 1.29 (t, *J* = 7.1 Hz, 3H), 1.20 (t, *J* = 7.1 Hz, 3H). **$^{13}$C NMR** (151 MHz, $CDCl_3$, +25 °C) [signals of the E-isomer] δ = 172.1, 166.1, 146.4, 145.6, 145.0, 122.3, 121.4, 120.7, 119.3, 109.8, 61.1, 60.1, 56.0, 30.7, 28.6, 27.7, 14.22, 14.20. **MS** (ESI) m/z: $[M + Na]^+$ 357. **HPLC**: IC (*n*-hexane/*i*-PrOH 80:20, 1 mL/min) E-isomer: $t_{maj}$ = 12.5 min, $t_{min}$ = 15.2 min.

## Synthetic elaborations

Synthetic elaboration on **3aa** were performed using freshly prepared **3aa**, isolated by a fast flash column chromatography using 3:1 hexane/acetone from catalytic crude, or using the one pot protocols detailed below.

**Ethyl (1*S*,1a*S*,7b*S*)-2-oxo-1,1a,2,7b-tetrahydrocyclopropa[*c*]chromene-1-carboxylate 5aa**

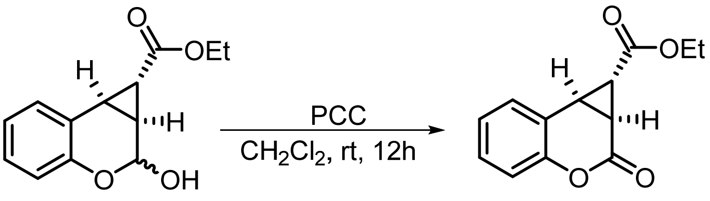


**3aa** **5aa**

In a small vial equipped with a magnetic stirring bar, PCC (0.2 mmol, 2 equiv., 43 mg) was added to a solution of cyclopropanchromanol **3aa** (0.1 mmol, 1 equiv., 23 mg), in $CH_2Cl_2$ (0.5 mL). The resulting solution was stirred at room temperature for 12 h and then poured into an aq. solution of $Na_2SO_3$ (3 M), and extracted with DCM (3 x). The combined organic phases were dried over $MgSO_4$, filtered and evaporated under reduced pressure. The crude residue was then purified by flash column chromatography on silica gel (*n*-hexane/$Et_2O$ = 3:1) affording product **5aa** as a white solid in 37% yield (8.6 mg)

*One pot protocol:* In a small vial equipped with a magnetic stirring bar, aldehyde **1a** (0.1 mmol, 1 equiv., 14.8 mg) and sulfoxonium ylide **2a** (0.15 mmol, 1.5 equiv., 25.2 mg) were added to a $CDCl_3$ (1 mL) solution of catalyst (*S*)-**A** (0.02 mmol, 0.20 equiv., 6.5 mg) and AcONa (0.02 mmol, 0.20 equiv., 1.6 mg). The resulting mixture was stirred at room temperature for 12 h, then treated directly with PCC (0.3 mmol, 3 equiv., 65 mg). Work-up and purification as above afforded compound **5aa** as a white solid in 35% yield (8.1 mg).

m.p. = 77-80 °C. $[\alpha]_D^{25}$ +7.2 (c = 0.25, $CH_2Cl_2$) for 96% ee. **IR** (ATR) ν(max) =1754 (s) 1722 (s) 1172 (s) 1076 (s) $cm^{-1}$. **$^1$H NMR** (400 MHz, $CDCl_3$, +25 °C) δ = 7.39 (dd, *J* = 7.6, 1.7 Hz, 1H), 7.30 – 7.22 (m, 1H), 7.13 (td, *J* = 7.5, 1.2 Hz, 1H), 7.05 – 6.98 (m, 1H), 4.26-4.21 (m, 2H), 3.04 (dd, *J* = 8.1, 4.2 Hz, 1H), 2.85 (dd, *J* = 8.1, 4.1 Hz, 1H), 2.07 (t, *J* = 4.1 Hz, 1H), 1.29 (t, *J* = 7.2 Hz, 3H). **$^{13}$C NMR** (101 MHz, $CDCl_3$, +25 °C) δ = 169.9, 163.9, 150.0, 128.7, 128.2, 124.7, 119.0, 117.4, 62.0, 27.5, 27.0, 25.2, 14.1. **HRMS** (ESI) m/z: $[M + Na]^+$ Calcd for $C_{13}H_{12}O_4Na$ 255.0628; Found 255.0633. **HPLC**: AD-H (*n*-hexane/*i*-PrOH 90:10, 0.75 mL/min) $t_{maj}$ = 11.4 min, $t_{min}$ = 15.7 min.

**Ethyl (1*S*,1a*S*,7b*R*)-1,1a,2,7b-tetrahydrocyclopropa[*c*]chromene-1-carboxylate 6aa**

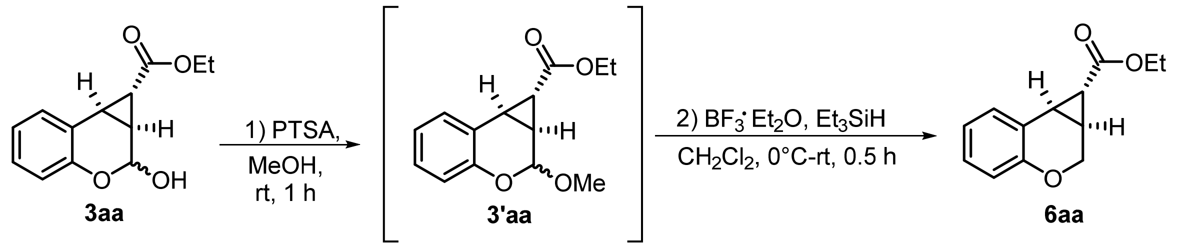


In a small vial equipped with a magnetic stirring bar, PTSA·$H_2O$ (0.1 mmol, 1 equiv., 95.1 mg) was added to a solution of cyclopropanchromanol **3aa** (0.1 mmol, 1 equiv., 23 mg) in 2.5 mL of MeOH. The reaction was stirred at rt for 1 h and then the desired intermediate **3'aa** was purified by a short plug on silica gel using DCM as eluent. Intermediate **3'aa** was then dissolved in 1 mL of DCM and cooled to 0 °C, then $BF_3$·$Et_2O$ (0.3 mmol, 3 equiv., 42.5 mg, 37 μL) and $Et_3SiH$ (0.3 mmol, 3 equiv., 34.9 mg, 48 μL) were added and the reaction was stirred at rt for 30 min. The mixture was then poured into a solution of $NaHCO_{3(sat)}$ and extracted with DCM (3x). The combined organic phases were dried over $MgSO_4$, filtered and evaporated under reduced pressure. The crude residue was purified by flash column chromatography on silica gel (DCM/*n*-hexane = 1:1) affording product **6aa**[8] as an oil in 67% yield (14.6 mg).

*One pot protocol:* In a small vial equipped with a magnetic stirring bar, aldehyde **1a** (0.1 mmol, 1 equiv., 14.8 mg) and sulfoxonium ylide **2a** (0.15 mmol, 1.5 equiv., 25.2 mg) were added to a $CDCl_3$ (1 mL) solution of catalyst (*S*)-**A** (0.02 mmol, 0.20 equiv., 6.5 mg) and AcONa (0.02 mmol, 0.20 equiv., 1.6 mg). The resulting mixture was stirred at room temperature for 12 h, then evaporated to dryness (replacing residual $CDCl_3$ with MeOH portions). The residue was dissolved in MeOH (1 mL), and treated with PTSA·$H_2O$ (0.1 mmol, 1 equiv., 95.1 mg). The reaction was stirred at rt for 1 h, then evaporated to dryness. The mixture containing intermediate **3'aa** was then dissolved in 1 mL of DCM and cooled to 0 °C, then $BF_3$·$Et_2O$ (0.3 mmol, 3 equiv., 42.5 mg, 37 μL) and $Et_3SiH$ (0.3 mmol, 3 equiv., 34.9 mg, 48 μL) were added and the reaction was stirred at rt for 30 min. Work-up and purification as above afforded compound **6aa** as an oil in 17% yield (3.7 mg). $[\alpha]_D^{25}$ -104.5 (c = 0.32, $CH_2Cl_2$) **IR** (ATR) ν(max) =1723 (m) 1253 (m) 1029 (s) $cm^{-1}$. **$^1$H NMR** (300 MHz, $CDCl_3$, +25 °C) δ = 7.24 (dd, *J* = 7.4, 1.6 Hz, 1H), 7.10 (ddd, *J* = 1.6, 7.5, 8.0 Hz, 1H), 6.91 (ddd, *J* = 1.2, 7.5, 8.0 Hz, 1H), 6.8 (dd, *J* = 1.0, 8.1 Hz, 1H), 4.37 (d, *J* = 11.1 Hz, 1H), 4.17 (q, *J* = 7.2 Hz, 2H), 3.91 (dd, *J* = 10.7, 0.8 Hz, 1H), 2.57 (dd, *J* = 4.4, 8.4 Hz, 1H), 2.37-2.28 (m, 2H) 1.27 (t, *J* = 7.2 Hz, 3H). **$^{13}$C NMR** (151 MHz, $CDCl_3$, +25 °C) δ = 172.2, 152.7, 128.7, 127.3, 124.0, 121.8, 117.3, 61.6,

[8] Racemic **6aa**: Ye, L.-W.; Sun, X.-L.; Li, C.-Y.; Tang, Y. *J. Org. Chem.* **2007**, *72*, 1335.

60.8, 26.9, 24.4, 22.7, 14.2. **HRMS** (ESI) m/z: $[M + Na]^+$ Calcd for $C_{13}H_{14}O_3Na$ 241.0835; Found 241.0841.

**Ethyl (1*S*,2*S*,3*R*)-2-(hydroxymethyl)-3-(2-hydroxyphenyl)cyclopropane-1-carboxylate 7aa**

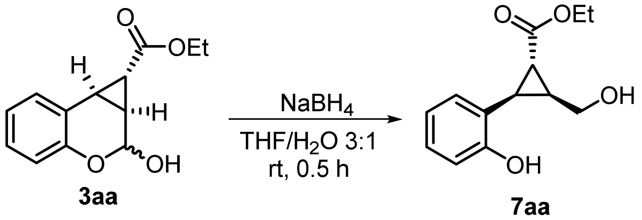


In a small vial equipped with a magnetic stirring bar $NaBH_4$ (0.225 mmol, 1.5 equiv., 8.5 mg) was added to a cooled (0 °C) solution of cyclopropanchromanol **3aa** (0.15 mmol, 35.1 mg) in a 3:1 THF/$H_2O$ mixture (1.5 mL). After 30 minutes stirring at 0 °C, the mixture was poured into a solution of $NH_4Cl_{(sat)}$ and extracted with DCM. The combined organic phases were dried over $MgSO_4$, filtered and evaporated under reduced pressure. The crude residue was purified by flash column chromatography on silica gel (*n*-hexane/acetone = 5:1) affording product **7aa** as a yellow oil in 91% yield (32.5 mg).

*One pot protocol:* In a small vial equipped with a magnetic stirring bar, aldehyde **1a** (0.1 mmol, 1 equiv., 14.8 mg) and sulfoxonium ylide **2a** (0.15 mmol, 1.5 equiv., 25.2 mg) were added to a $CDCl_3$ (1 mL) solution of catalyst (*S*)-**A** (0.02 mmol, 0.20 equiv., 6.5 mg) and AcONa (0.02 mmol, 0.20 equiv., 1.6 mg). The resulting mixture was stirred at room temperature for 12 h, then evaporated to dryness (replacing residual $CDCl_3$ with THF portions). The residue was dissolved in a 3:1 THF/$H_2O$ mixture (1.5 mL), cooled to 0 °C, and treated with $NaBH_4$ (0.225 mmol, 1.5 equiv., 8.5 mg). After 1.5 h, additional $NaBH_4$ (0.225 mmol, 1.5 equiv., 8.5 mg) was added. The mixture was stirred at 0 °C for an additional 30 minutes. Work-up and purification as described above afforded compound **7aa** as a yellow oil in 50% yield (11.8 mg).

$[\alpha]_D^{25}$ -18.0 (c = 0.5, $CH_2Cl_2$). **IR** (ATR) ν(max) =3452 (w, br) 3165 (w, br) 1701 (s) 1219 (s) 1188 (s) 1018 (s) $cm^{-1}$. **$^1$H NMR** (400 MHz, $CDCl_3$, +25 °C) δ = 7.20-7.15 (m, 2H), 6.90-6.85 (m, 2H), 4.22 (q, *J* = 7.2 Hz, 2H), 3.90 (dd, *J* = 11.2, 4.3 Hz, 1H), 2.85 (t, *J* = 10.6 Hz, 1H), 2.7 (dd, *J* = 5.0, 9.1 Hz, 1H), 2.25-2.20 (m, 1H), 1.91 (t, *J* = 4.9 Hz, 1H), 1.30 (t, *J* = 7.1 Hz, 3H). **$^{13}$C NMR** (101 MHz, $CDCl_3$, +25 °C) δ = 173.1, 154.9, 131.3, 128.8, 122.8, 121.1, 116.7, 61.5, 61.1, 29.1, 26.6, 22.9, 14.2. **HRMS** (ESI) m/z: $[M + Na]^+$ Calcd for $C_{13}H_{16}O_4Na$ 259.0941; Found 259.0946.

**Ethyl (1*S*,1a*S*,2*R*,7b*R*)-2-(2-ethoxy-2-oxoethyl)-1,1a,2,7b-tetrahydrocyclopropa[*c*]chromene-1-carboxylate and Ethyl (1*S*,1a*S*,2*S*,7b*R*)-2-(2-ethoxy-2-oxoethyl)-1,1a,2,7b-tetrahydro cyclopropa[*c*]chromene-1-carboxylate 8aa**

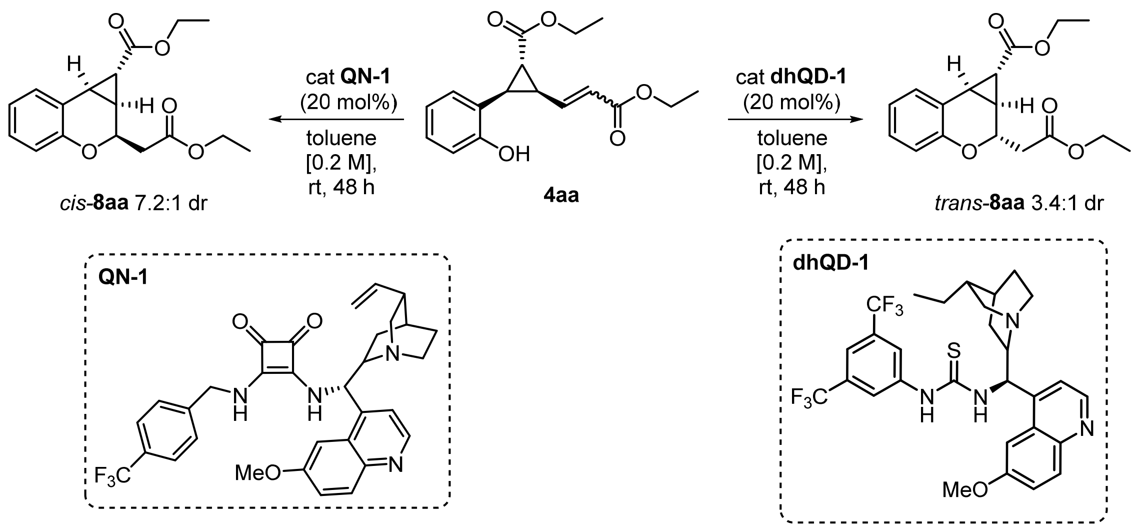


***cis*-8aa-selective reaction (QN-1 catalyzed):**

In a small vial equipped with a magnetic stirring bar catalyst **QN-1** (0.02 mmol, 0.2 equiv., 11.3 mg) was added to a solution of **4aa** (0.1 mmol, 1 equiv., 30.4 mg) in toluene (0.5 mL). The reaction was stirred 48 h at rt, then the catalyst was removed by a short plug of silica gel using $Et_2O$ as eluent. After evaporation of the solvents, the residue was analyzed by $^1$H NMR spectroscopy indicating a 7.2:1 diastereomeric ratio favoring the *cis*-**8aa** isomer. Subsequently, the crude residue was purified by flash column chromatography on silica gel (*n*-hexane/AcOEt = 14:1) affording product **8aa** as a diastereomeric mixture in 58% yield (17.6 mg) and 99% ee for the *cis*-**8aa** isomer. *Cis*/*trans*-**8aa** (*cis*-enriched): **IR** (ATR) ν(max) =1720 (s) 1263 (s) 1172 (s) cm$^{-1}$. $^{\mathbf{1}}$**H NMR** (600 MHz, $CDCl_3$, +25 °C) [signals of the *cis*-isomer] δ = 7.25 (dd, *J* = 7.6, 1.7 Hz, 1H), 7.10 (td, *J* = 7.7, 1.6 Hz, 1H), 6.92 (td, *J* = 7.4, 1.0 Hz, 1H), 6.78 (br d, *J* = 8.1 Hz, 1H), 4.39 (br t, *J* = 6.5 Hz, 1H), 4.26-4.19 (m, 2H), 4.19-4.12 (m, 2H), 2.83 (dd, *J* = 15.5, 7.5 Hz, 1H), 2.75 (dd, *J* = 15.5, 5.5 Hz, 1H), 2.61 (dd, *J* = 9.2, 3.5 Hz, 1H), 2.38 (ddd, *J* = 9.2, 4.4, 1.4 Hz, 1H), 2.25 (br t, *J* = 4.0 Hz, 1H), 1.30 (t, *J* = 7.0 Hz, 3H), 1.27 (t, *J* = 7.0 Hz, 3H). $^{\mathbf{13}}$**C NMR** (151 MHz, $CDCl_3$, +25 °C) [signals of the *cis*-isomer] δ = 171.9, 170.2, 152.5, 128.4, 127.4, 123.6, 122.0, 117.4, 68.0, 60.9, 60.8, 40.4, 30.6, 23.7, 23.1, 14.23, 14.21. **HRMS** (ESI) m/z: [M + H]$^+$ Calcd for $C_{17}H_{21}O_5$ 305.1389; Found 305.1389. **HPLC**: IC (*n*-hexane/*i*-PrOH 95:5, 0.75 mL/min) *cis*-**8aa** isomer: $t_{min}$ = 18.5 min, $t_{maj}$ = 30.8 min.

***trans*-8aa-selective reaction (dhQD-1 catalyzed):**

In a small vial equipped with a magnetic stirring bar catalyst **dhQD-1** (0.02 mmol, 0.2 equiv., 12.0 mg) was added to a solution of **4aa** (0.1 mmol, 1 equiv., 30.4 mg) in toluene (0.5 mL). The reaction

was stirred 48 h at rt, then the catalyst was removed by a short plug of silica gel using $Et_2O$ as eluent. After evaporation of the solvents, the residue was analyzed by $^1$H NMR spectroscopy indicating a 3.4:1 diastereomeric ratio favoring the *trans*-**8aa** isomer. Subsequently the crude residue was purified by column chromatography on silica gel (*n*-hexane/AcOEt = 14:1) affording product **8aa** as a diastereomeric mixture in 58% yield (17.5 mg) and 99% ee for the *trans*-**8aa** isomer. *Cis*/*trans*-**8aa** (*trans*-enriched): **IR** (ATR) ν(max) =1726 (s) 1704 (s) 1261 (s) 1186 (s) 1029 (s) cm$^{-1}$. **$^1$H NMR** (600 MHz, $CDCl_3$, +25 °C) [signals of the *trans*-isomer] δ = 7.22 (dd, *J* = 7.5, 1.5 Hz, 1H), 7.11 (td, *J* = 7.8, 1.6 Hz, 1H), 6.92 (td, *J* = 7.4, 1.0 Hz, 1H), 6.75 (br d, *J* = 8.1 Hz, 1H), 4.91 (br t, *J* = 6.9 Hz, 1H), 4.19-4.13 (m, 4H), 2.59 (d, *J* = 15.4, 7.9 Hz, 1H), 2.56 (dd, *J* = 8.8, 5.2 Hz, 1H), 2.49 (dd, *J* = 15.4, 5.8 Hz, 1H), 2.28 (br t, *J* = 4.0 Hz, 1H), 2.27-2.24 (m, 1H), 1.27 (t, *J* = 7.3 Hz, 3H), 1.26 (t, *J* = 7.3 Hz, 3H). **$^{13}$C NMR** (151 MHz, $CDCl_3$, +25 °C) [signals of the *trans*-isomer] δ = 171.9, 170.3, 149.5, 128.5, 127.8, 123.7, 122.1, 118.4, 67.6, 60.9, 60.8, 38.4, 29.6, 25.5, 22.2, 14.23, 14.18. **HPLC**: IC (*n*-hexane/*i*-PrOH 95:5, 0.75 mL/min) *trans*-**8aa** isomer: $t_{min}$ = 23.2 min $t_{maj}$ = 34.1 min.

## Determination of the relative configuration of compounds 4’ab and 3ab

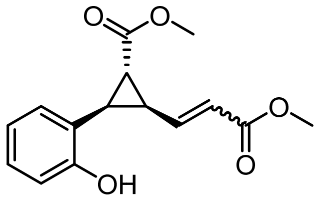

**4'ab**

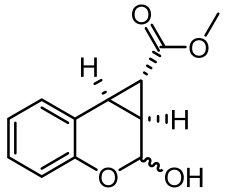

**3ab**

Compound **4’ab** was selected for the assignment of the relative disposition of cyclopropane protons. Full assignment of $^1$H NMR signals was preliminarily determined by J-coupling and HSQC and HMBC bidimensional sequences. The $^1$H NMR spectrum shows that the $H_3$ signal is coupled with the $H_1$, $H_2$ and $H_{1E}$ giving a ddd signal. The J constant $H_3$-$H_{1E}$ ($J$ = 9.5 Hz) is easily confirmed by the $H_{1E}$ signal at 6.22 ppm. The large value of J coupling constant with the cyclopropane $H_2$ ($J$ = 9.7 Hz) and the smaller J constant with the last cyclopropane $H_1$ ($J$ = 4.3 Hz) suggests that $H_3$ is cis and trans, respectively, to these protons. To confirm the 1R*,2R*,3S* relative configuration, NOESY-1D spectra were acquired (Figure S1).

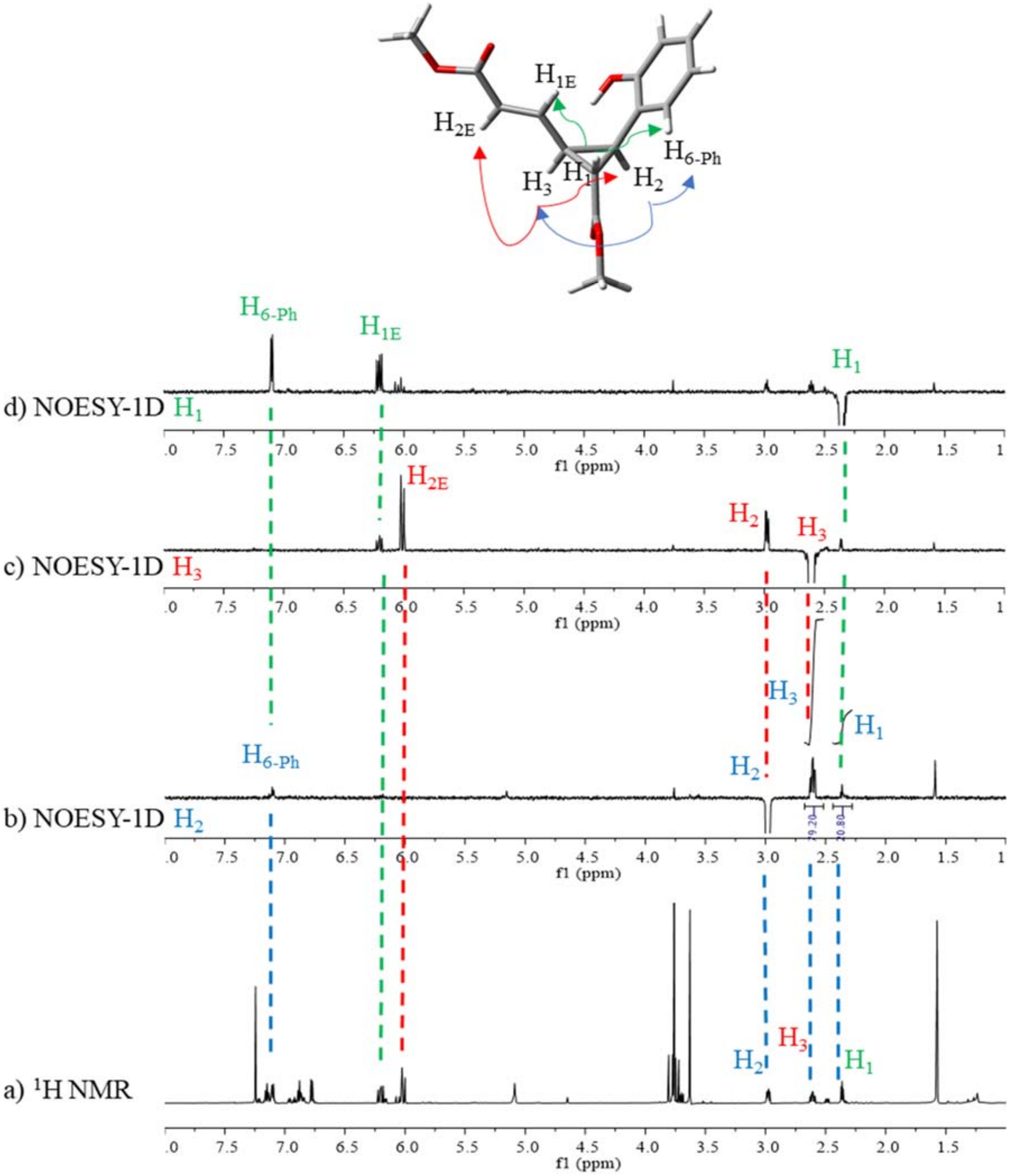


**Figure S1.** DPFGSE-NOE spectra of **4'ab** (600 MHz in $CDCl_3$, T = 25 °C); a) control $^1$H-NMR spectrum; b) saturation of cyclopropane $H_2$ signal; c) saturation of cyclopropane $H_3$ signal; d) saturation of cyclopropane $H_1$ signal.

On saturation of the proton in position 2 of the cyclopropane ($H_2$), strong NOE effect is generated on the $H_3$ hydrogen (79.2%), smaller on the $H_1$ (20.8%) and $H_{6\text{-Ph}}$ signals (Fig. S1 trace b). If the protons were in the same side, a 50% of NOE effect should occur. When $H_3$ is saturated only $H_2$ and $H_{2E}$ give strong NOE effect. Finally, on saturation of the $H_1$, strong NOE effect is generated on the $H_{1E}$ and $H_{6\text{-Ph}}$ signals confirming that the substituents of the cyclopropane are in the same side of $H_1$.

These results indicate that cyclopropane has a 1R*,2R*,3S* relative configuration.

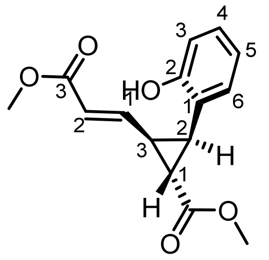


methyl (1*R*,2*R*,3*S*)-2-(2-hydroxyphenyl)-3-((*E*)-3-methoxy-3-oxoprop-1-en-1-yl)cyclopropane-1-carboxylate

Having in hand the relative configuration of compound **4'ab**, the relative assignment of the cyclopropyl ring in both major and minor products of compound **3ab** was done. The two diastereoisomers differ for the configuration of the hemiacetal carbon 2. Keeping in mind the C.I.P. priority groups, the relative configuration is 1S*,1aS*,2*,7bR*. To assign the relative configuration of the two isomers, NOESY-1D experiments were acquired (Figure S2).

On saturation of the aromatic proton $H_7$, NOE effect is generated on the $H_{7b}$ cyclopropane proton (Figure 2, trace b). When hemiacetal proton of major diastereosiomer ($H_{2maj}$) is saturated, both $H_1$ and $H_{1a}$ give strong NOE effect (Figure S2, trace c), suggesting its disposition in the same side (3D structure in Figure S2, major). Vice versa, $H_1$ does not give NOE effect on saturation of hemiacetal proton of minor diastereosiomer ($H_{2min}$) (trace d) confirming their opposite side (3D structure in Figure S2, minor).

In conclusion, the relative configuration of the major diastereoisomer of **3ab** is 1S*,1aS*,2R*,7bR* and the minor diastereoisomer of **3ab** is 1S*,1aS*,2S*,7bR*.

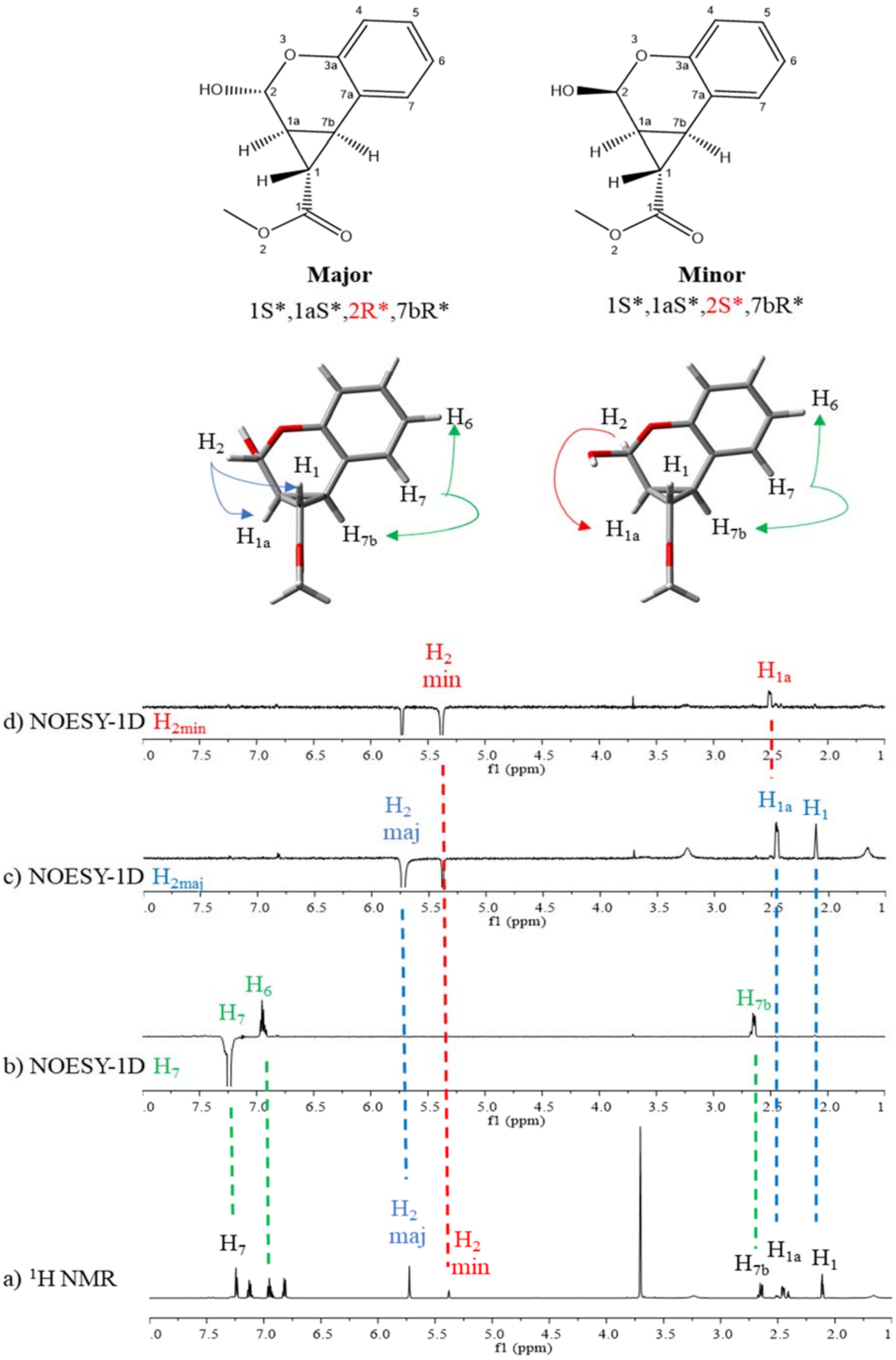


**Figure S2.** DPFGSE-NOE spectra of **3ab** (600 MHz in $CDCl_3$, T = 25 °C); a) control $^1$H-NMR spectrum; b) saturation of $H_7$ aromatic signal; c) saturation of $H_{2maj}$ major signal of hemiacetal; d) saturation of $H_{2min}$ minor signal of hemiacetal.

## Determination of the relative configuration of compounds *cis*-8aa and *trans*-8aa

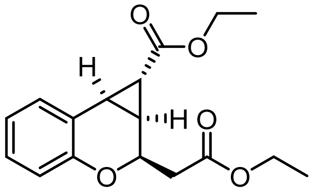


1a,2-*cis*-**8aa**

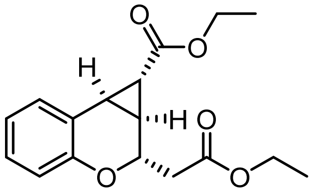


1a,2-*trans*-**8aa**

To determine the relative configuration between the C1a and C2 chirality centers of compounds *cis*- and *trans*-**8aa**, NOESY-1D experiments were performed on a mixture enriched in the *trans*-**8aa** isomer (Figure S3). Irradiation of the aromatic signal corresponding to $H_7$ at 7.22 ppm (dd, $J$ = 7.5, 1.5 Hz, 1H) gave a NOE effect on the signal at 6.92 ppm (td, $J$ = 7.4, 1.0 Hz, 1H), assigned to $H_6$, and on the signal at 2.56 ppm (dd, $J$ = 8.8, 5.2 Hz, 1H) (Figure S3, trace b). The latter signal could thus be assigned to $H_{7b}$. Such assignment was confirmed by irradiating the signal at 6.75 ppm (br d, $J$ = 8.1 Hz, 1H, $H_4$), which gave NOE effect only on the aromatic signal at 7.11 ppm (td, $J$ = 7.8, 1.6 Hz, 1H, $H_5$) (not shown). Irradiating the signal at 4.91 ppm (br t, $J$ = 6.9 Hz, 1H), assigned to $H_2$ based on its chemical shift, gave NOE effect on the two cyclopropanic proton signals at 2.28 ppm (br t, J = 4.0 Hz, 1H), and 2.27-2.24 ppm (m, 1H) (Figure S3, trace c). Irrespective of the assignment of these signals to $H_1$ and $H_{1a}$, this result indicates a *cis*-relationship between $H_2$ and $H_1$ and thus, ultimately, a 1a,2-*trans* relationship.

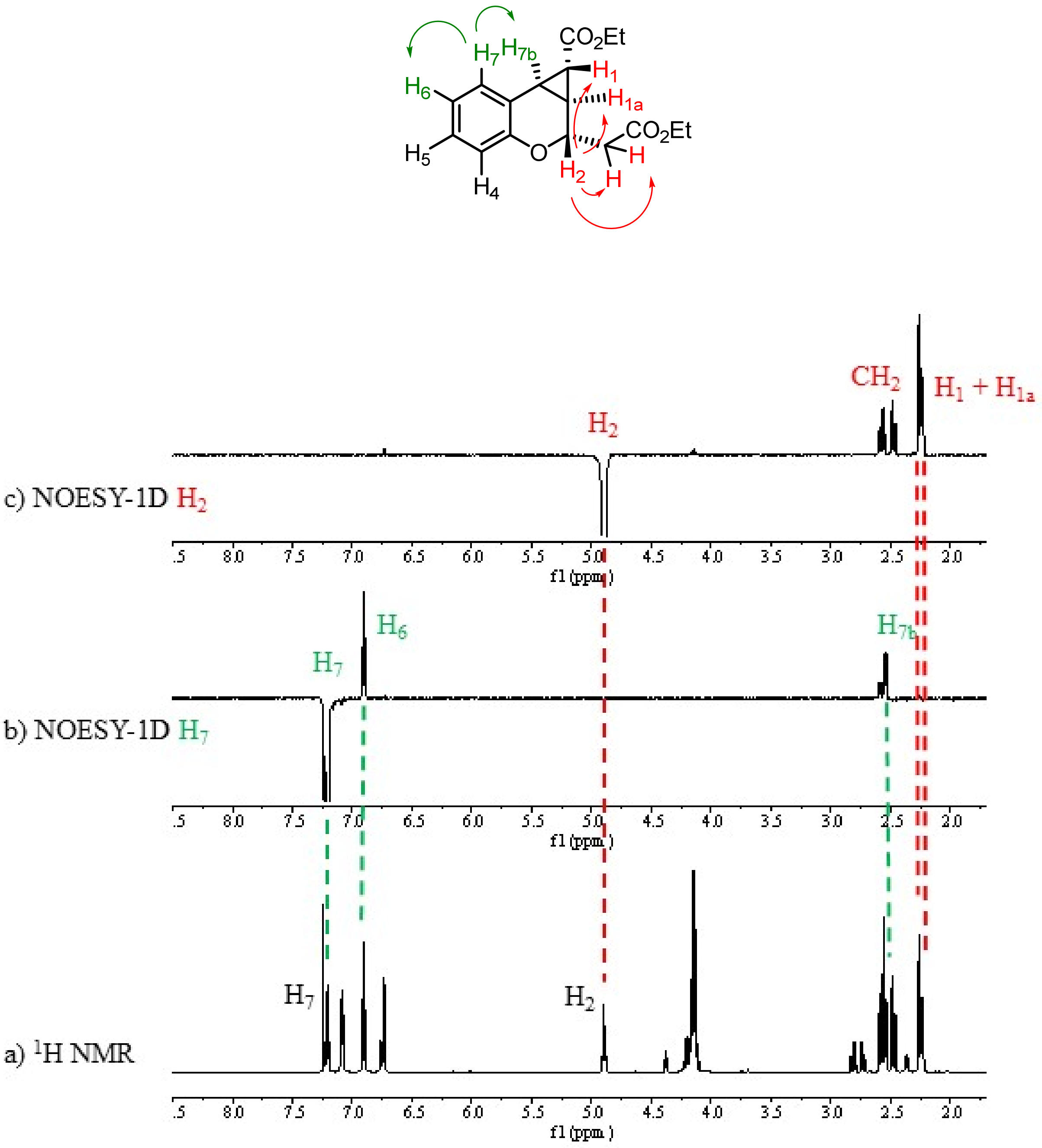


**Figure S3.** DPFGSE-NOE spectra of **8aa**, predominantly 1a,2-*trans* (600 MHz in $CDCl_3$, T = 25 °C); a) control $^1$H-NMR spectrum; b) saturation of $H_7$ aromatic signal; c) saturation of $H_2$.

## Determination of the absolute configuration of compounds 4’ab and 3ab

The determination of the absolute configuration (AC) of these products using X-Ray diffractometer was unfeasible because good crystals were not obtained. Therefore, the electronic circular dichroism (ECD) method was selected for the absolute configuration assignment.

**Absolute Configuration of Compound 4’ab**

The experimental ECD spectrum of compound **4’ab** was acquired in the 195-400 nm region using a JASCO J-810 spectropolarimeter in HPLC-grade acetonitrile solution. Concentration was about $2 \cdot 10^{-4}$ M, optimized in order to have a maximum absorbance less than 1, with a cell path of 0.1 cm. The spectrum was obtained by the average of 6 scans at 50 nm·min$^{-1}$ scan rate.

The ECD spectrum for compound **4’ab** shows a large negative band at 280 nm and a positive one at 220 nm (vide infra).

For compound **4’ab**, two ground state geometries, within less than 1 kcal/mol, were found and optimized at the B3LYP/6-31G(d,p) level of theory (Figure S4), and validated by frequency analysis (no imaginary frequency was observed). The two geometries differ in the dihedral angle of the *o*-phenol, which can be -145.4° (73.3%) or +58.7° (26.7%).

The ECD spectra have been calculated in the gas phase for the two conformations with 1*R*,2*R*,2*S* absolute configuration using TD-DFT. Four different hybrid functionals (BH&HLYP[9] and M06-2X,[10] ωB97-XD[11] and CAM-B3LYP[12]) and the basis set (6-311++G(2d,p) were employed (Figure S4).

---

[9] In Gaussian 16 the BH&HLYP functional has the form: 0.5*$^{EXHF}$ + 0.5*$^{EXLSDA}$ + 0.5*$\Delta EX^{Becke88}$ + $EC^{LYP}$

[10] Zhao, Y.; Truhlar, D. G. *Theor. Chem. Acc.* **2008**, *120*, 215.

[11] Chai, J.-D.; Head-Gordon, M. *Phys. Chem. Chem. Phys.* **2008**, *10*, 6615. Iikura, H.; Tsuneda, T.; Yanai, T.; Hirao, K. *J. Chem. Phys.* **2001**, *115*, 3540.

[12] Yanai, T.; Tew, D.; Handy, N. *Chem. Phys. Lett.* **2004**, *393*, 51.

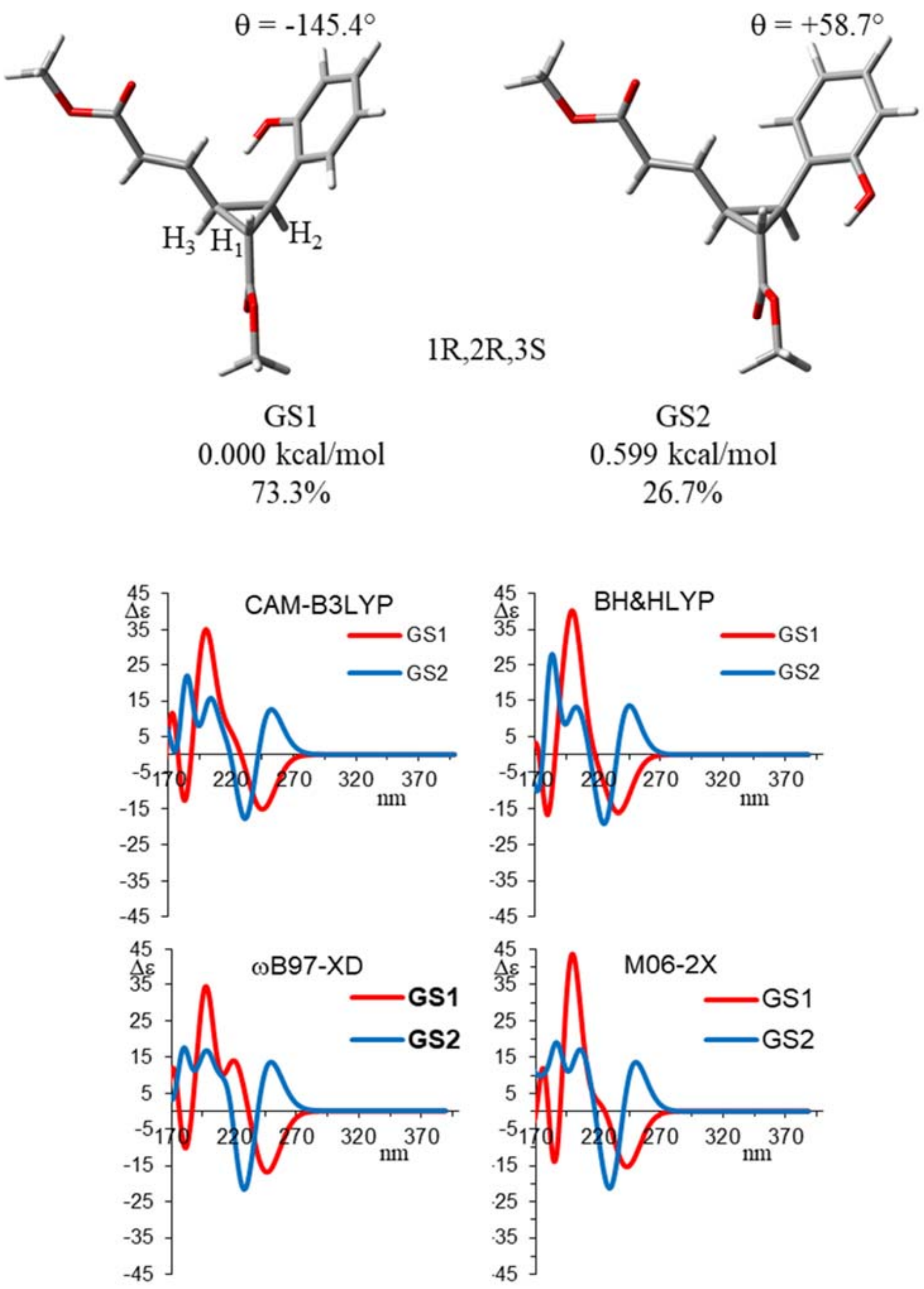


**Figure S4**. Top: Ground state geometries of **4’ab** with 1*R*,2*R*,3*S* absolute configuration. Bottom: calculated ECD spectra.

The calculated spectra for the two geometries are quite different (Figure S4), therefore the weighted sum was done and compared with the experimental ECD spectrum (Figure S5). The simulated spectra were vertically scaled and red-shifted to get the best match with the experimental spectrum. A very good overlap with the experimental ECD spectrum permits to assign the 1*R*,2*R*,2*S* absolute configuration to compound **4’ab**.

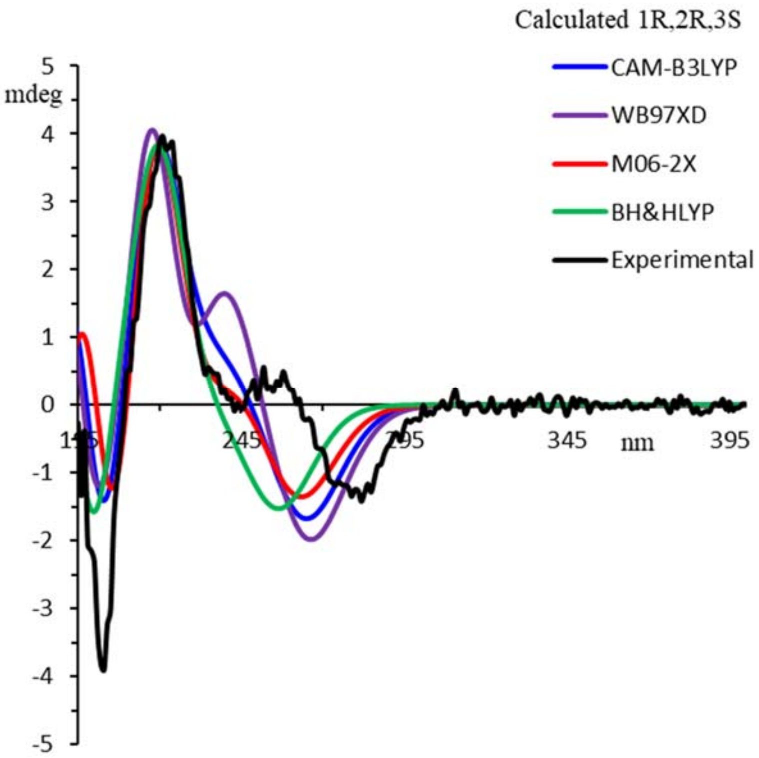


**Figure S5** Overlap of calculated and experimental (black line) ECD spectra for compound (1*R*,2*R*,2*S*)-**4'ab**.

### Absolute Configuration of Compound 3ab

For compound **3ab**, two diastereomeric geometries were found. Starting from relative configuration, 1*S*,1a*S*,2*R*,7b*R* geometry was calculated for the major diastereoisomer (78% by NMR) and 1*S*,1a*S*,2*S*,7b*R* geometry was calculated for the minor diastereoisomer (22% by NMR).

The ECD spectra have been calculated in the gas phase using TD-DFT, such as for compound **4'ab**. Both calculated spectra for the two diastereoisomers have a good overlap with the experimental ECD of the mixture, meaning that the hemiacetal chiral carbon does not influence the biggest band at 230 nm, that is mainly due to the chromophores tetrahydrocyclopropa[*c*]chromene (Figures S6 and S7). However, the weighted sum of the two diastereoisomers was done and compared with the experimental ECD spectrum (Figure S8). The simulated spectra were vertically scaled and red-shifted to get the best match with the experimental spectrum. A very good overlap with the experimental ECD spectrum permits to assign the 1*S*,1a*S*,2*R*,7b*R* A.C. to the major diastereosiomer and 1*S*,1a*S*,2*S*,7b*R* A.C. to the minor diastereoisomer, thus confirming the correctness of the assignment previously done on **4'ab**.

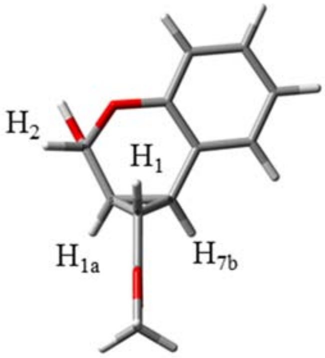


**Major**

1S,1aS,2R,7bR

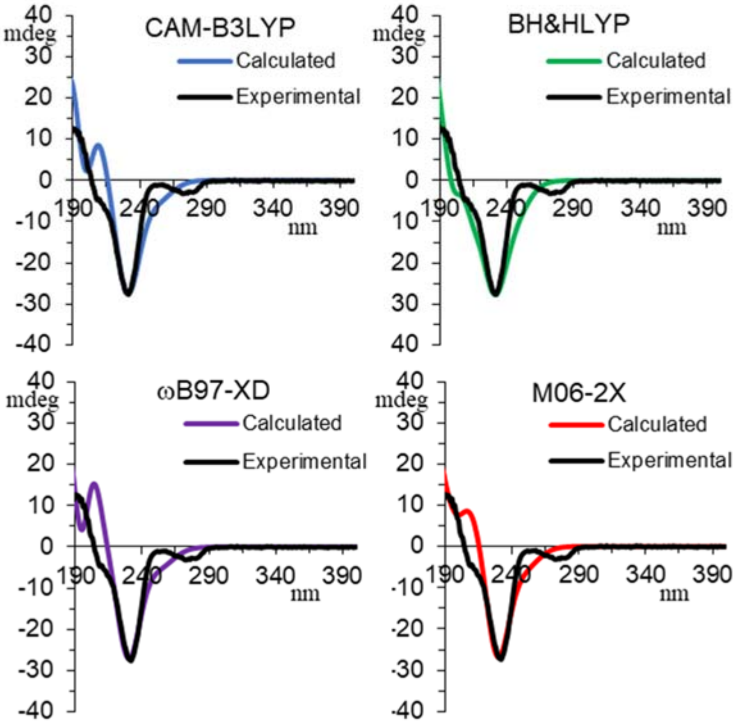


**Figure S6**. Overlap of calculated ECD spectra for the major diastereoisomer of **3ab**, and the experimental ECD of the diastereomeric mixture.

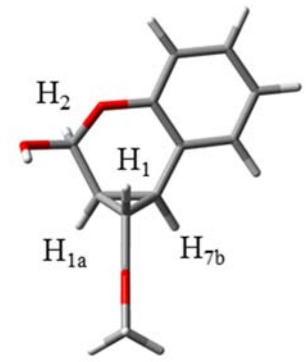


**Minor**

1S,1aS,2S,7bR

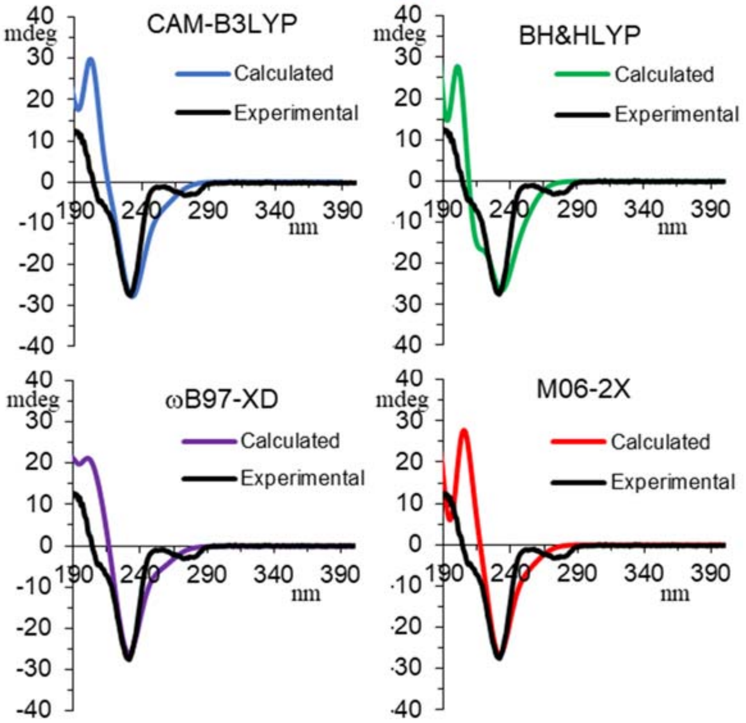


**Figure S7**. Overlap of calculated ECD spectra for the minor diastereoisomer of **3ab**, and the experimental ECD of the diastereomeric mixture.

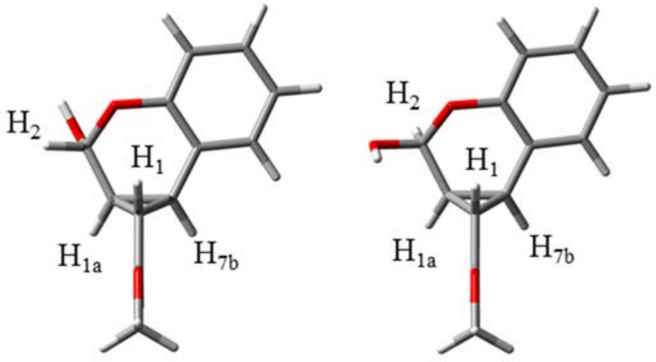


**Major**
1S,1aS,2R,7bR
78%

**Minor**
1S,1aS,2S,7bR
22%

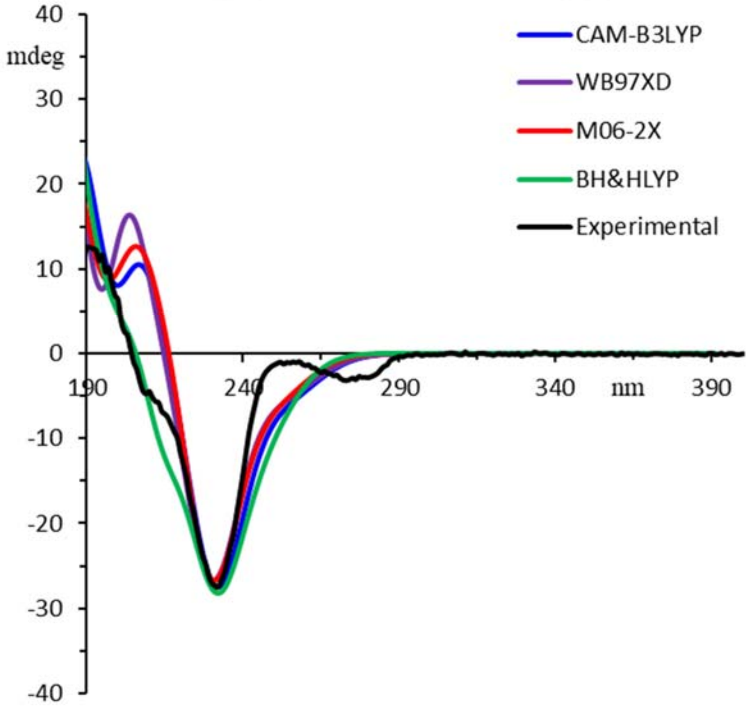


**Figure S8** Overlap of calculated, the weighted sum of the two diastereoisomers of **3ab**, and experimental (black line) ECD spectra.

# Copies of $^{1}$H and $^{13}$C NMR spectra of products 4-8

**Ethyl (1R,2S,3R)-2-(3-ethoxy-3-oxoprop-1-en-1-yl)-3-(2-hydroxyphenyl)cyclopropane-1-carboxylate 4aa**

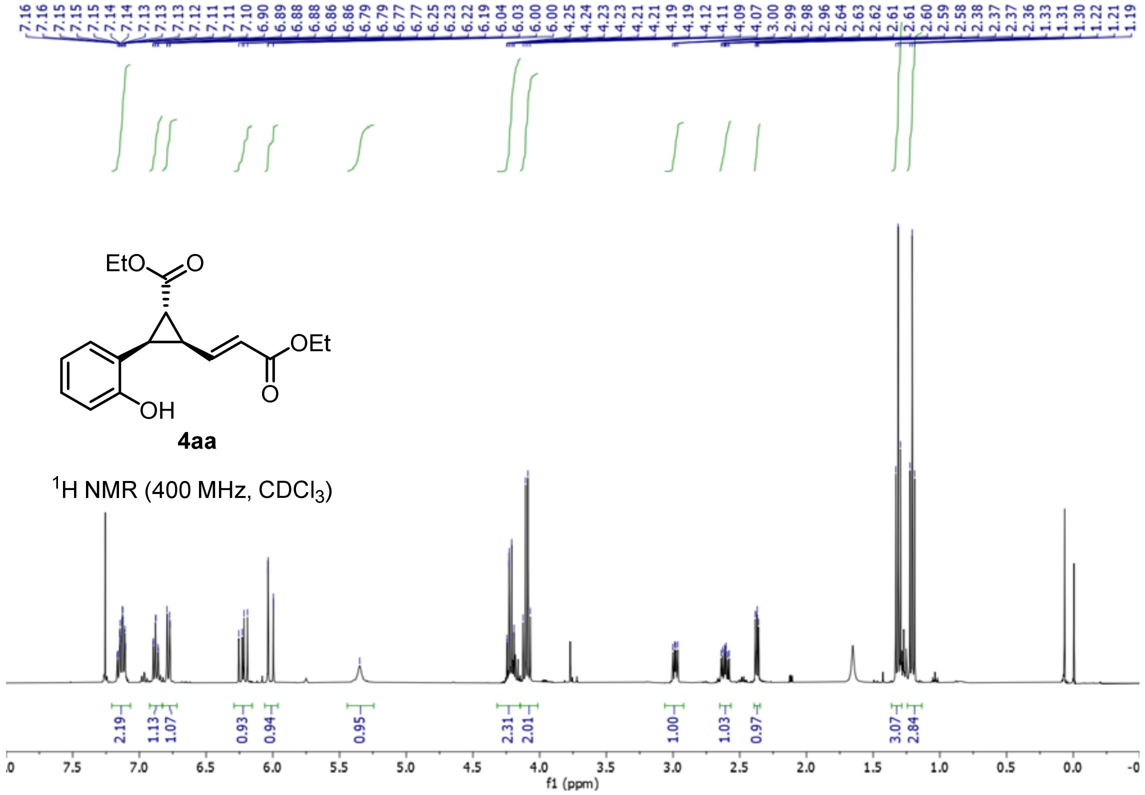


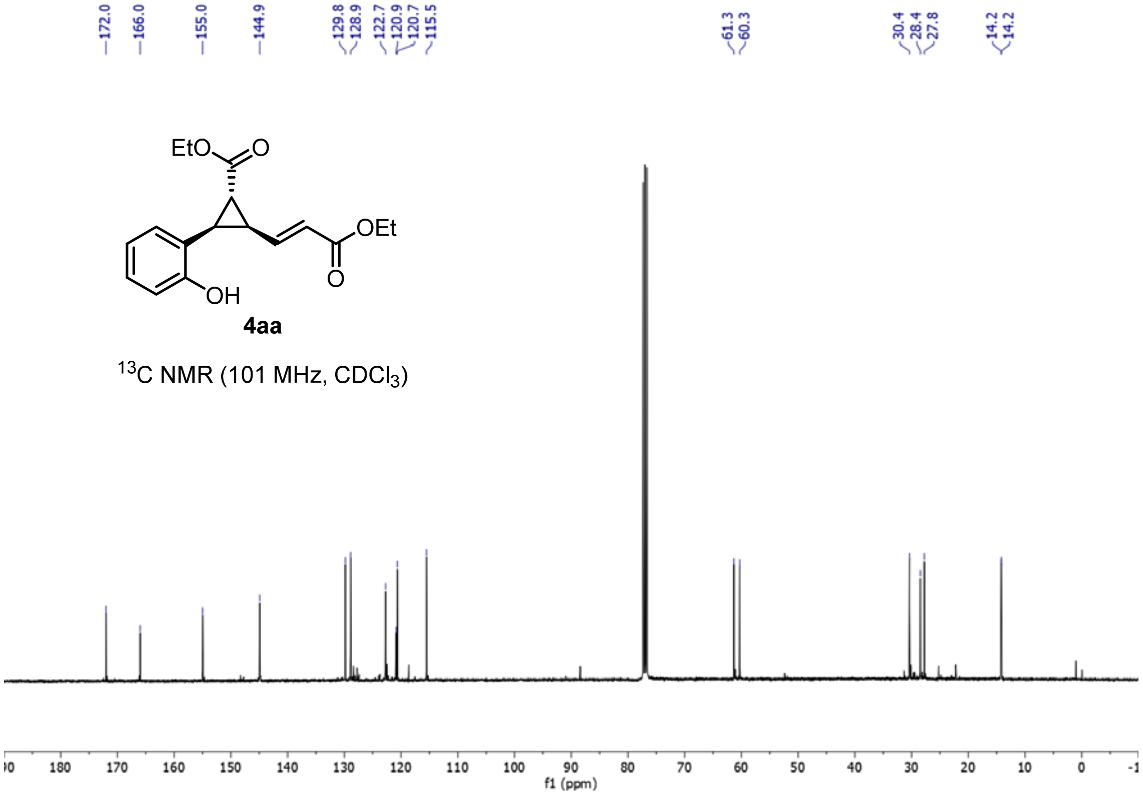

**Methyl (1R,2S,3R)-2-(3-ethoxy-3-oxoprop-1-en-1-yl)-3-(2-hydroxyphenyl)cyclopropane-1-carboxylate 4ab**

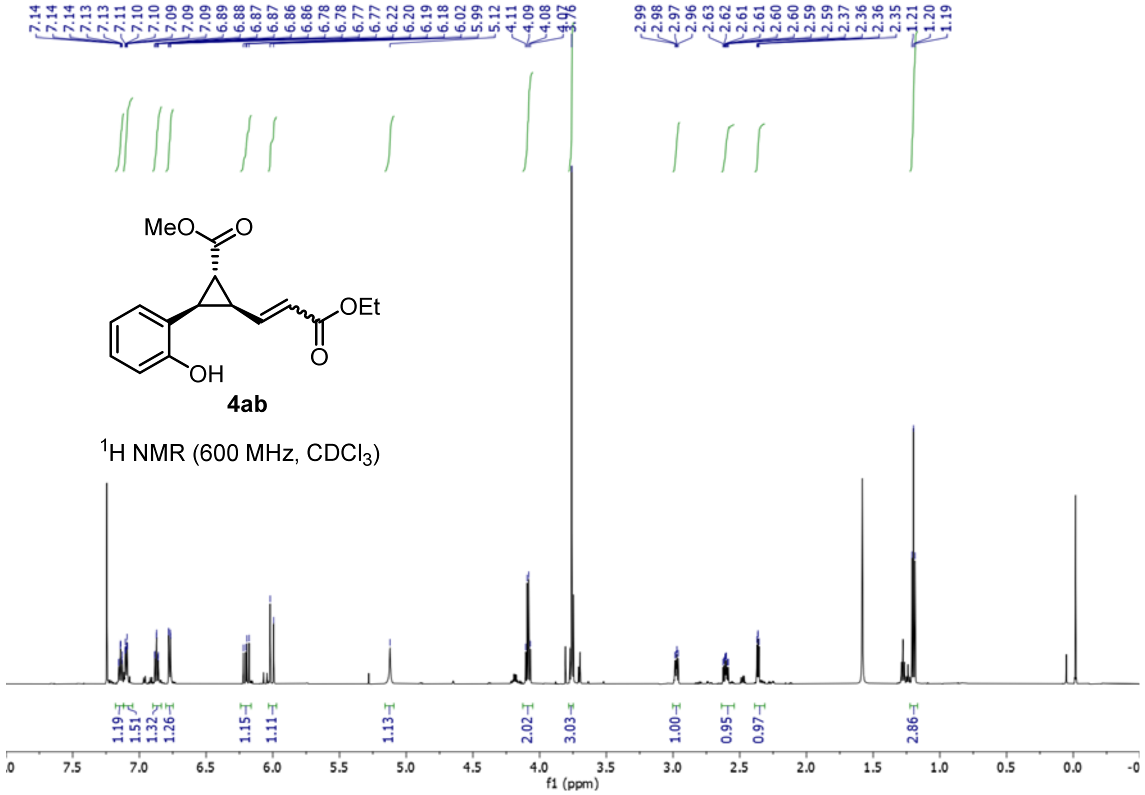


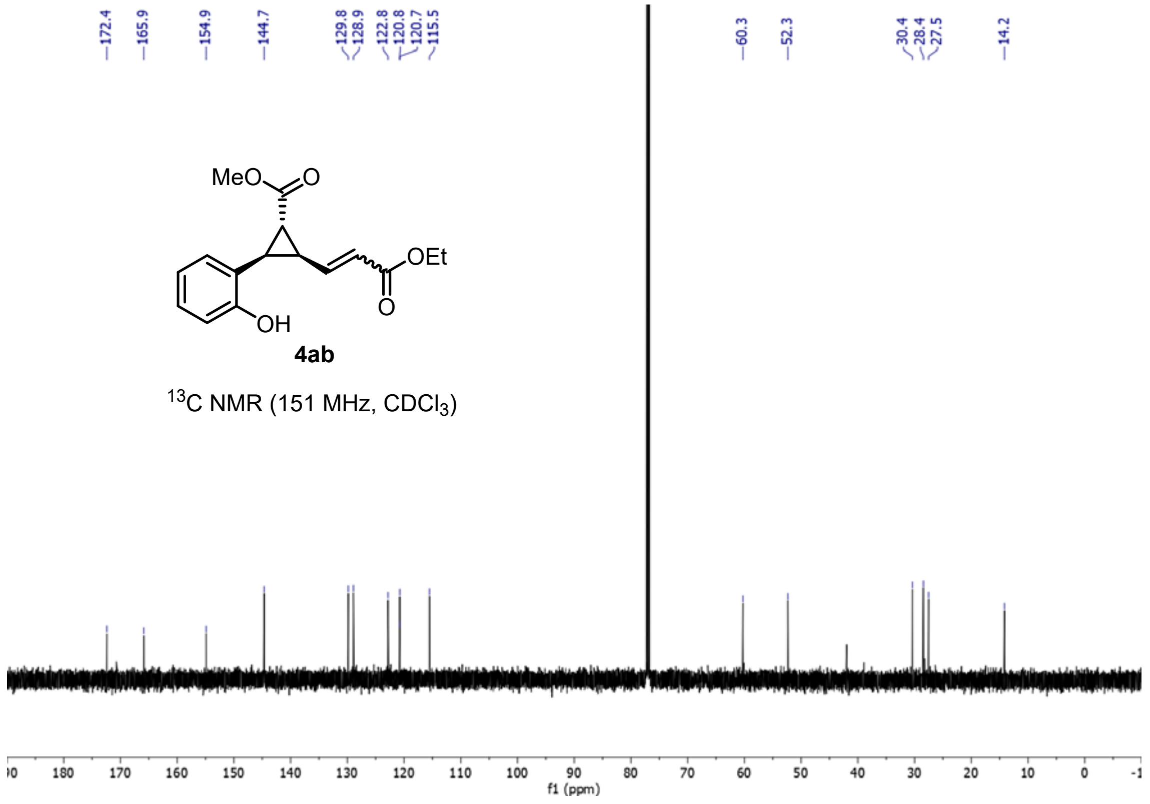

**Methyl (1R,2R,3S)-2-(2-hydroxyphenyl)-3-((E)-3-methoxy-3-oxoprop-1-en-1-yl)cyclopropane-1-carboxylate 4’ab**

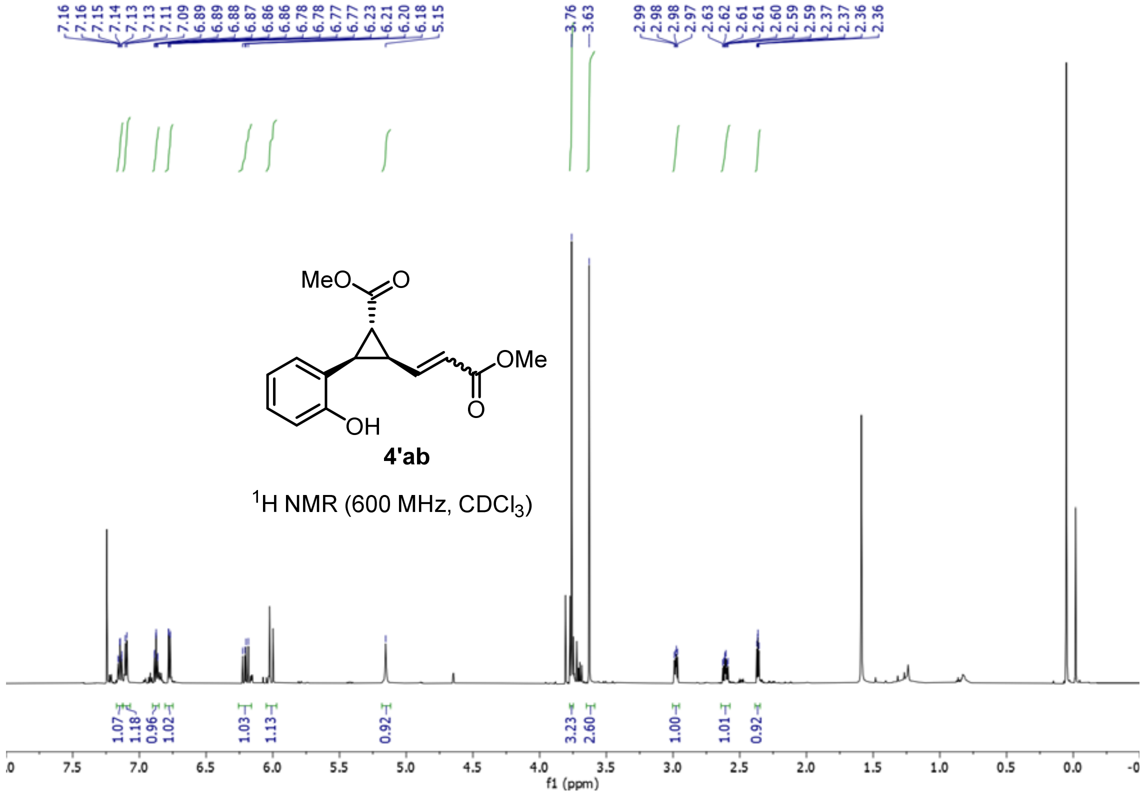


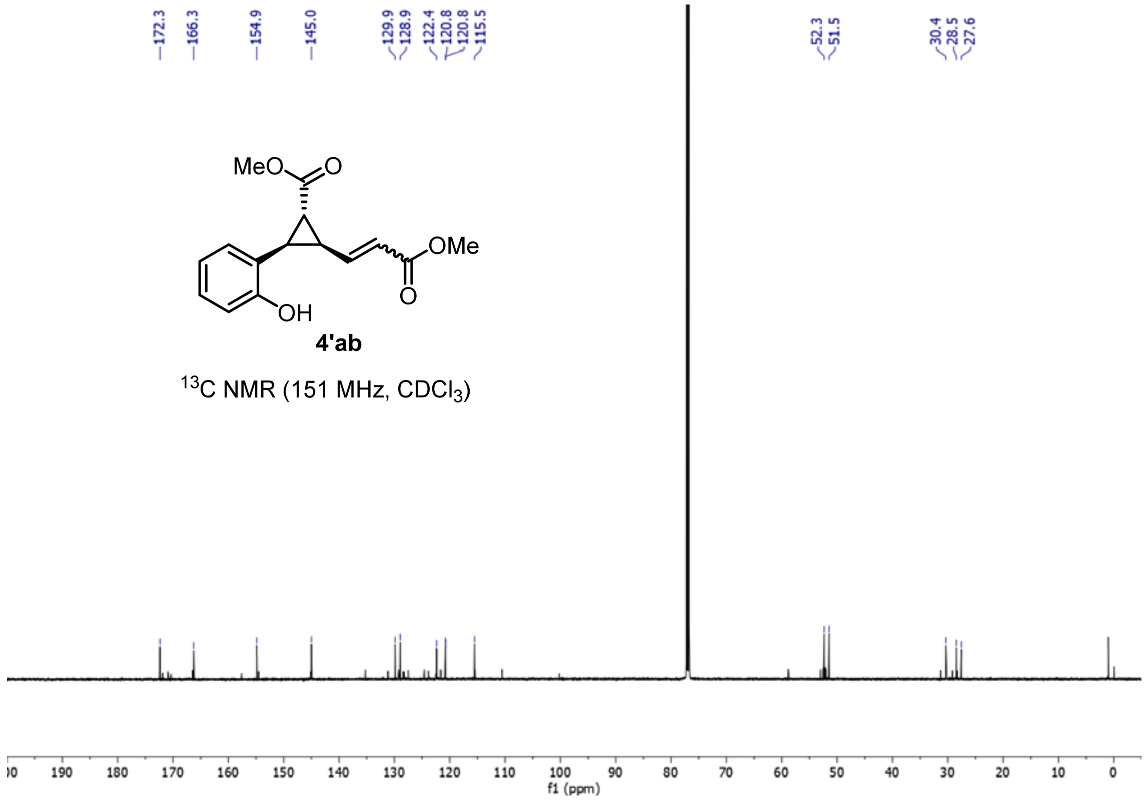

**Butyl (1R,2S,3R)-2-(3-ethoxy-3-oxoprop-1-en-1-yl)-3-(2-hydroxyphenyl)cyclopropane-1-carboxylatecarboxylate 4ac**

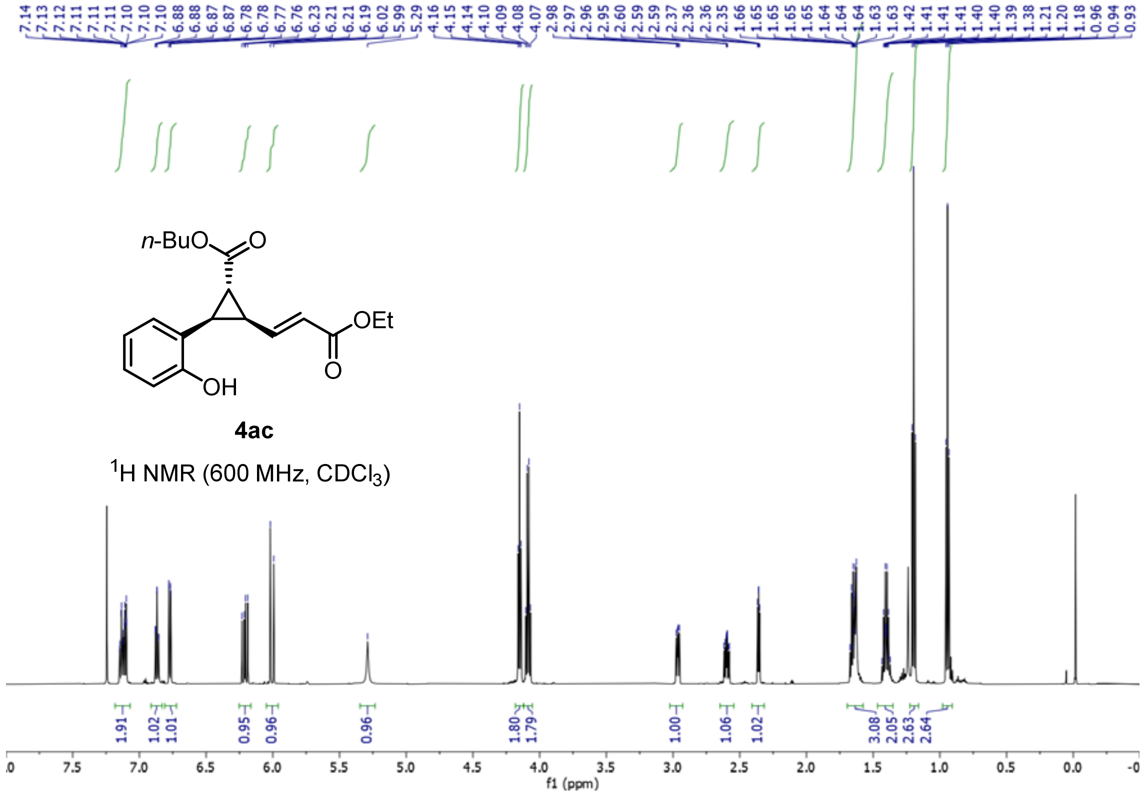


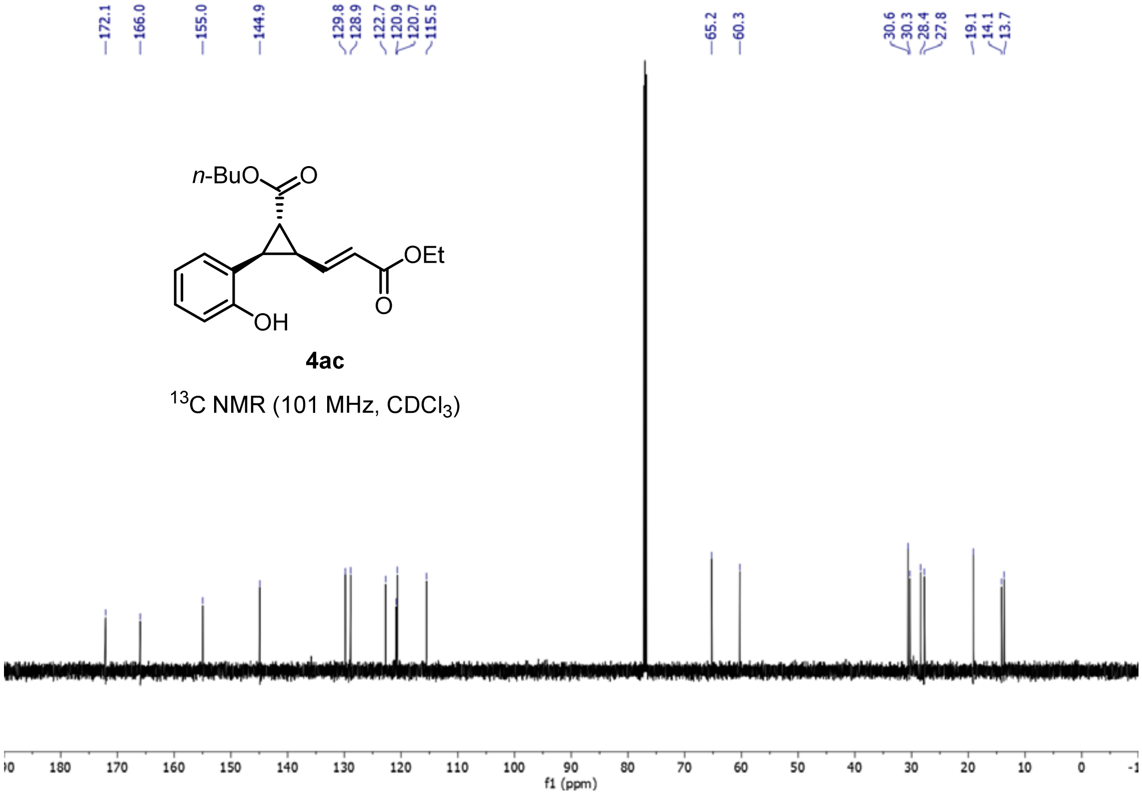

**Isobutyl (1R,2S,3R)-2-(-3-ethoxy-3-oxoprop-1-en-1-yl)-3-(2-hydroxyphenyl)cyclopropane-1-carboxylate 4ad**

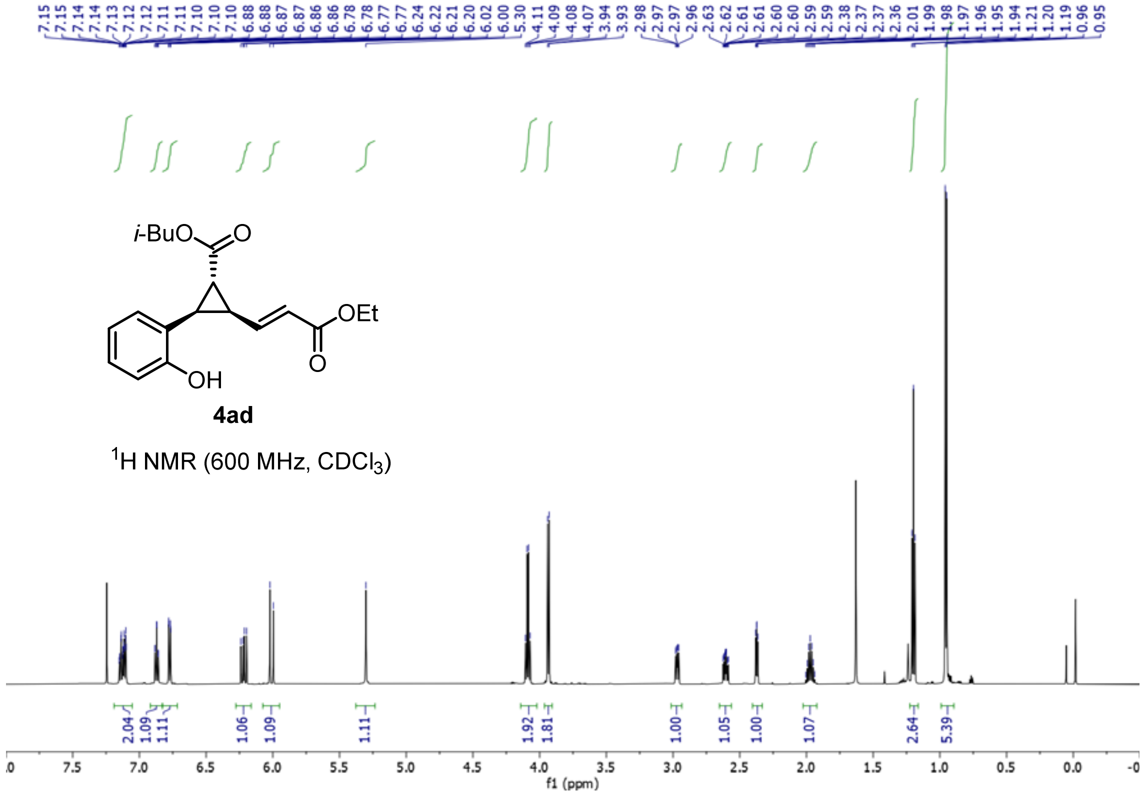


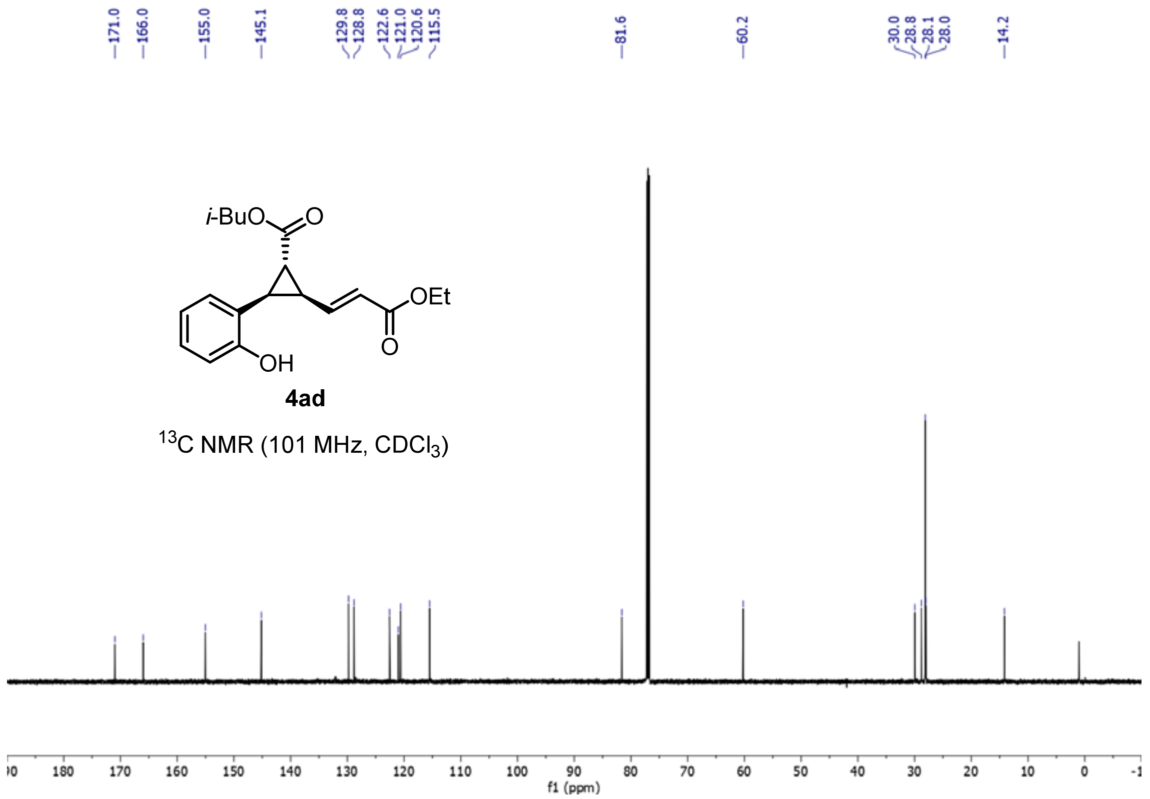

**Tert-butyl (1R,2S,3R)-2-(-3-ethoxy-3-oxoprop-1-en-1-yl)-3-(2-hydroxyphenyl)cyclopropane-1-carboxylate 4ae**

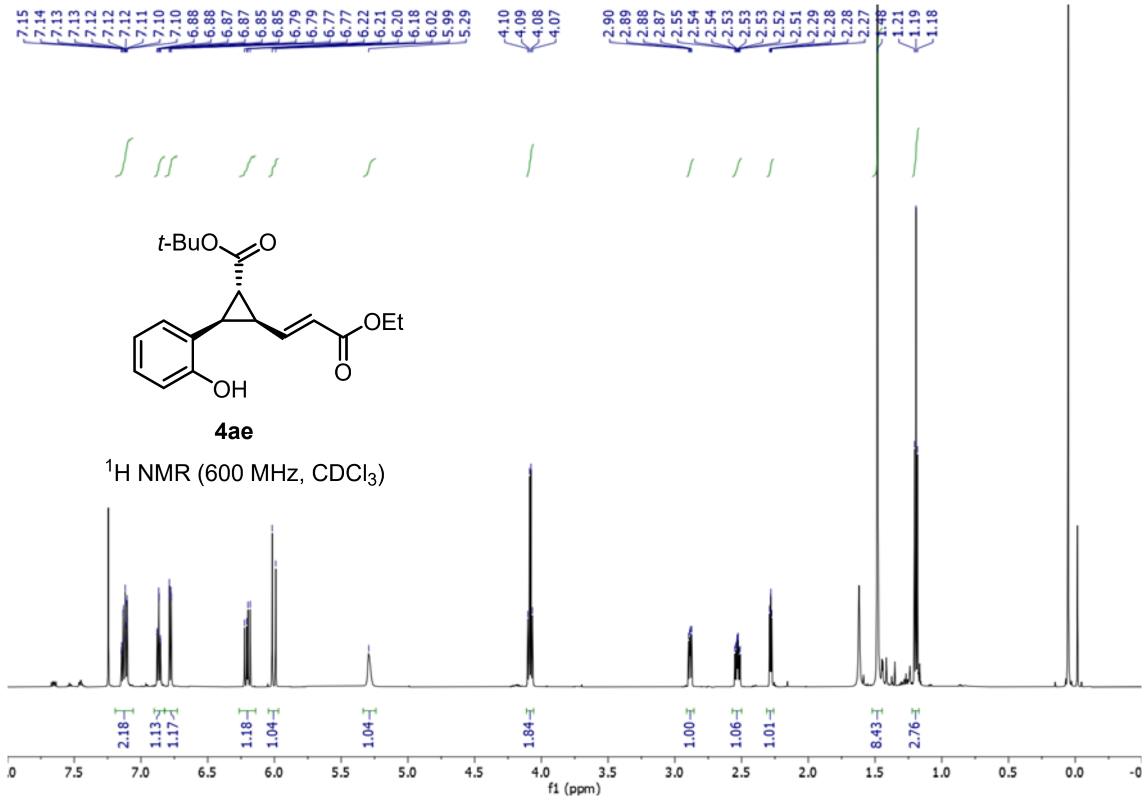


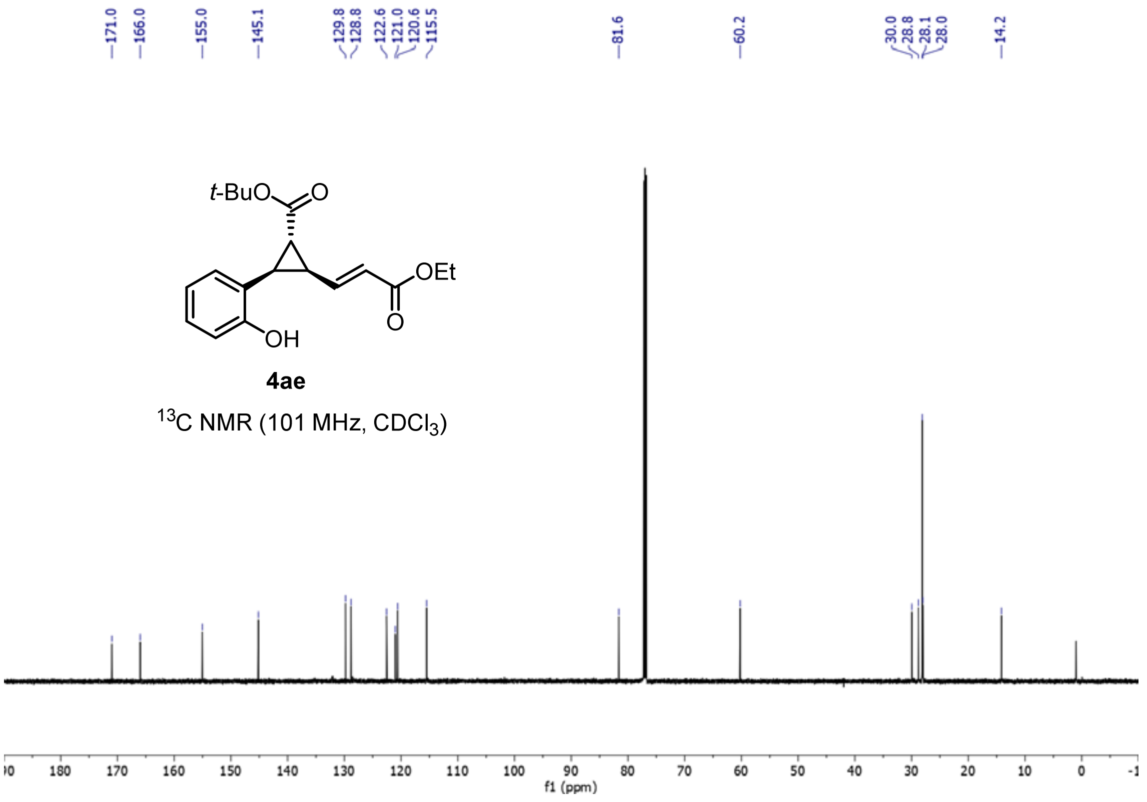

**Allyl (1R,2S,3R)-2-(3-ethoxy-3-oxoprop-1-en-1-yl)-3-(2-hydroxyphenyl)cyclopropane-1-carboxylate 4af**

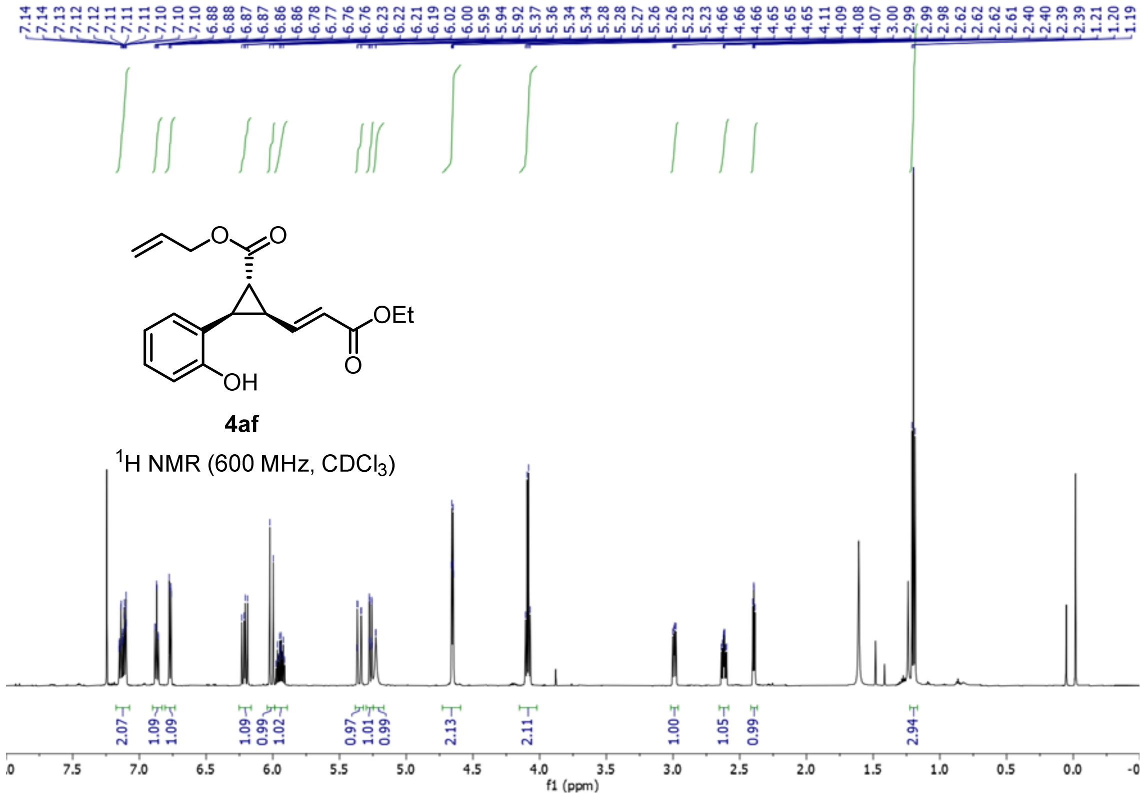


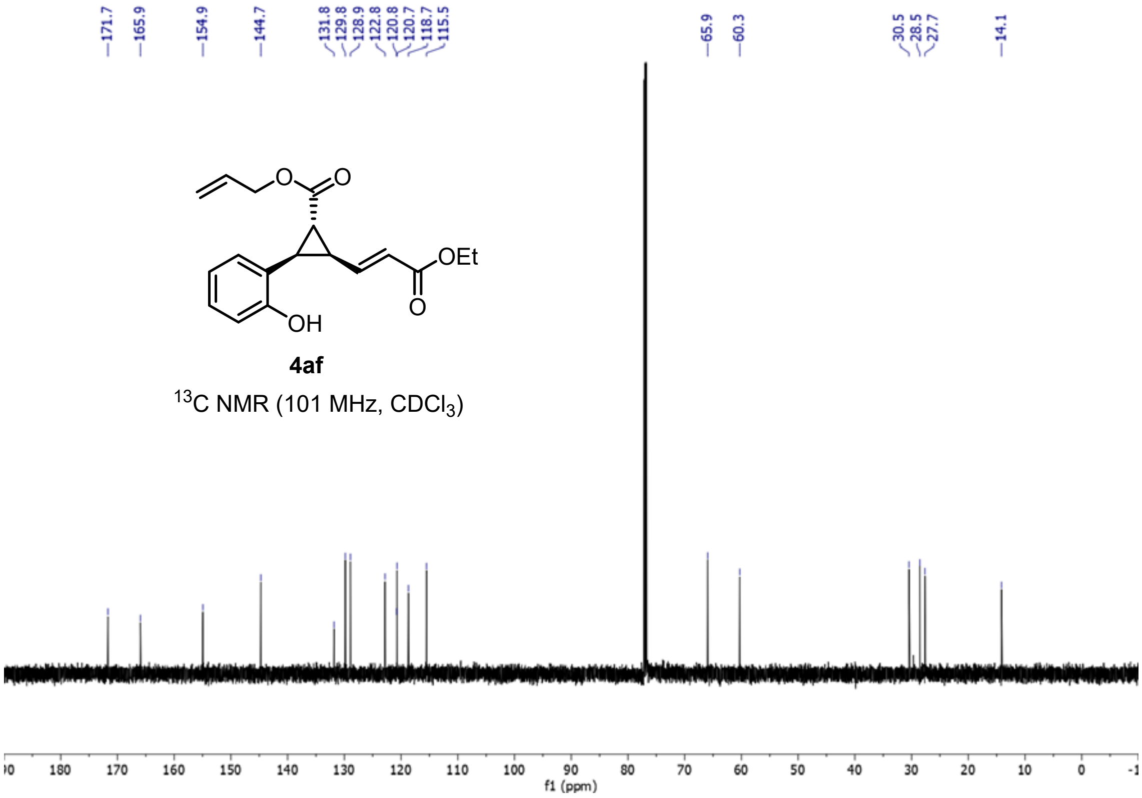

**Benzyl (1R,2S,3R)-2-(3-ethoxy-3-oxoprop-1-en-1-yl)-3-(2-hydroxyphenyl)cyclopropane-1-carboxylate 4ag**

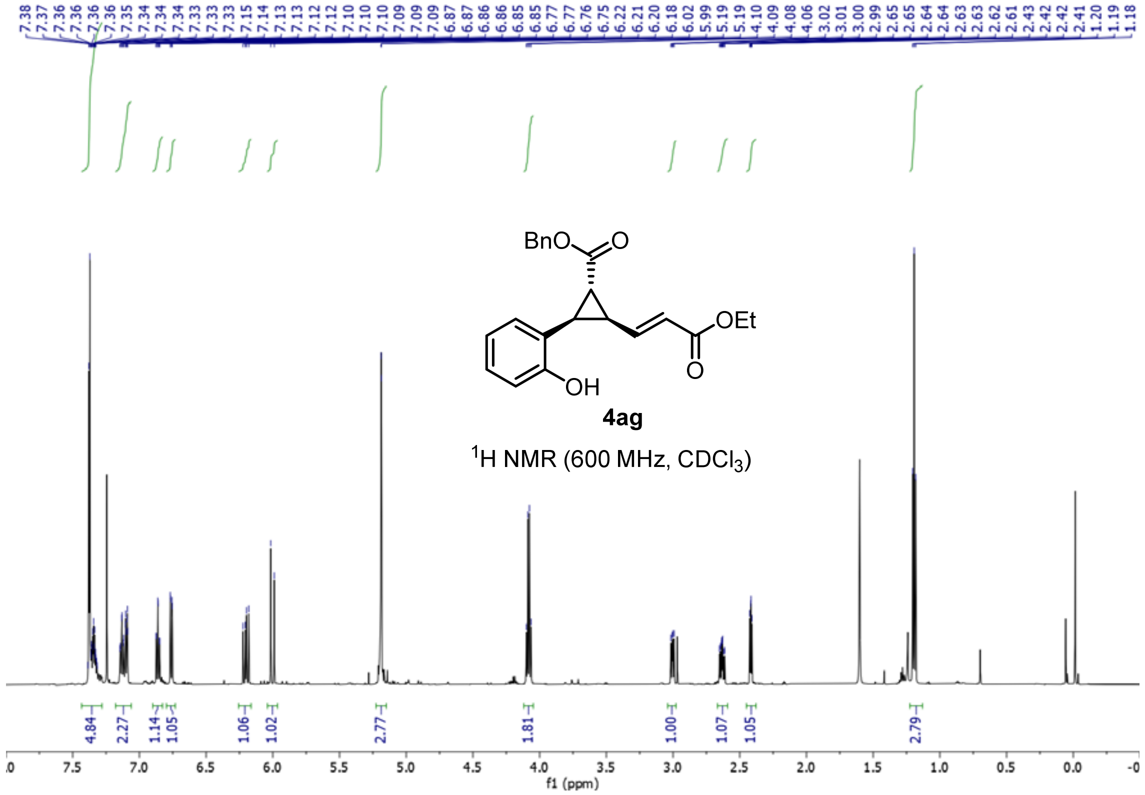


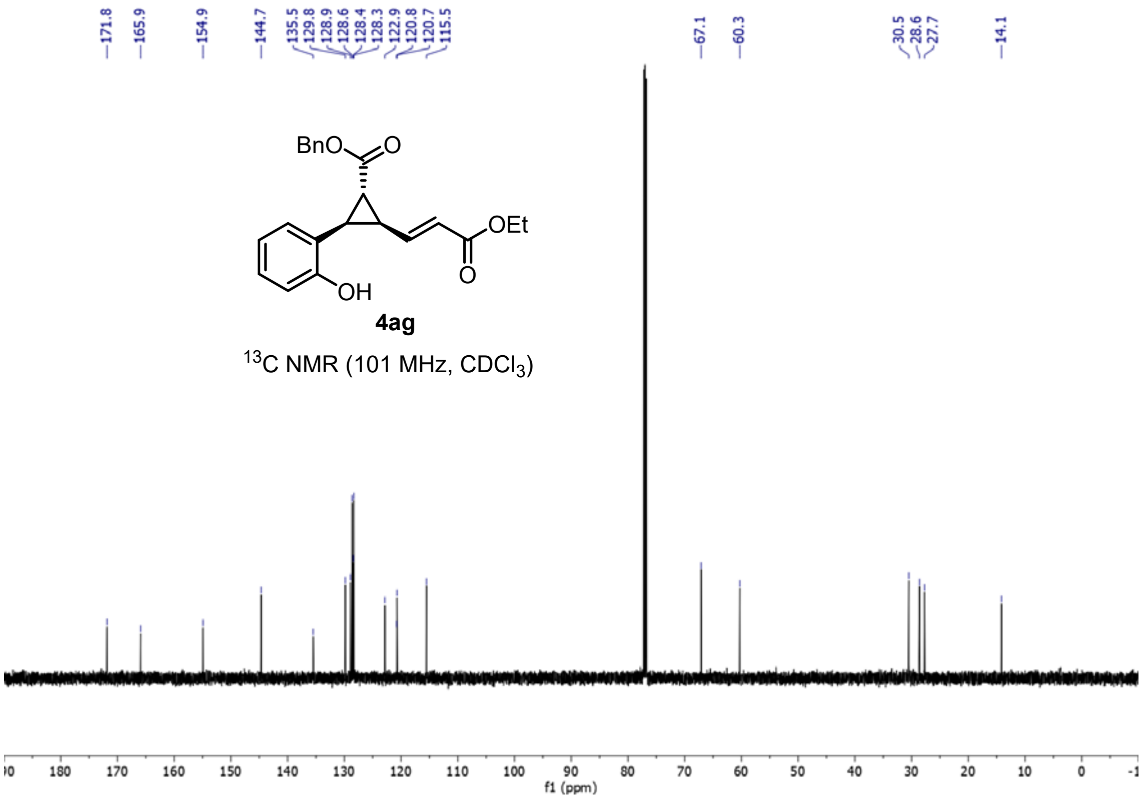

**Ethyl--3-((1S,2R,3R)-2-benzoyl-3-(2-hydroxyphenyl)cyclopropyl)acrylate 4ah**

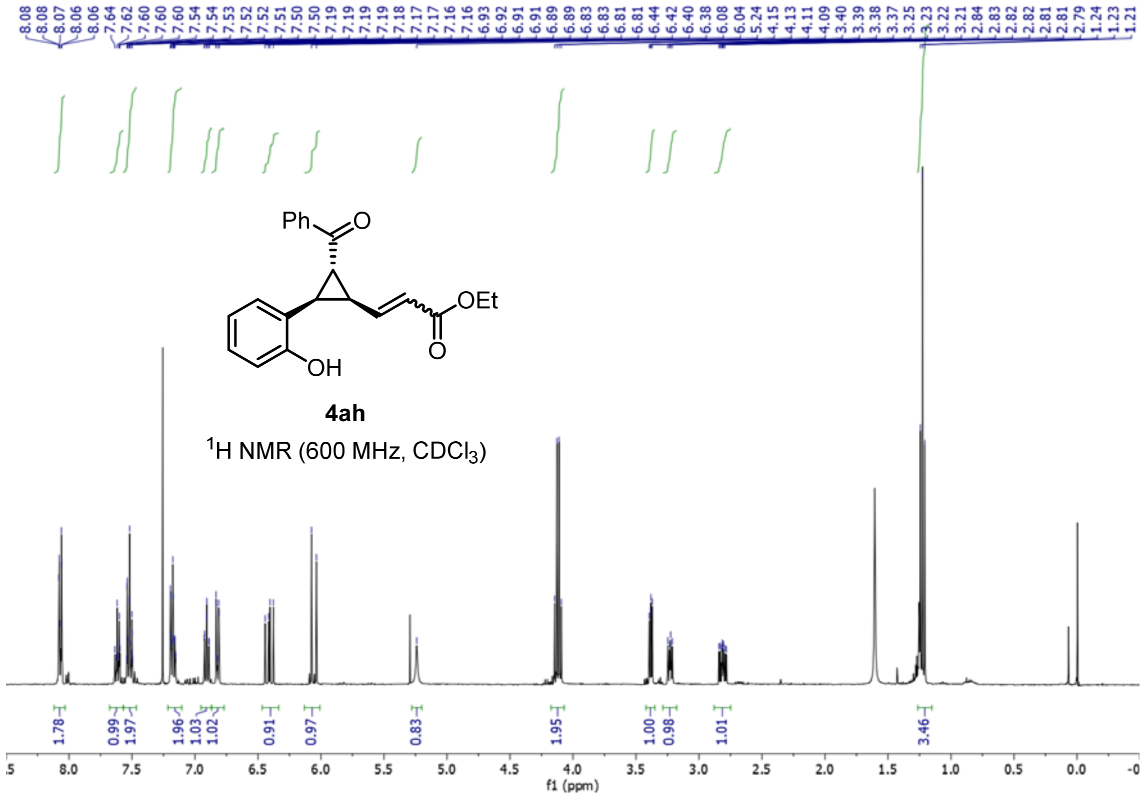


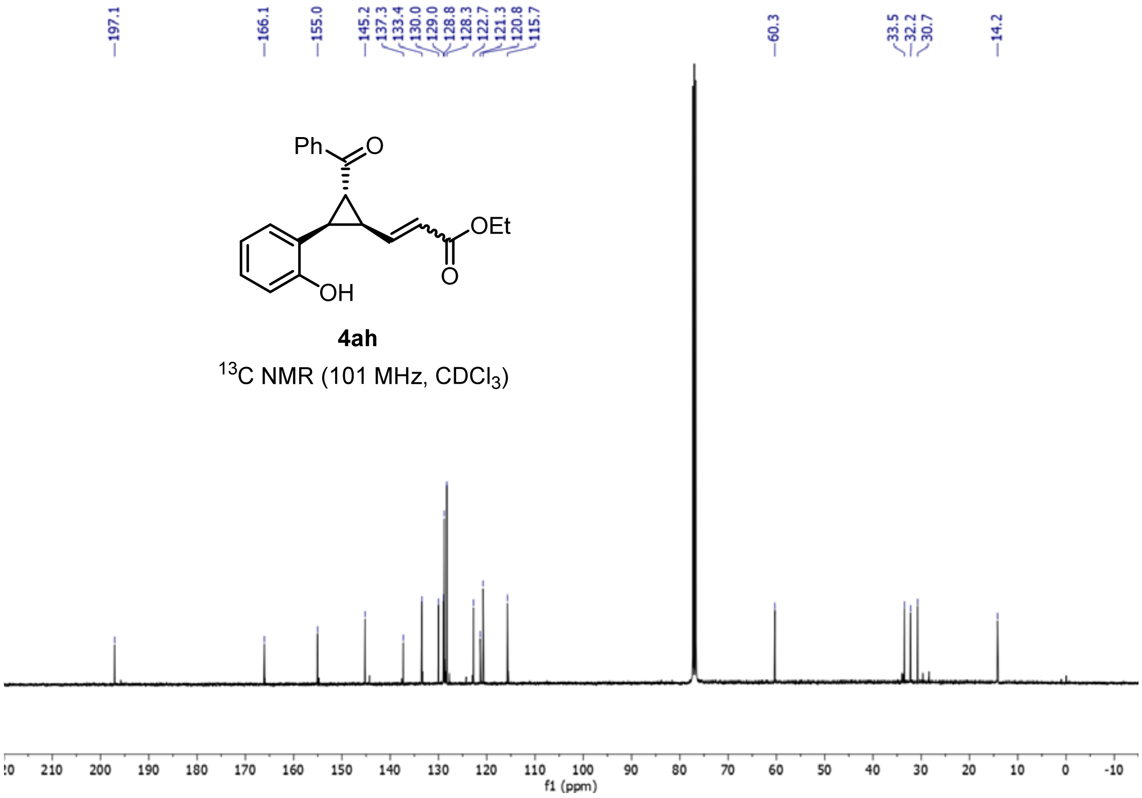

**Ethyl (1R,2S,3R)-2-(3-ethoxy-3-oxoprop-1-en-1-yl)-3-(2-hydroxy-4-methylphenyl)cyclopropane-1-carboxylate 4ba**

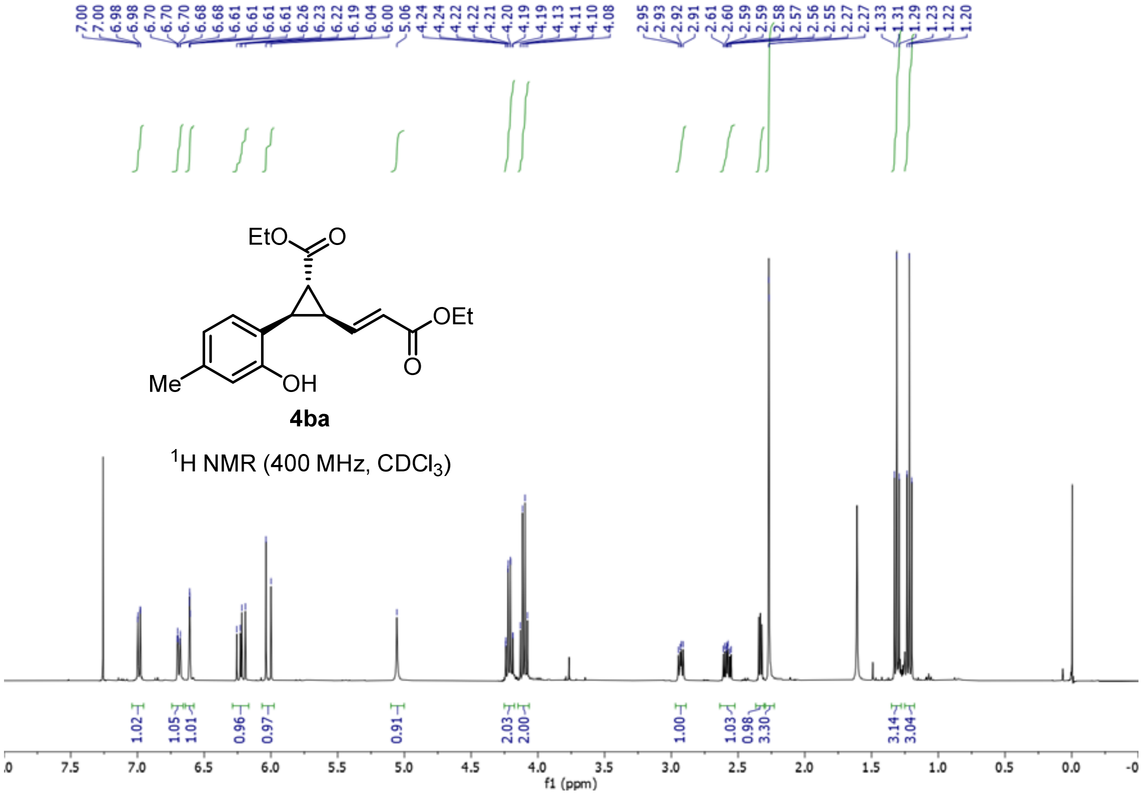


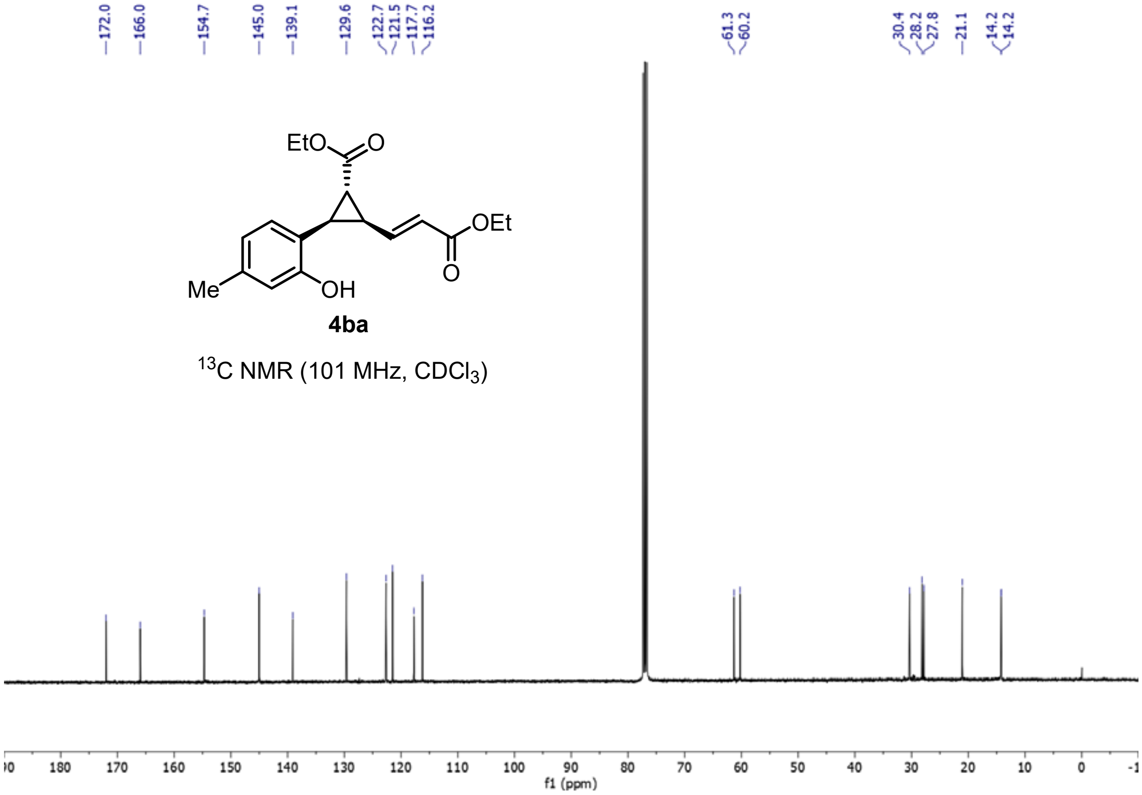

**Ethyl (1R,2S,3R)-2-(3-ethoxy-3-oxoprop-1-en-1-yl)-3-(2-hydroxy-5-methylphenyl)cyclopropane-1-carboxylate 4ca**

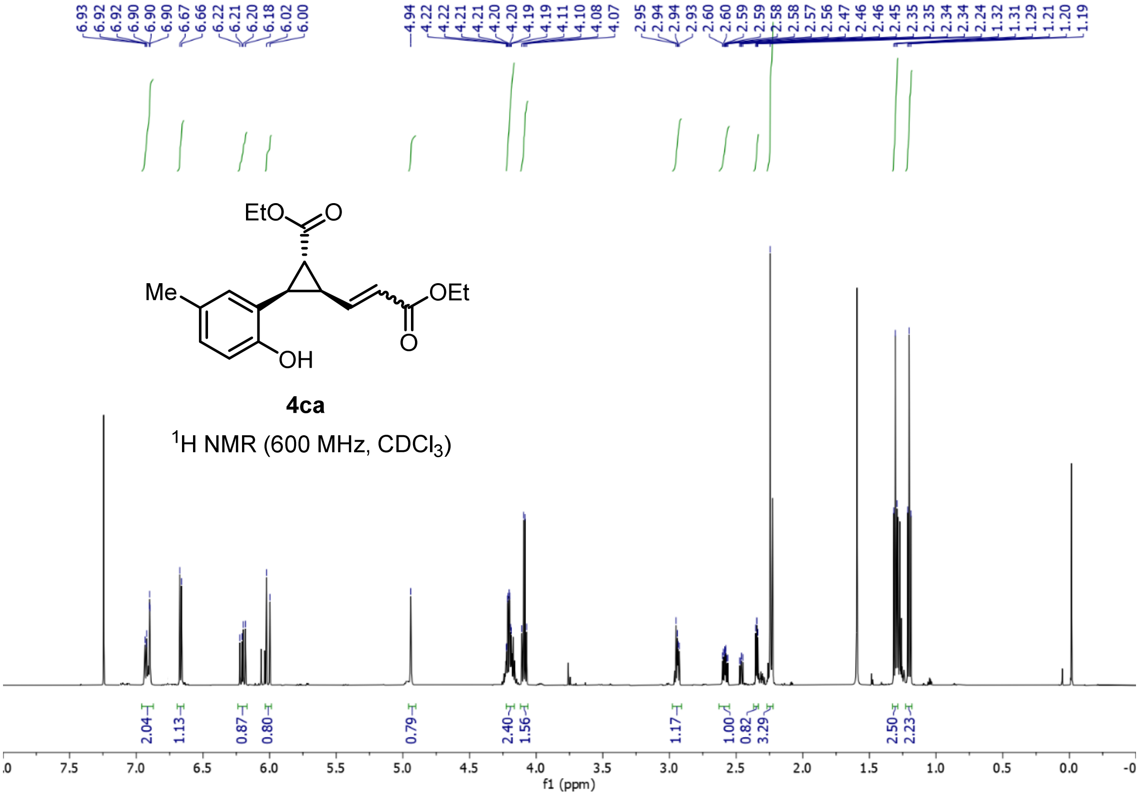


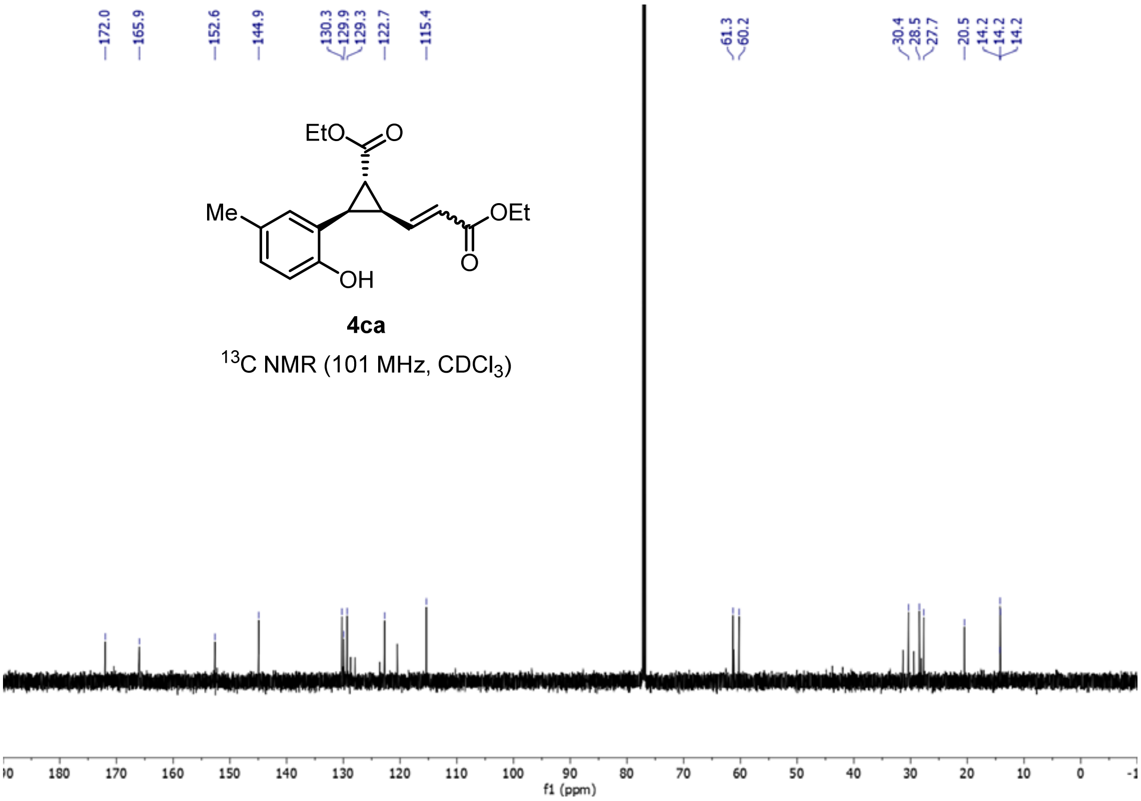

**Ethyl (1R,2S,3R)-2-(-3-ethoxy-3-oxoprop-1-en-1-yl)-3-(2-hydroxy-5-methoxyphenyl)cyclopropane -1-carboxylate 4da**

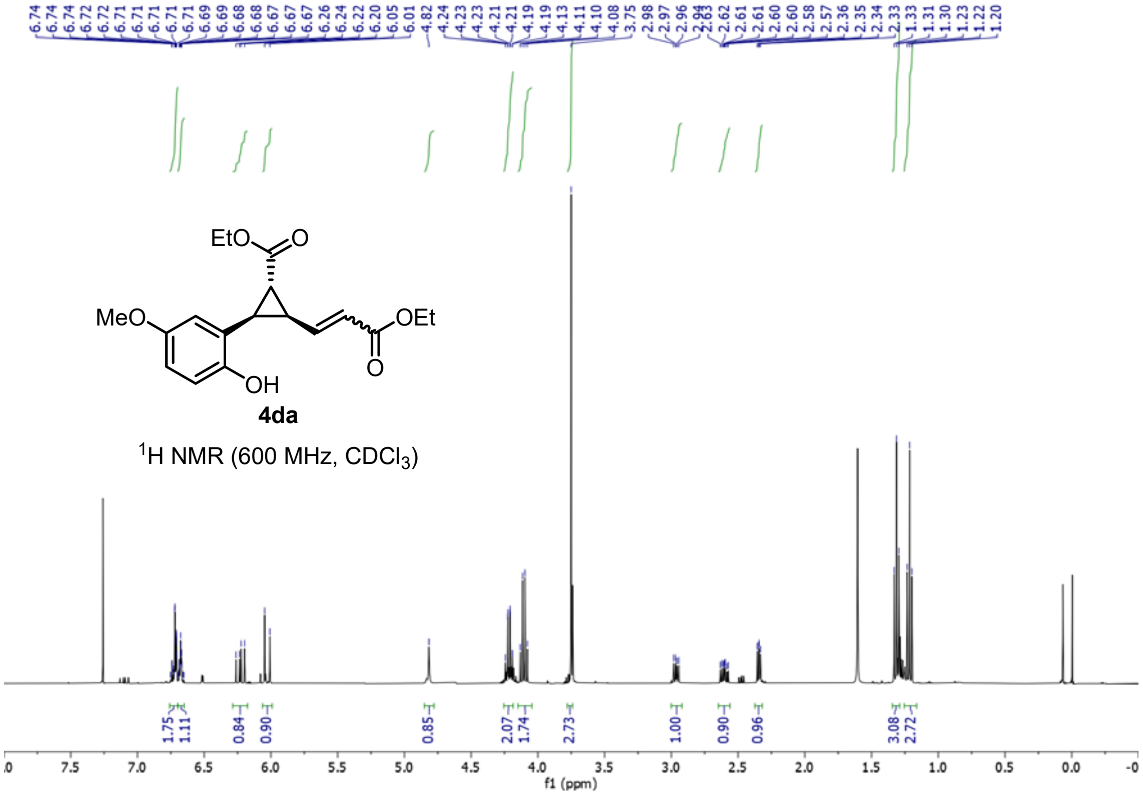


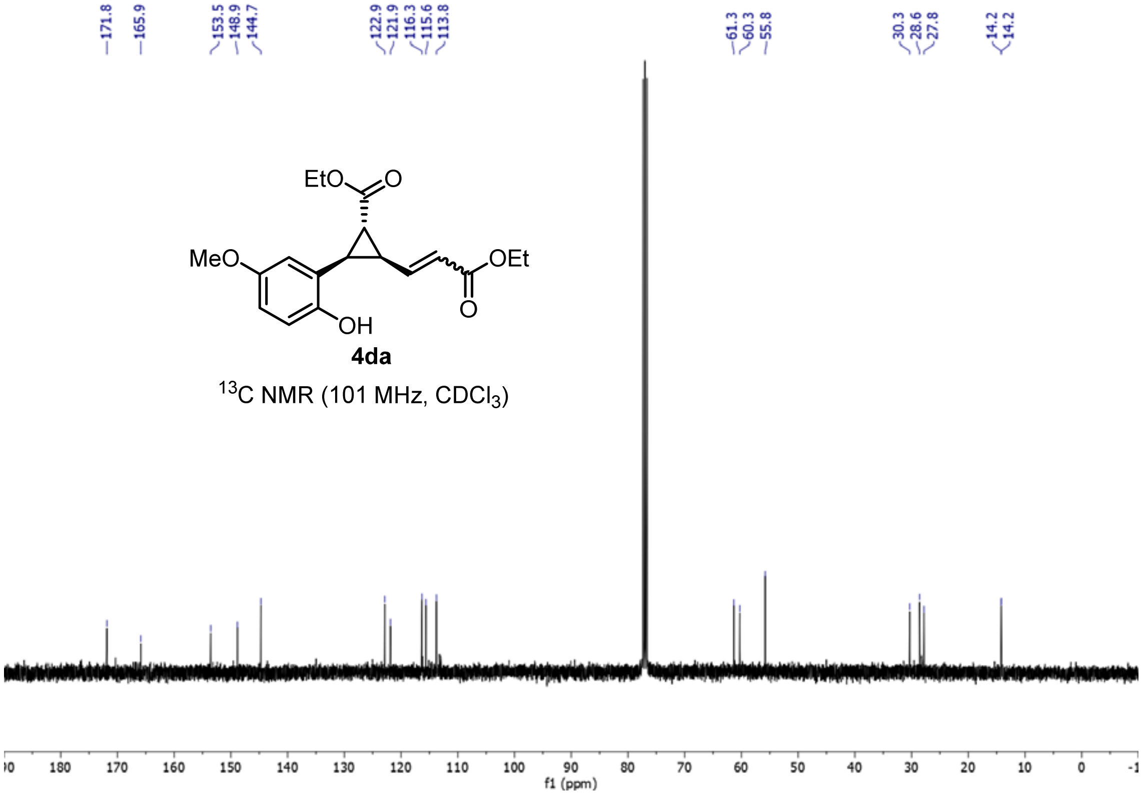

**Ethyl (1R,2R,3S)-2-(5-chloro-2-hydroxyphenyl)-3-(-3-ethoxy-3-oxoprop-1-en-1-yl)cyclopropane-1-carboxylate 4ea**

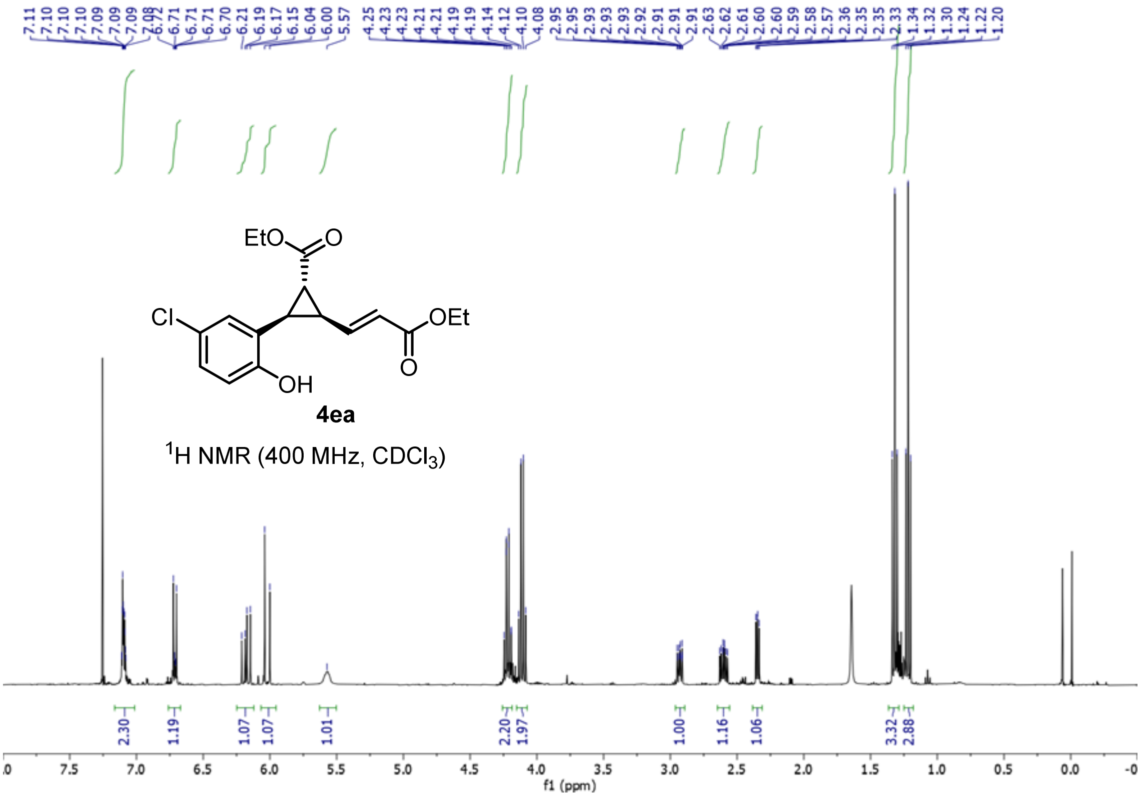


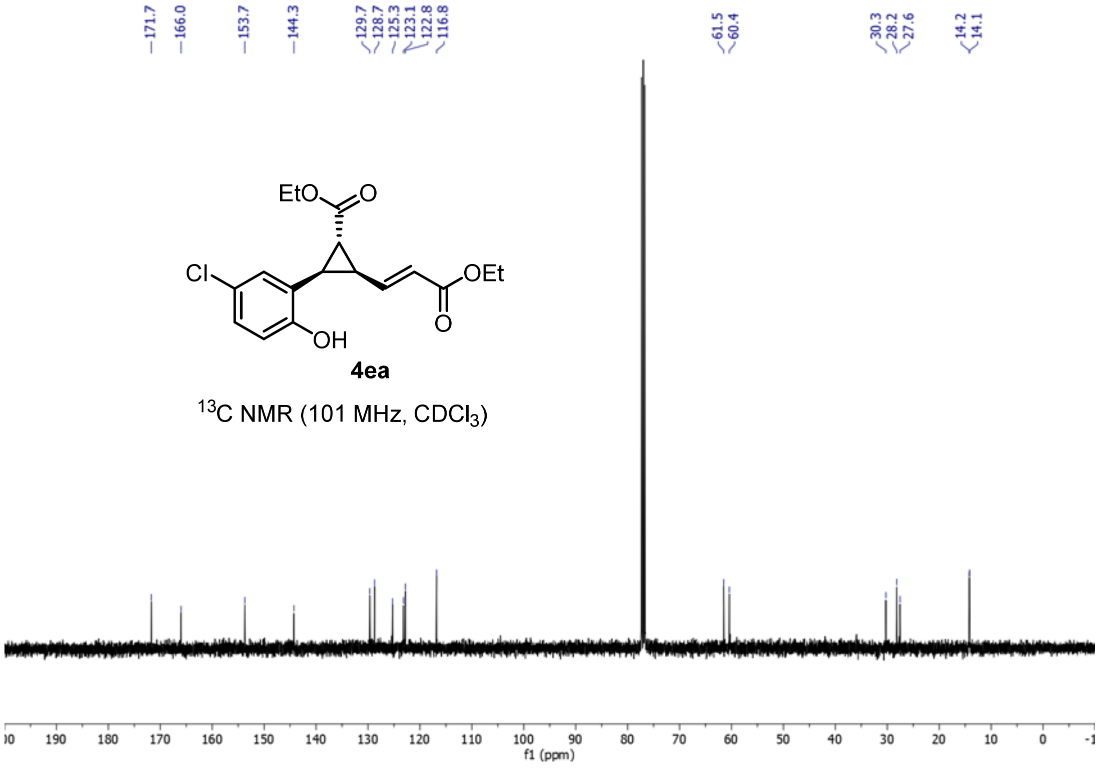

**Ethyl (1R,2S,3R)-2-(3-ethoxy-3-oxoprop-1-en-1-yl)-3-(2-hydroxy-4-methoxyphenyl)cyclopropane-1-carboxylate 4fa**

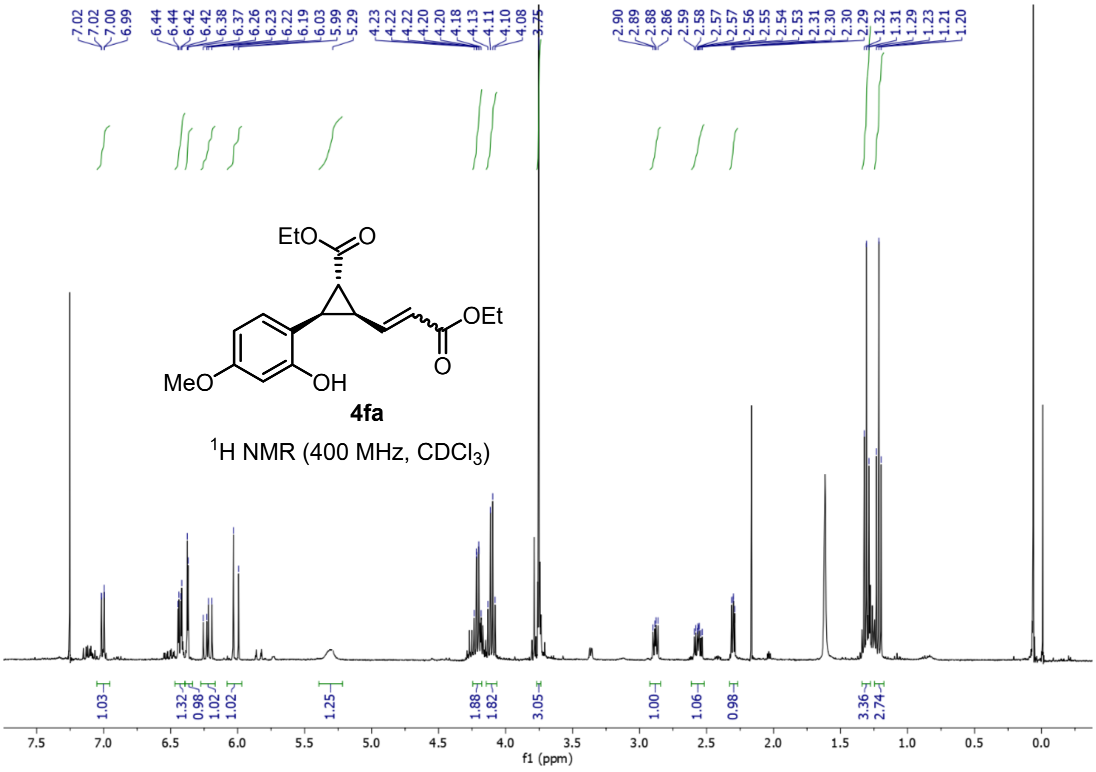


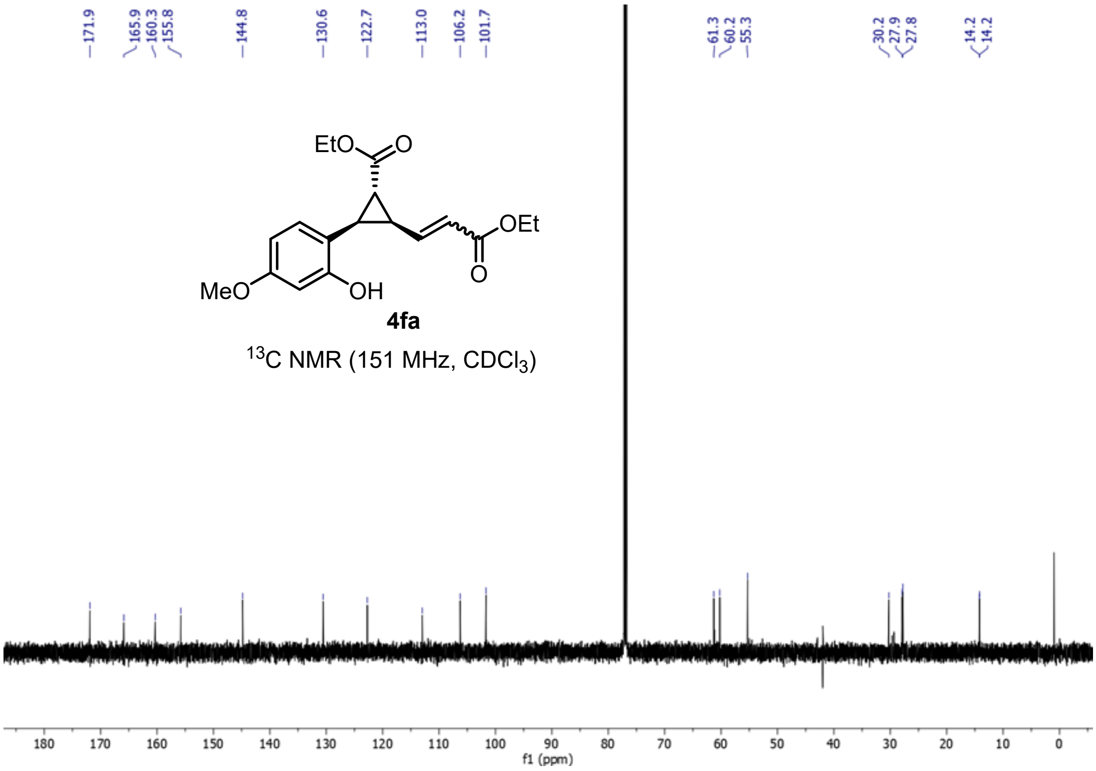

**Ethyl (1R,2S,3R)-2-(3-ethoxy-3-oxoprop-1-en-1-yl)-3-(2-hydroxy-3-methoxyphenyl)cyclopropane-1-carboxylate 4ga**

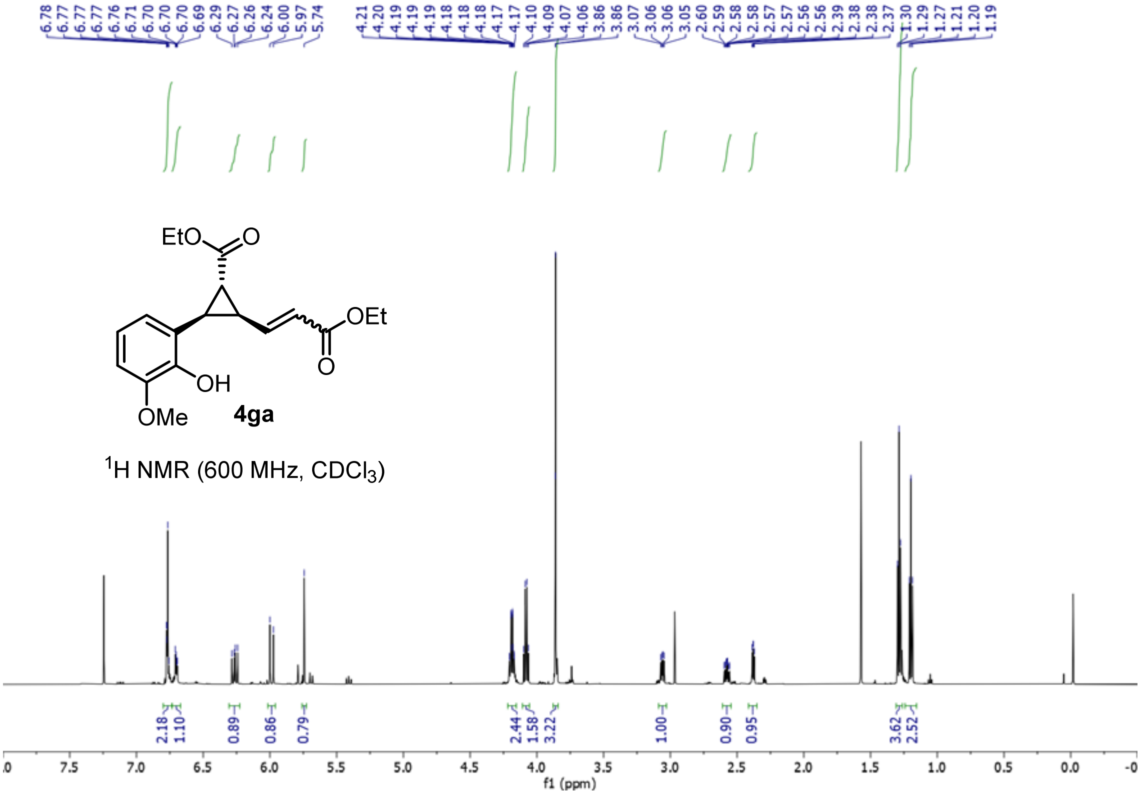


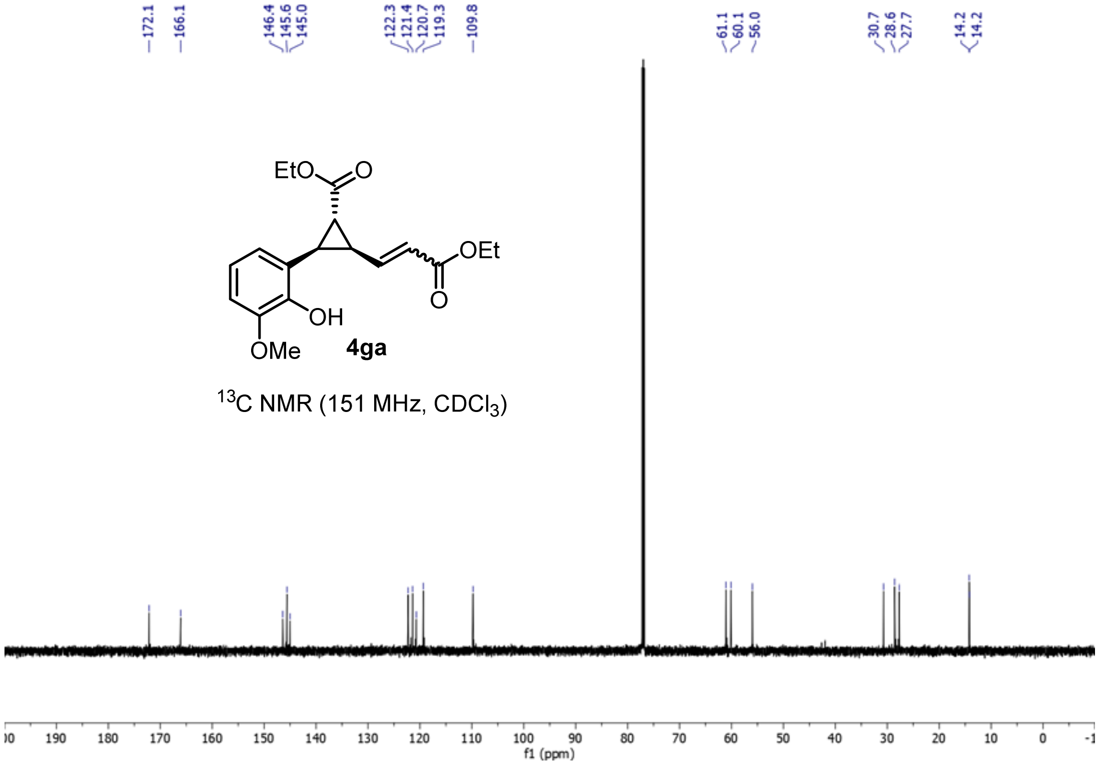

**Ethyl (1S,1aS,7bS)-2-oxo-1,1a,2,7b-tetrahydrocyclopropa[*c*]chromene-1-carboxylate 5aa**

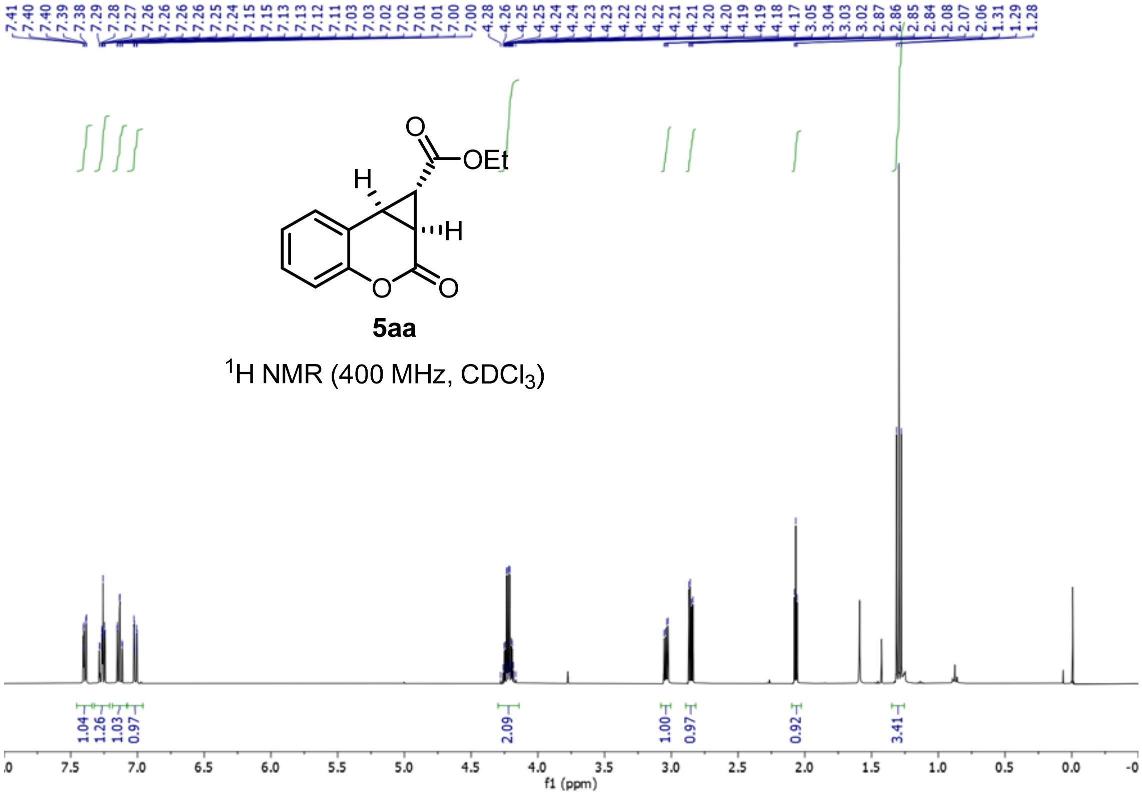


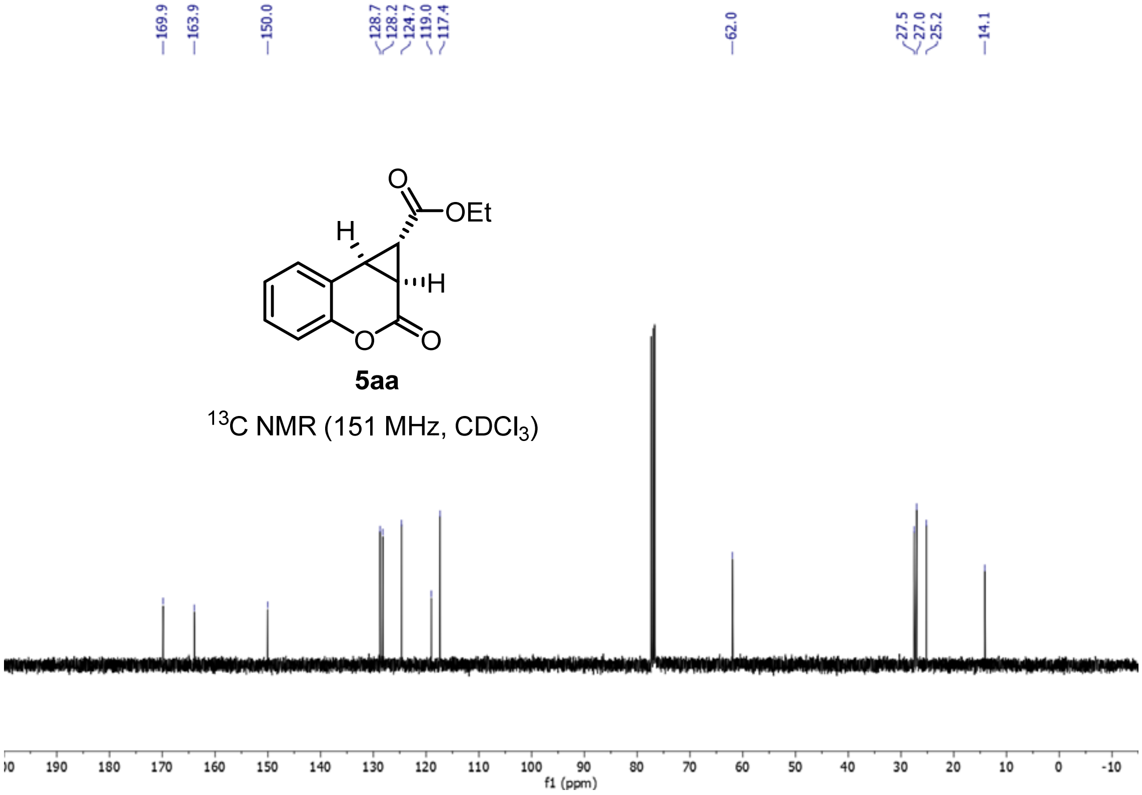

**Ethyl (1S,1aS,7bR)-1,1a,2,7b-tetrahydrocyclopropa[*c*]chromene-1-carboxylate 6aa**[13]

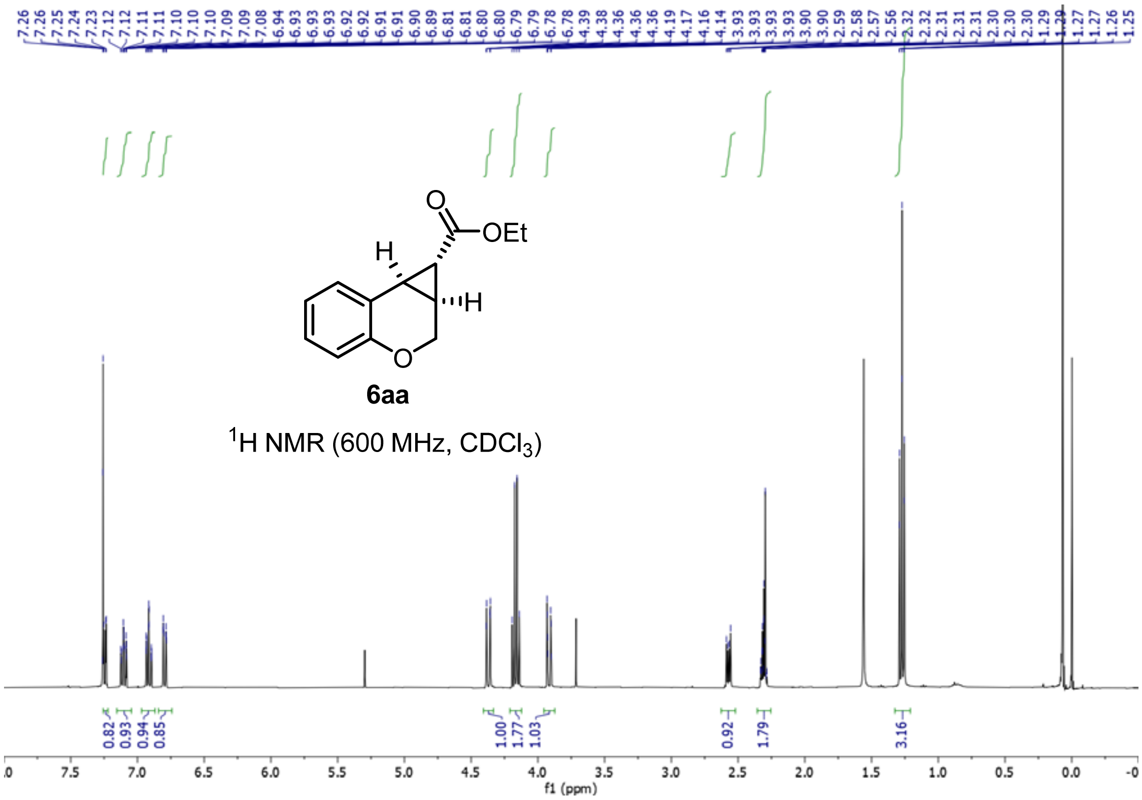


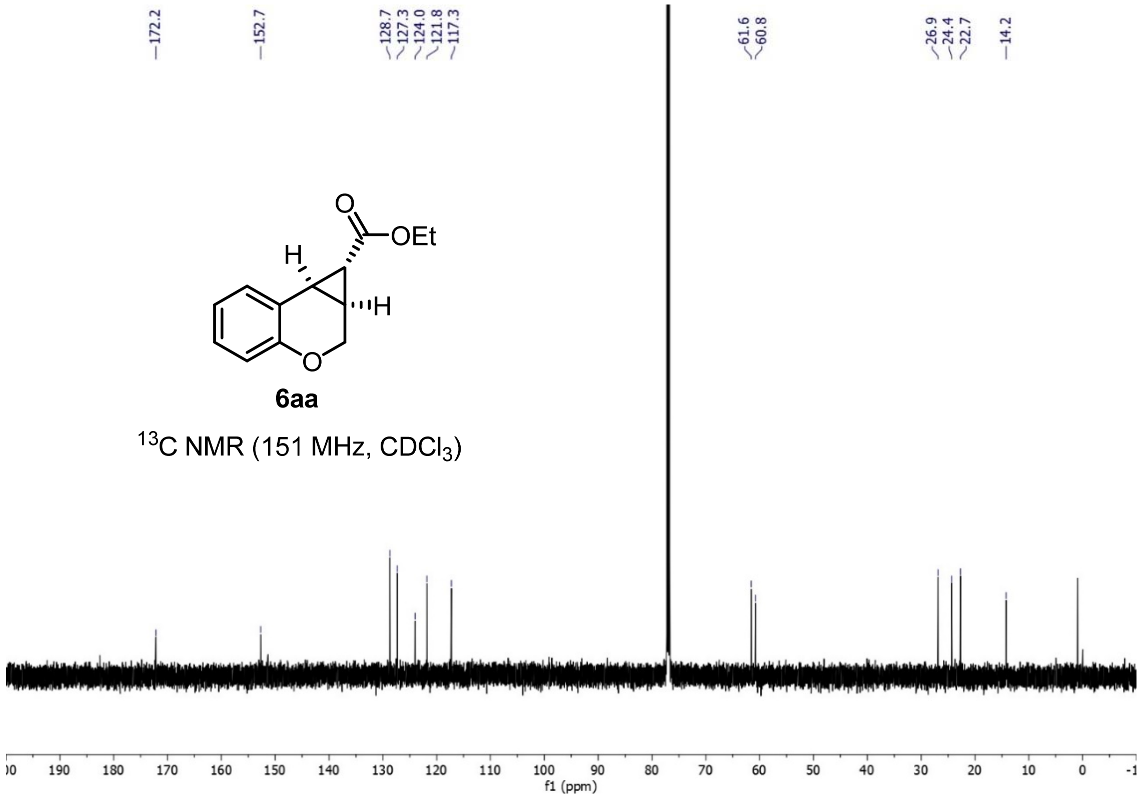


---

13 Racemic **6aa**: Ye, L.-W.; Sun, X.-L.; Li, C.-Y.; Tang, Y. *J. Org. Chem.* **2007**, *72*, 1335.

**Ethyl (1S,2S,3R)-2-(hydroxymethyl)-3-(2-hydroxyphenyl)cyclopropane-1-carboxylate 7aa**

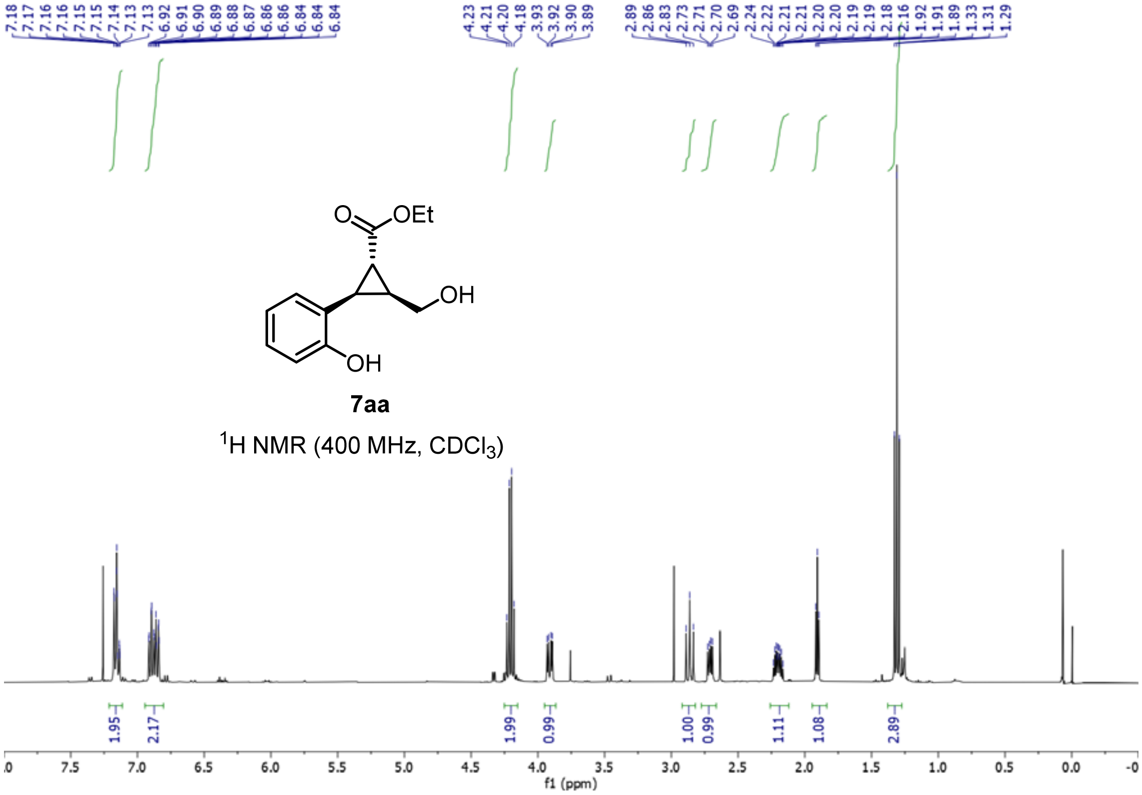


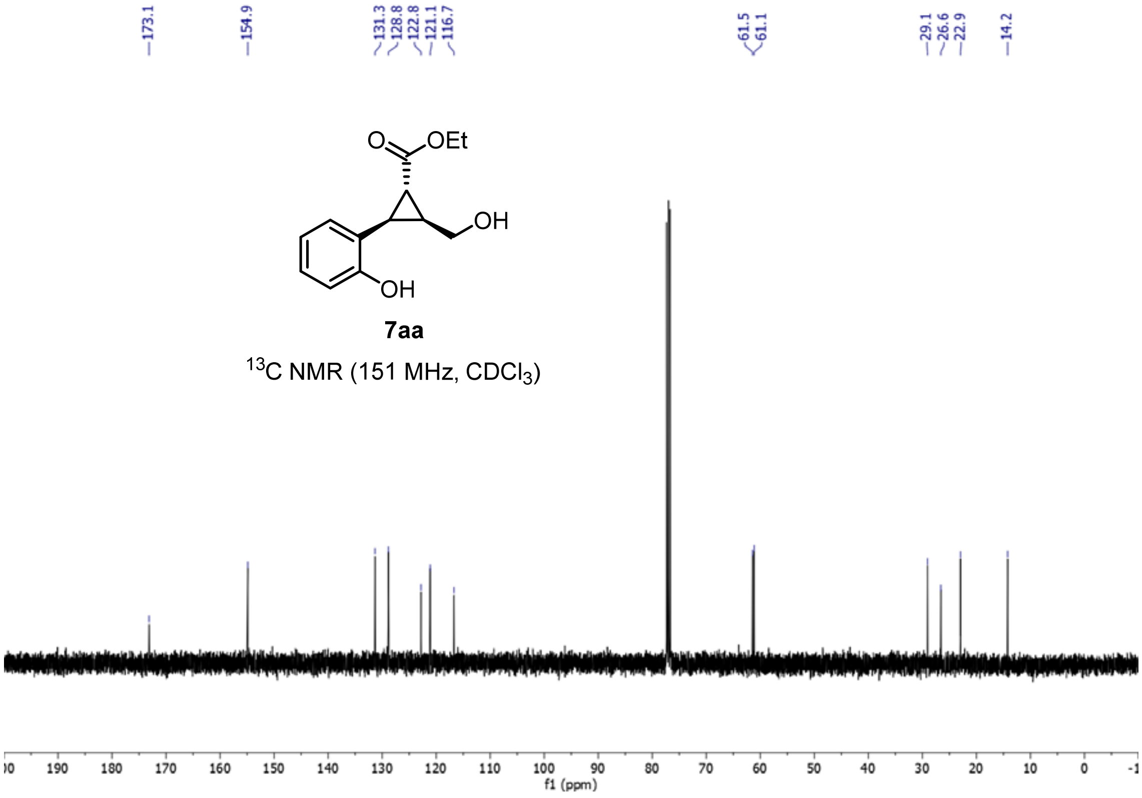

**Ethyl (1S,1aS,2R,7bR)-2-(2-ethoxy-2-oxoethyl)-1,1a,2,7b-tetrahydrocyclopropa[c]chromene-1-carboxylate *cis*-8aa**

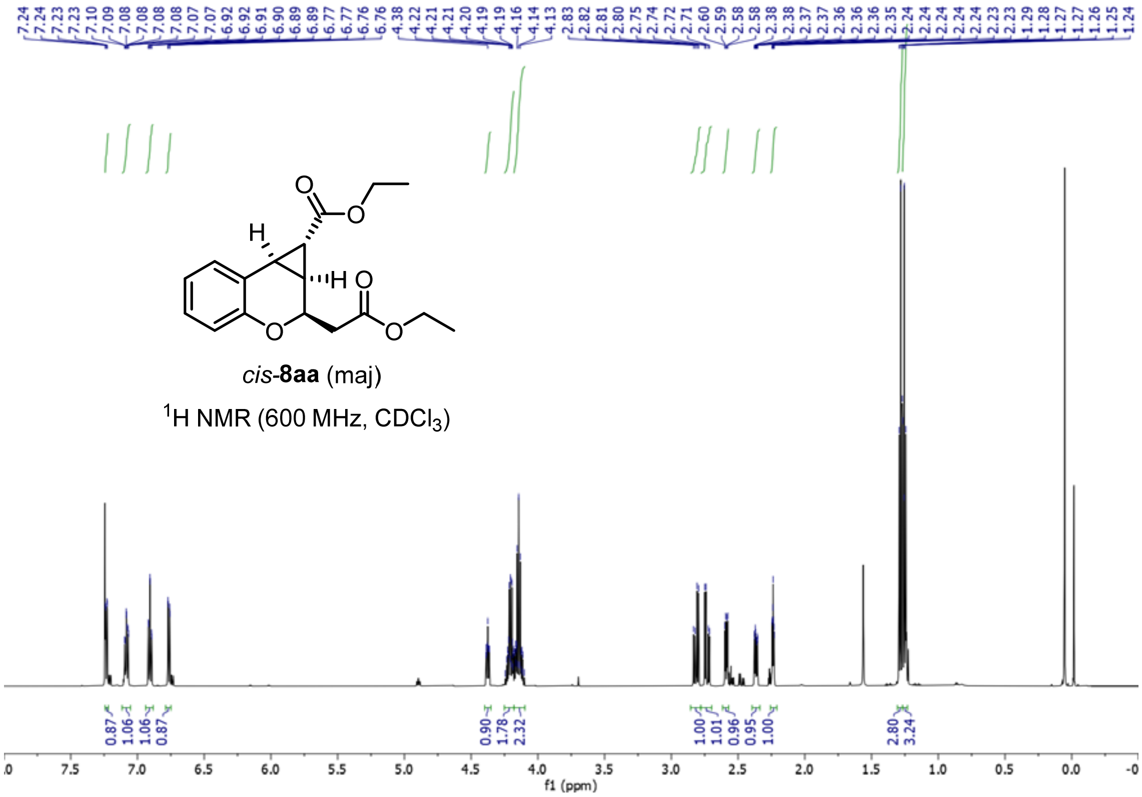


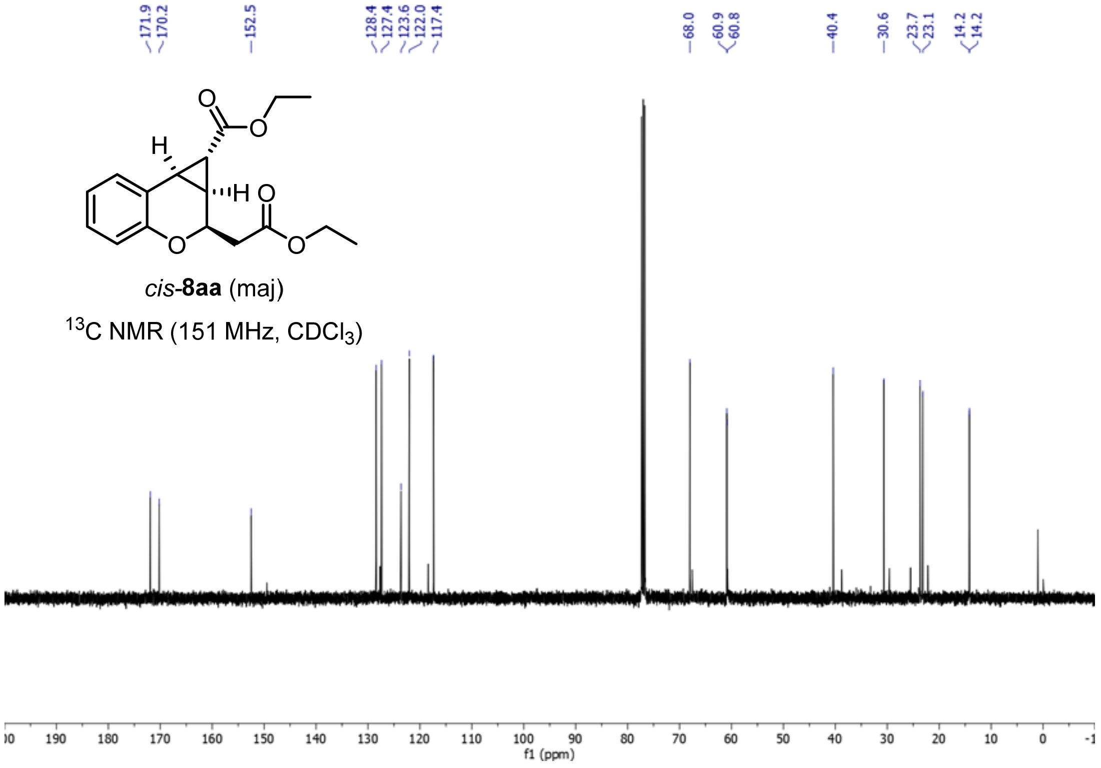

**Ethyl (1S,1aS,2S,7bR)-2-(2-ethoxy-2-oxoethyl)-1,1a,2,7b-tetrahydrocyclopropa[c]chromene-1-carboxylate *trans*-8aa**

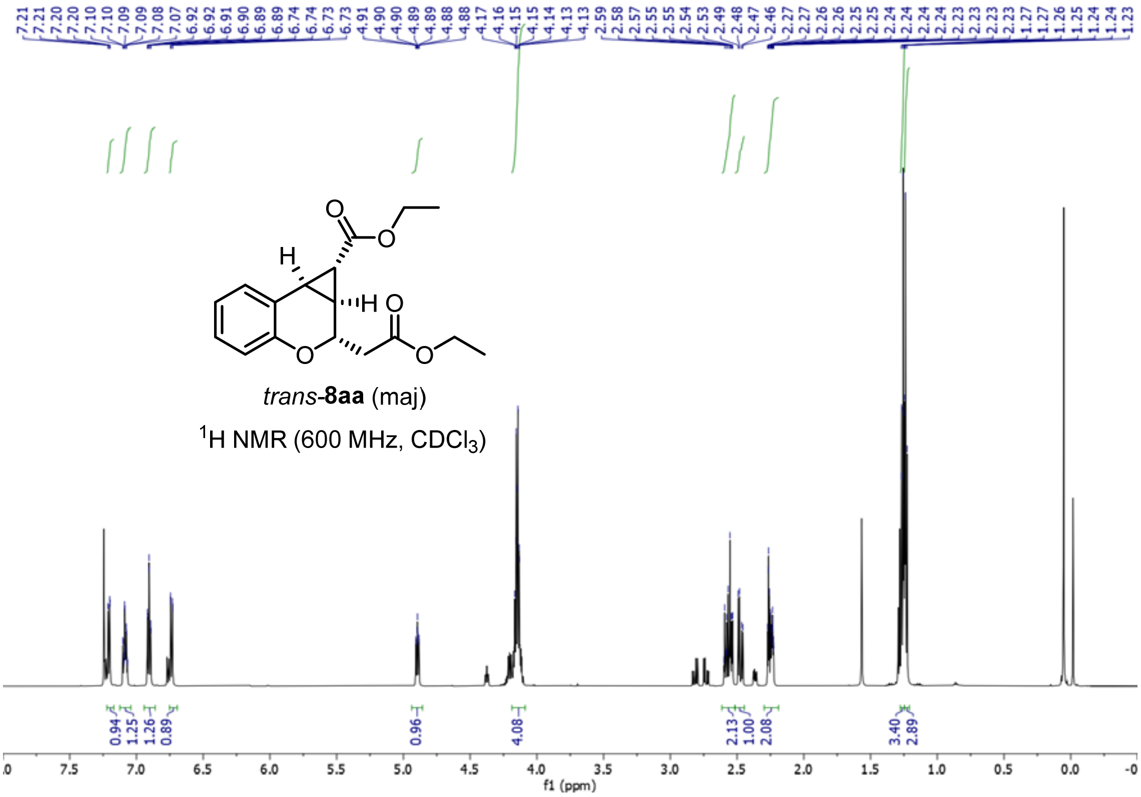


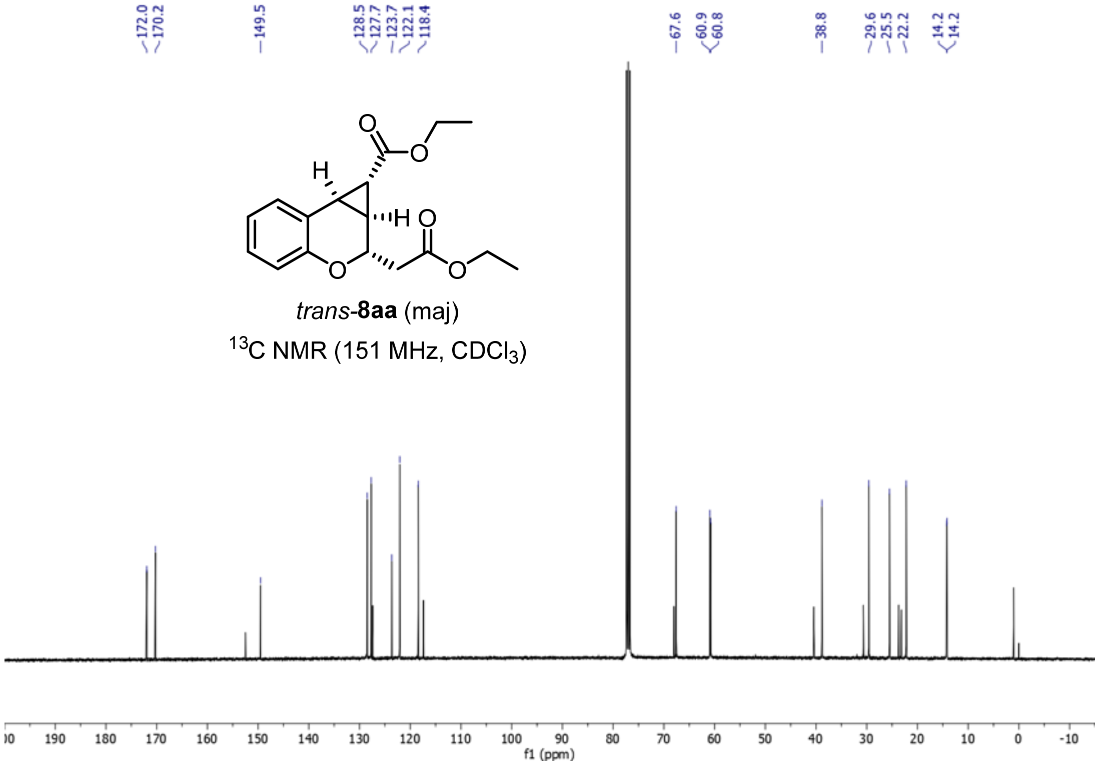

## Copies of HPLC traces of products 4-8

**Ethyl (1R*,2S*,3R*)-2-(3-ethoxy-3-oxoprop-1-en-1-yl)-3-(2-hydroxyphenyl)cyclopropane-1-carboxylate *rac*-4aa**

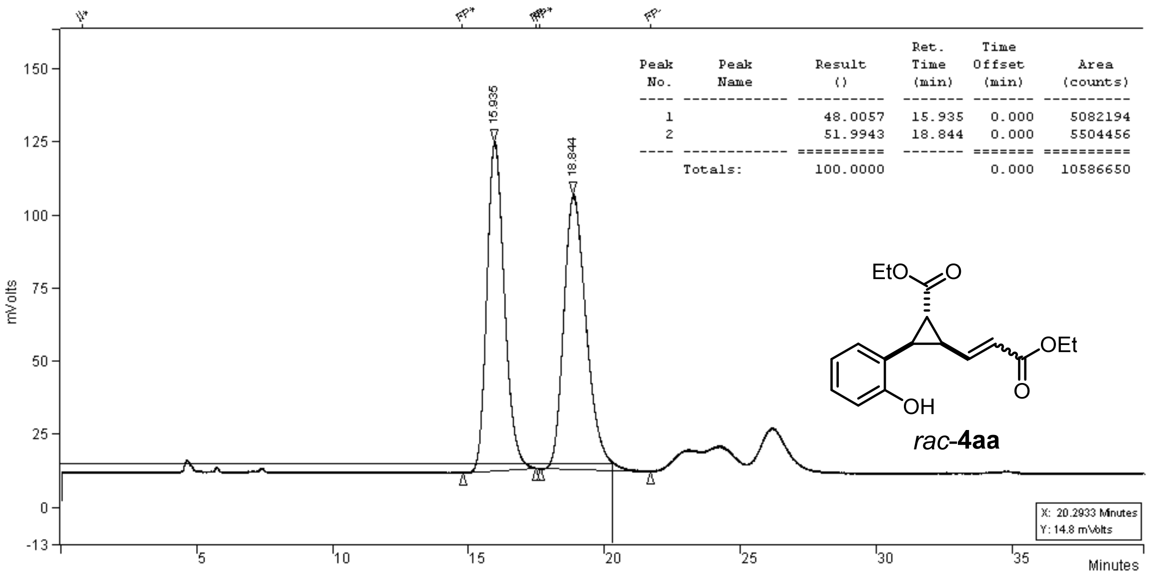


**Ethyl (1R,2S,3R)-2-(3-ethoxy-3-oxoprop-1-en-1-yl)-3-(2-hydroxyphenyl)cyclopropane-1-carboxylate 4aa**

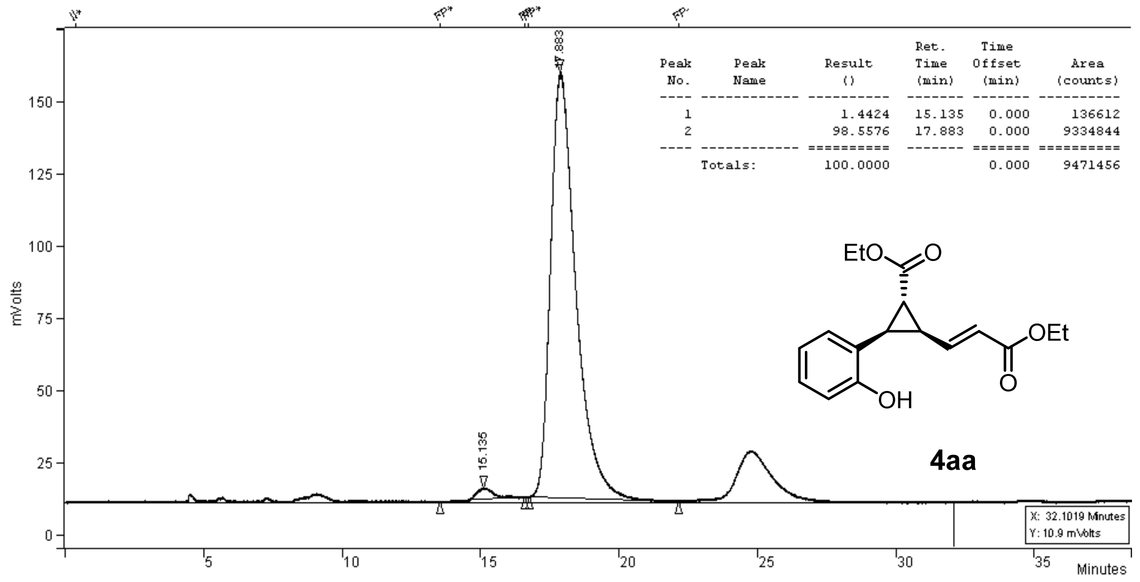

**Methyl (1R*,2S*,3R*)-2-(3-ethoxy-3-oxoprop-1-en-1-yl)-3-(2-hydroxyphenyl)cyclopropane-1-carboxylate *rac*-4ab**

| Peak # | RetTime [min] | Type | Width [min] | Area [mAU*s] | Height [mAU] | Area % |
|---|---|---|---|---|---|---|
| 1 | 7.493 | BB | 0.1851 | 1246.63782 | 104.53787 | 55.8733 |
| 2 | 9.118 | BV | 0.2300 | 984.54730 | 66.77629 | 44.1267 |

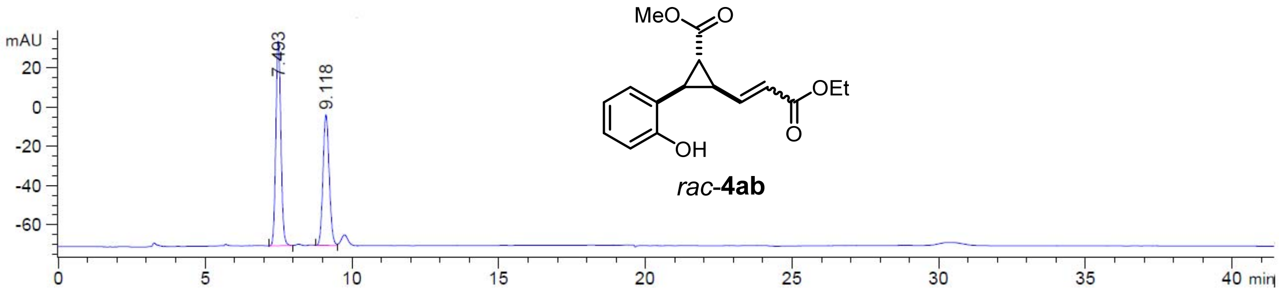


**Methyl (1R,2S,3R)-2-(3-ethoxy-3-oxoprop-1-en-1-yl)-3-(2-hydroxyphenyl)cyclopropane-1-carboxylate 4ab**

| Peak # | RetTime [min] | Type | Width [min] | Area [mAU*s] | Height [mAU] | Area % |
|---|---|---|---|---|---|---|
| 1 | 7.694 | BB | 0.1860 | 96.50211 | 8.15624 | 2.2479 |
| 2 | 9.355 | BV | 0.2402 | 4196.45410 | 271.73581 | 97.7521 |

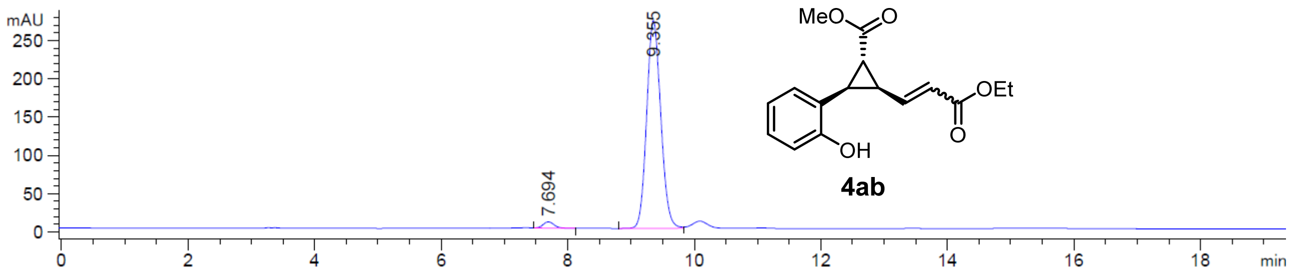

**Methyl (1R*,2R*,3S*)-2-(2-hydroxyphenyl)-3-(3-methoxy-3-oxoprop-1-en-1-yl)cyclopropane-1-carboxylate 4’ab**

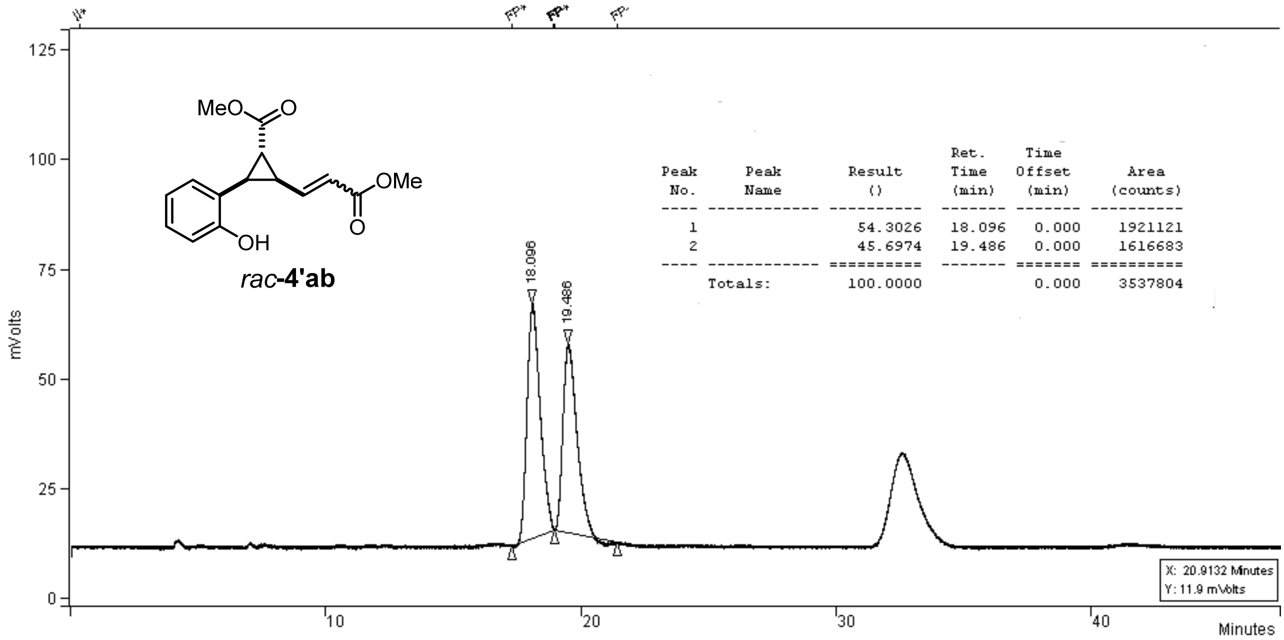


| Peak No. | Peak Name | Result () | Ret. Time (min) | Time Offset (min) | Area (counts) |
|---|---|---|---|---|---|
| 1 | | 54.3026 | 18.096 | 0.000 | 1921121 |
| 2 | | 45.6974 | 19.486 | 0.000 | 1616683 |
| | Totals: | 100.0000 | | 0.000 | 3537804 |

**Methyl (1R,2R,3S)-2-(2-hydroxyphenyl)-3-(3-methoxy-3-oxoprop-1-en-1-yl)cyclopropane-1-carboxylate 4’ab**

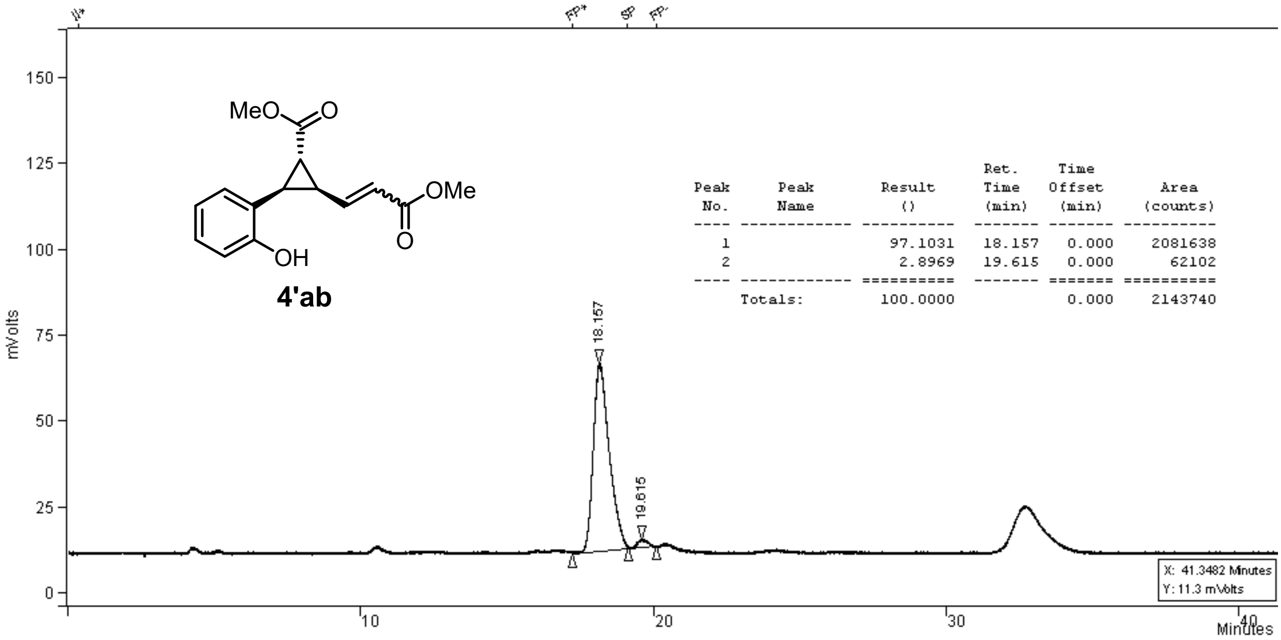


| Peak No. | Peak Name | Result () | Ret. Time (min) | Time Offset (min) | Area (counts) |
|---|---|---|---|---|---|
| 1 | | 97.1031 | 18.157 | 0.000 | 2081638 |
| 2 | | 2.8969 | 19.615 | 0.000 | 62102 |
| | Totals: | 100.0000 | | 0.000 | 2143740 |

**Butyl (1R*,2S*,3R*)-2-(3-ethoxy-3-oxoprop-1-en-1-yl)-3-(2-hydroxyphenyl)cyclopropane-1-carboxylatecarboxylate 4ac**

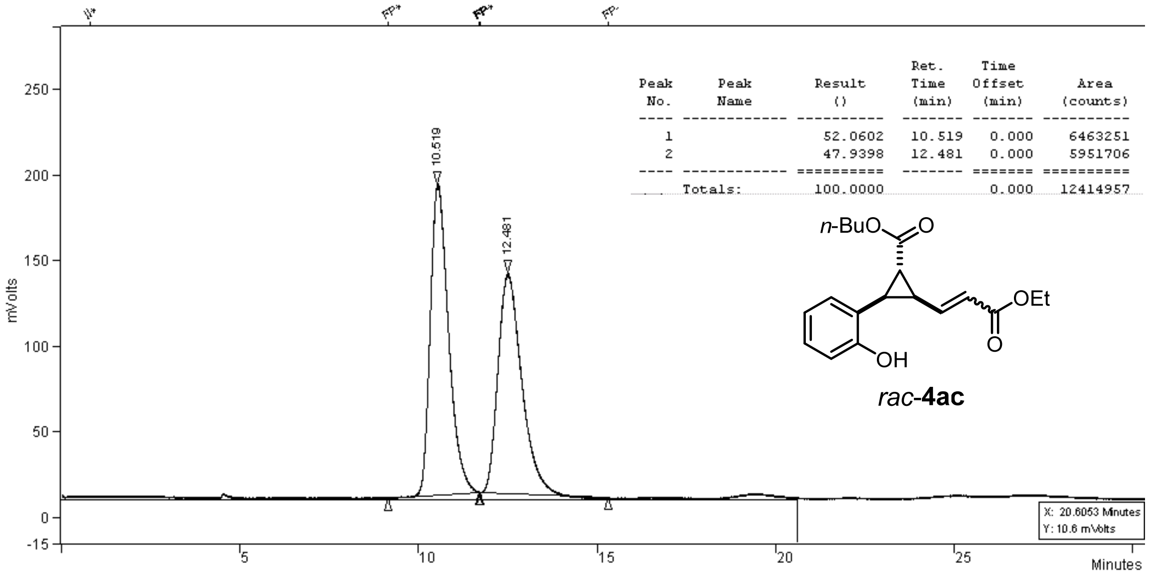


**Butyl (1R,2S,3R)-2-(3-ethoxy-3-oxoprop-1-en-1-yl)-3-(2-hydroxyphenyl)cyclopropane-1-carboxylatecarboxylate 4ac**

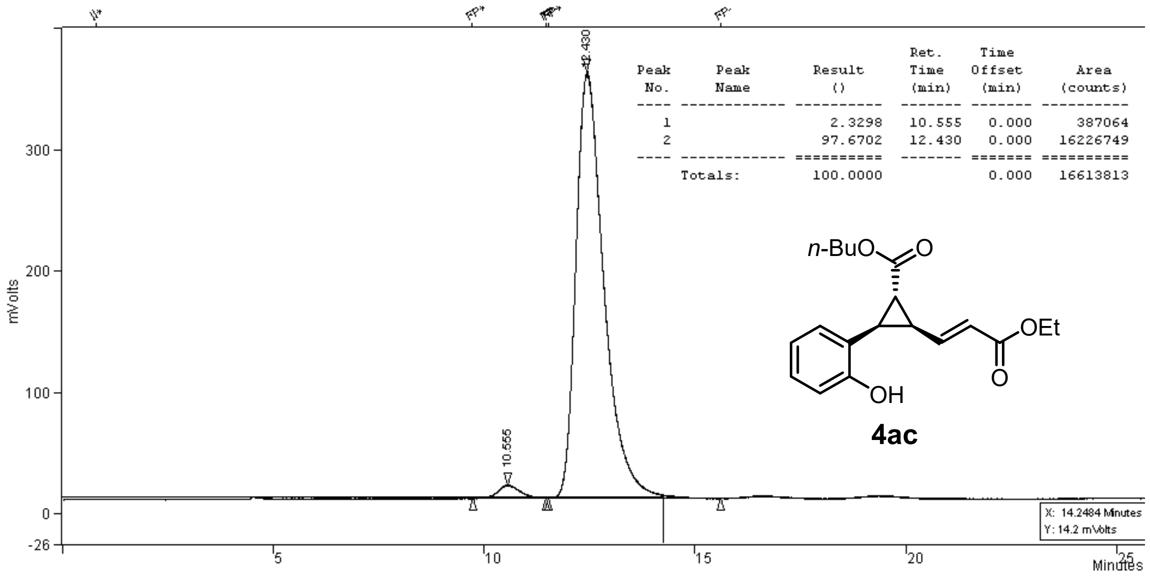

**Isobutyl (1R*,2S*,3R*)-2-(-3-ethoxy-3-oxoprop-1-en-1-yl)-3-(2-hydroxyphenyl)cyclopropane-1-carboxylate 4ad**

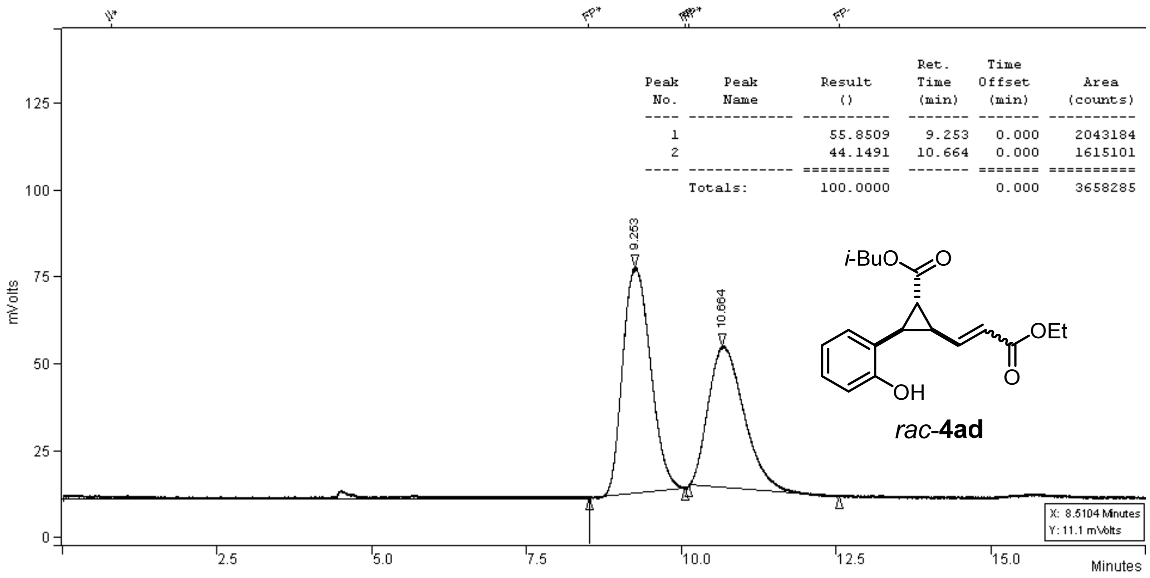


**Isobutyl (1R,2S,3R)-2-(-3-ethoxy-3-oxoprop-1-en-1-yl)-3-(2-hydroxyphenyl)cyclopropane-1-carboxylate 4ad**

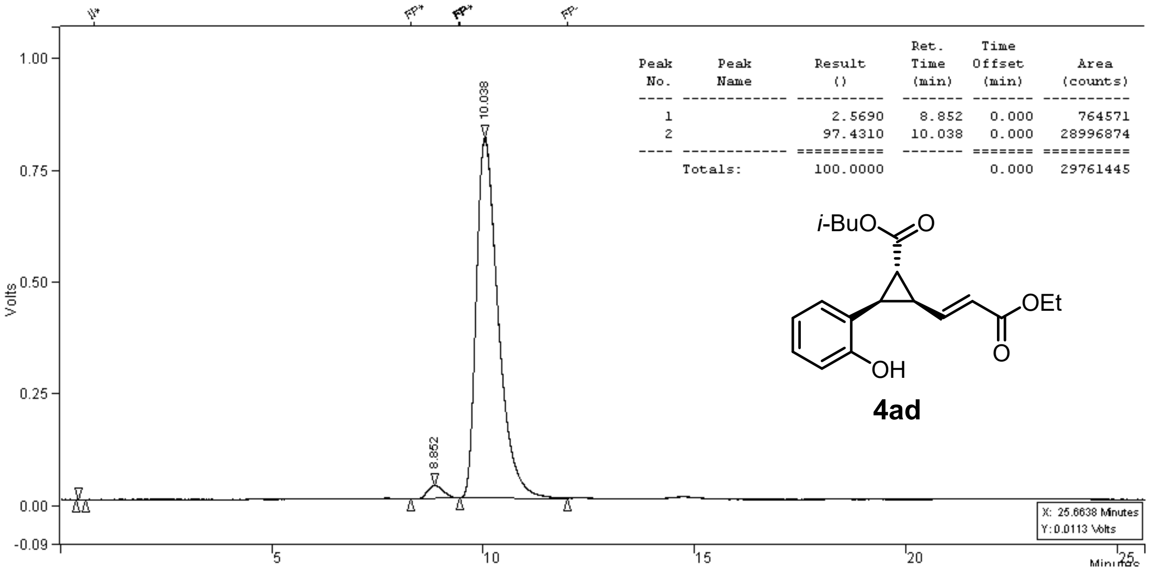

**Tert-butyl (1R*,2S*,3R*)-2-(-3-ethoxy-3-oxoprop-1-en-1-yl)-3-(2-hydroxyphenyl)cyclopropane-1-carboxylate 4ae**

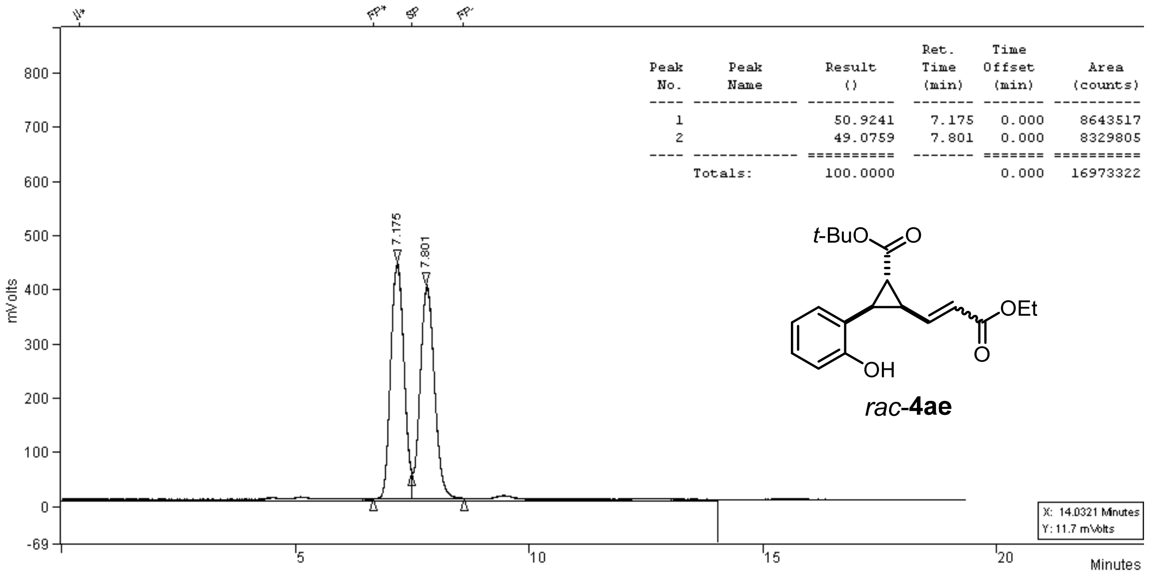


**Tert-butyl (1R,2S,3R)-2-(-3-ethoxy-3-oxoprop-1-en-1-yl)-3-(2-hydroxyphenyl)cyclopropane-1-carboxylate 4ae**

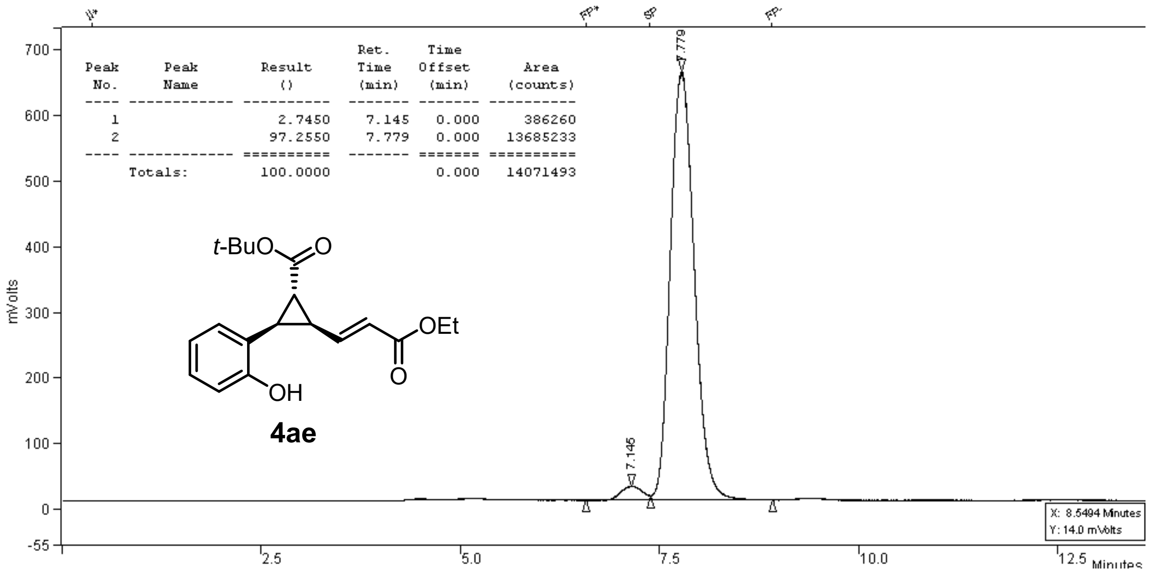

**Allyl (1R*,2S*,3R*)-2-(3-ethoxy-3-oxoprop-1-en-1-yl)-3-(2-hydroxyphenyl)cyclopropane-1-carboxylate 4af**

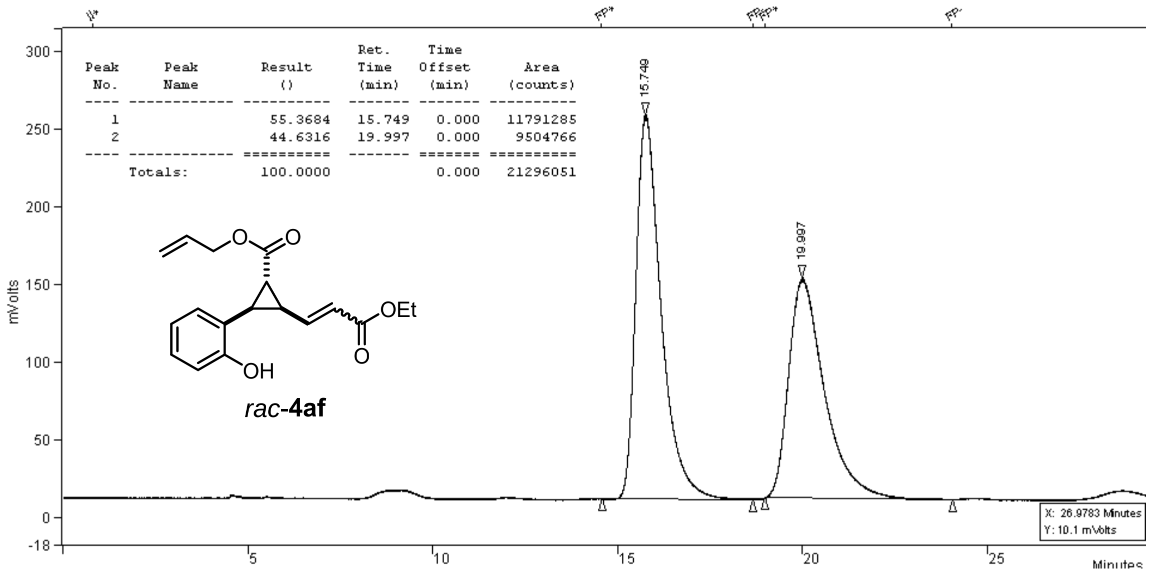


**Allyl (1R,2S,3R)-2-(3-ethoxy-3-oxoprop-1-en-1-yl)-3-(2-hydroxyphenyl)cyclopropane-1-carboxylate 4af**

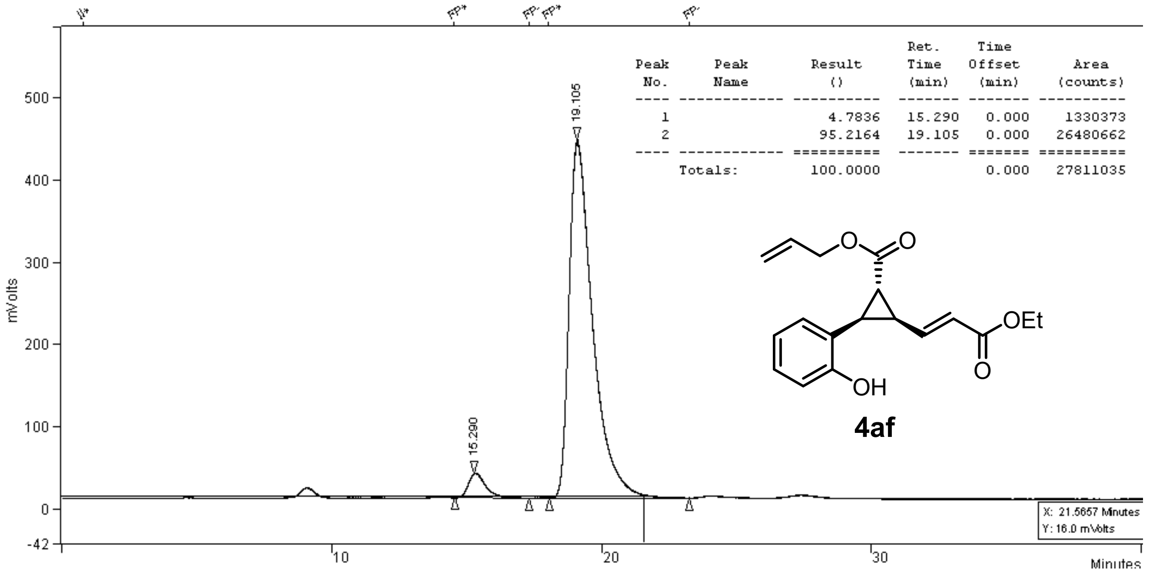

**Benzyl (1R*,2S*,3R*)-2-(3-ethoxy-3-oxoprop-1-en-1-yl)-3-(2-hydroxyphenyl)cyclopropane-1-carboxylate 4ag**

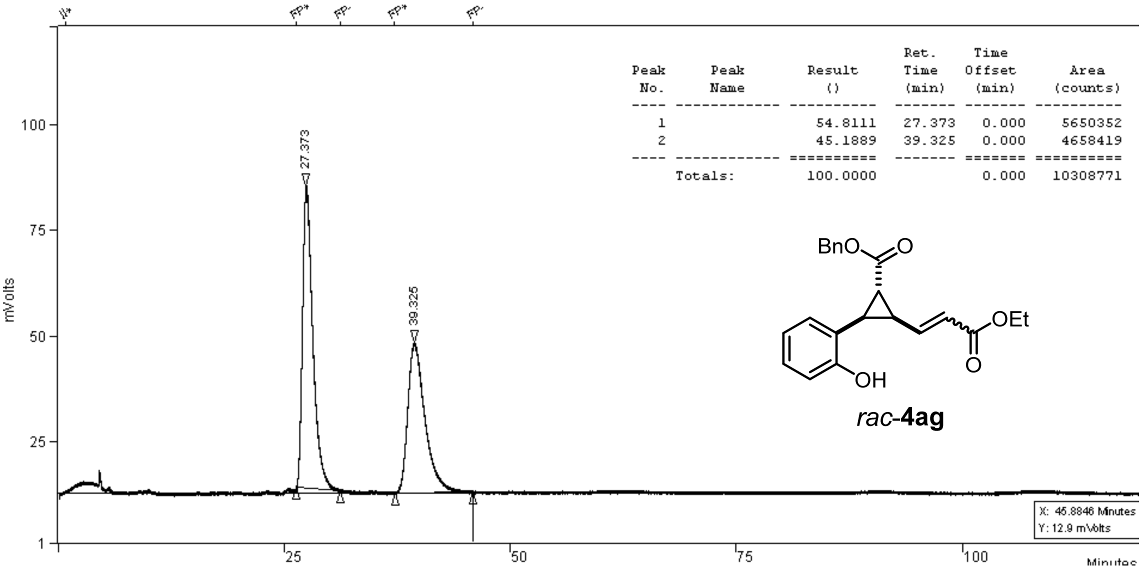

| Peak No. | Peak Name | Result () | Ret. Time (min) | Time Offset (min) | Area (counts) |
|---|---|---|---|---|---|
| 1 | | 54.8111 | 27.373 | 0.000 | 5650352 |
| 2 | | 45.1889 | 39.325 | 0.000 | 4658419 |
| | Totals: | 100.0000 | | 0.000 | 10308771 |



**Benzyl (1R,2S,3R)-2-(3-ethoxy-3-oxoprop-1-en-1-yl)-3-(2-hydroxyphenyl)cyclopropane-1-carboxylate 4af**

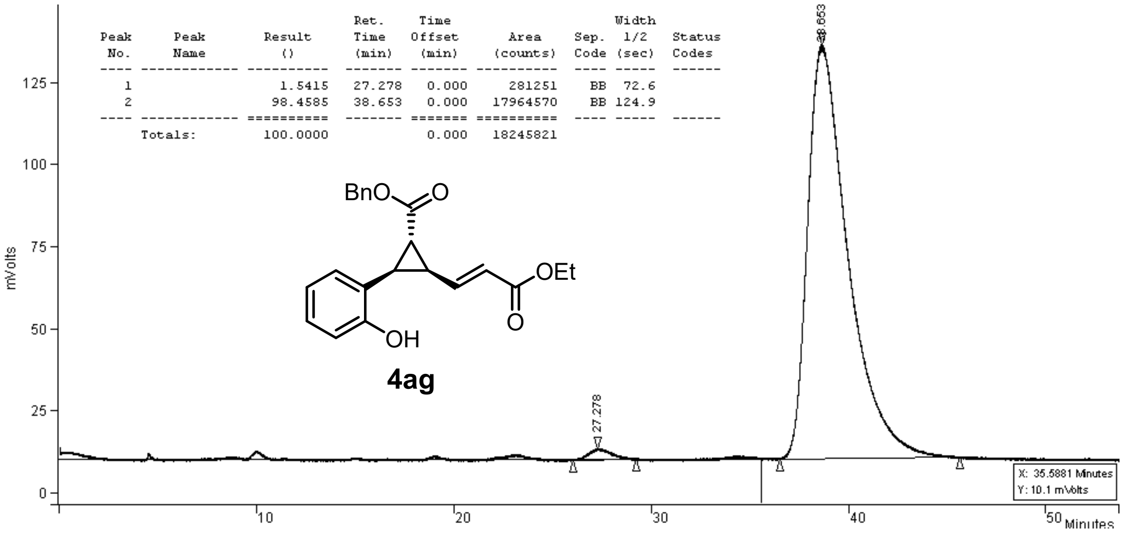

| Peak No. | Peak Name | Result () | Ret. Time (min) | Time Offset (min) | Area (counts) | Sep. Code | Width 1/2 (sec) | Status Codes |
|---|---|---|---|---|---|---|---|---|
| 1 | | 1.5415 | 27.278 | 0.000 | 281251 | BB | 72.6 | |
| 2 | | 98.4585 | 38.653 | 0.000 | 17964570 | BB | 124.9 | |
| | Totals: | 100.0000 | | 0.000 | 18245821 | | | |

**Ethyl 3-((1S*,2R*,3R*)-2-benzoyl-3-(2-hydroxyphenyl)cyclopropyl)acrylate *rac*-4ah**

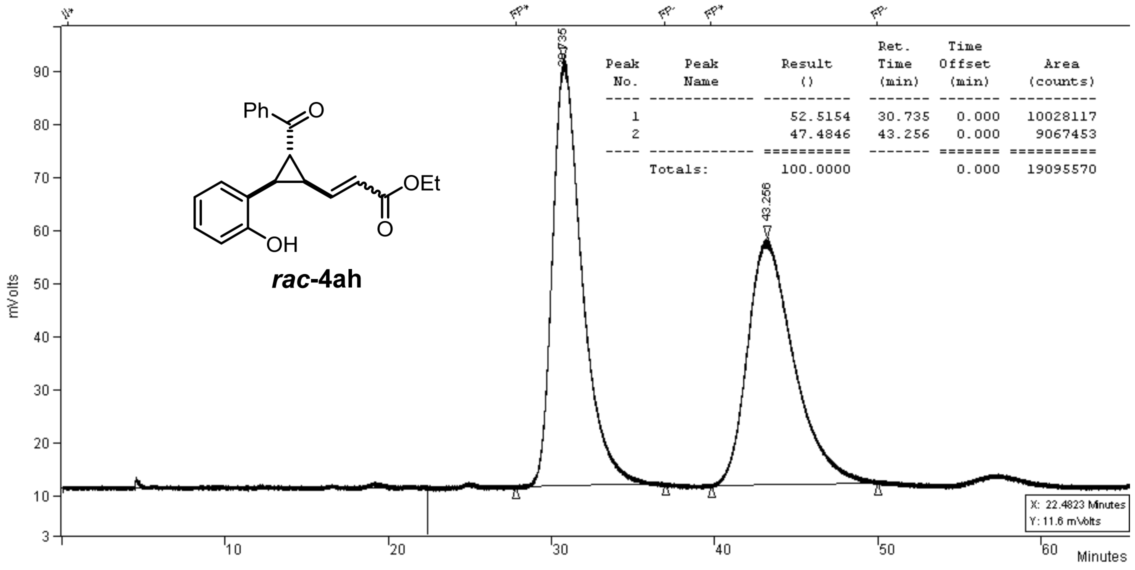


**Ethyl 3-((1S,2R,3R)-2-benzoyl-3-(2-hydroxyphenyl)cyclopropyl)acrylate 4ah**

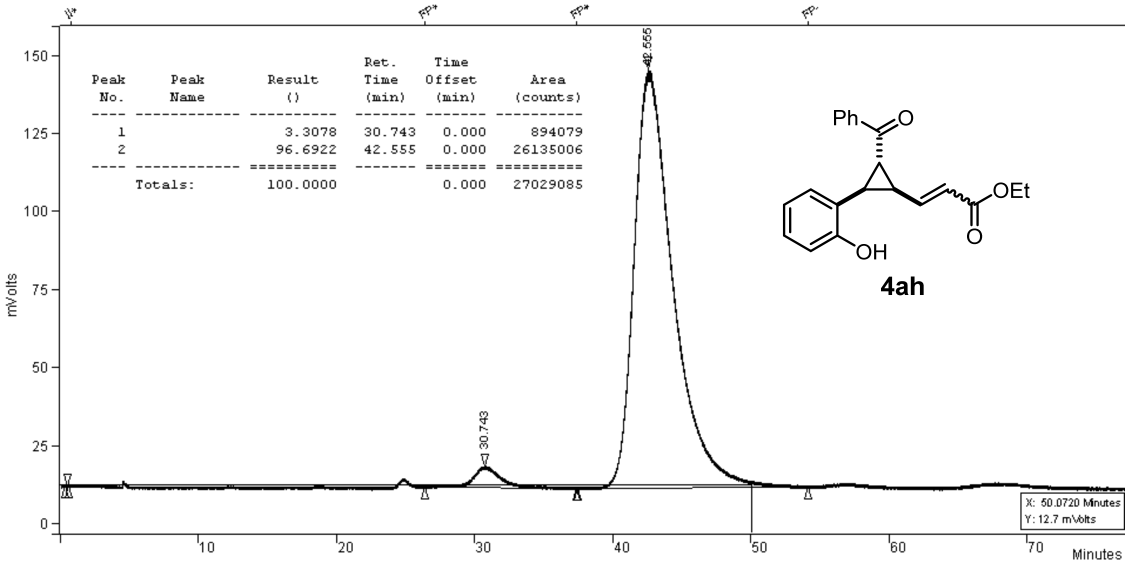

**Ethyl (1R*,2S*,3R*)-2-(3-ethoxy-3-oxoprop-1-en-1-yl)-3-(2-hydroxy-4-methylphenyl)cyclopropane-1-carboxylate 4ba**

| Peak # | RetTime [min] | Type | Width [min] | Area [mAU*s] | Height [mAU] | Area % |
|---|---|---|---|---|---|---|
| 1 | 7.072 | VV | 0.1755 | 1119.23657 | 96.39005 | 55.3514 |
| 2 | 8.791 | BV | 0.2252 | 902.81921 | 62.24410 | 44.6486 |
| Totals : | | | | 2022.05579 | 158.63414 | |

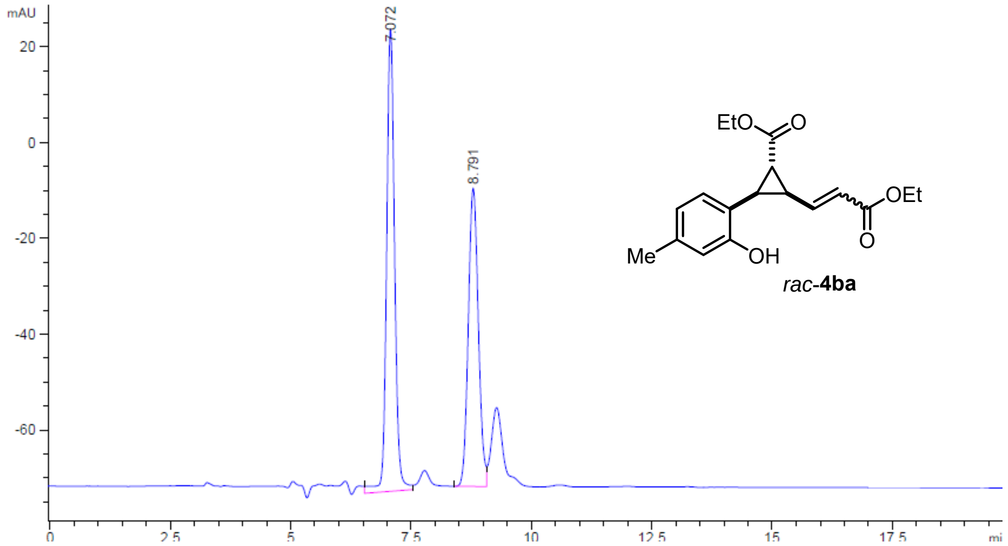


**Ethyl (1R,2S,3R)-2-(3-ethoxy-3-oxoprop-1-en-1-yl)-3-(2-hydroxy-4-methylphenyl)cyclopropane-1-carboxylate 4da**

| Peak # | RetTime [min] | Type | Width [min] | Area [mAU*s] | Height [mAU] | Area % |
|---|---|---|---|---|---|---|
| 1 | 7.075 | BB | 0.1790 | 34.59105 | 2.94582 | 3.7611 |
| 2 | 8.805 | BV | 0.2255 | 885.12604 | 60.93709 | 96.2389 |
| Totals : | | | | 919.71708 | 63.88291 | |

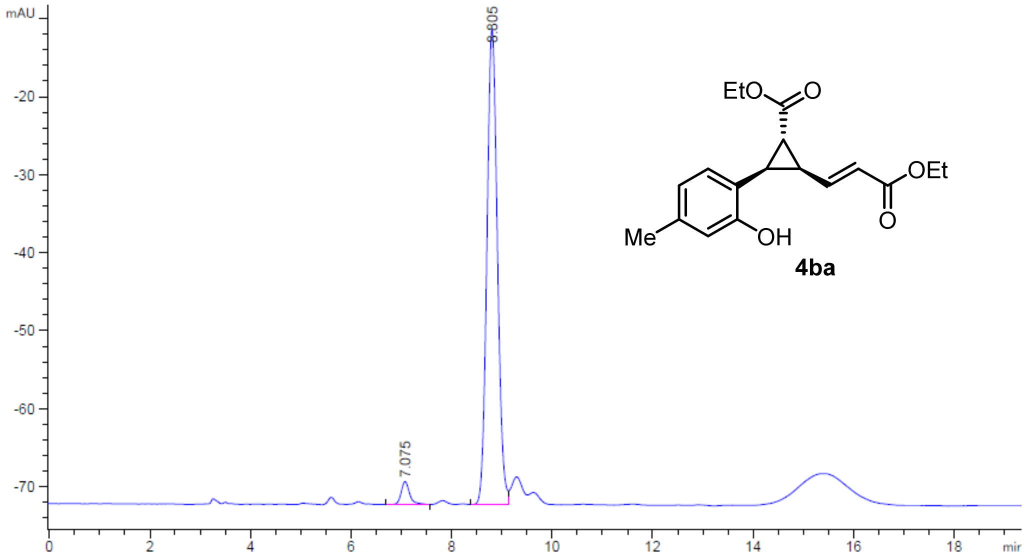

**Ethyl (1R*,2S*,3R*)-2-(3-ethoxy-3-oxoprop-1-en-1-yl)-3-(2-hydroxy-5-methylphenyl)cyclopropane-1-carboxylate 4ca**

| Peak # | RetTime [min] | Type | Width [min] | Area [mAU*s] | Height [mAU] | Area % |
|---|---|---|---|---|---|---|
| 1 | 7.049 | VB | 0.1841 | 91.06892 | 7.47739 | 40.9751 |
| 2 | 8.754 | VB | 0.3107 | 131.18553 | 6.05758 | 59.0249 |
| Totals : | | | | 222.25446 | 13.53497 | |

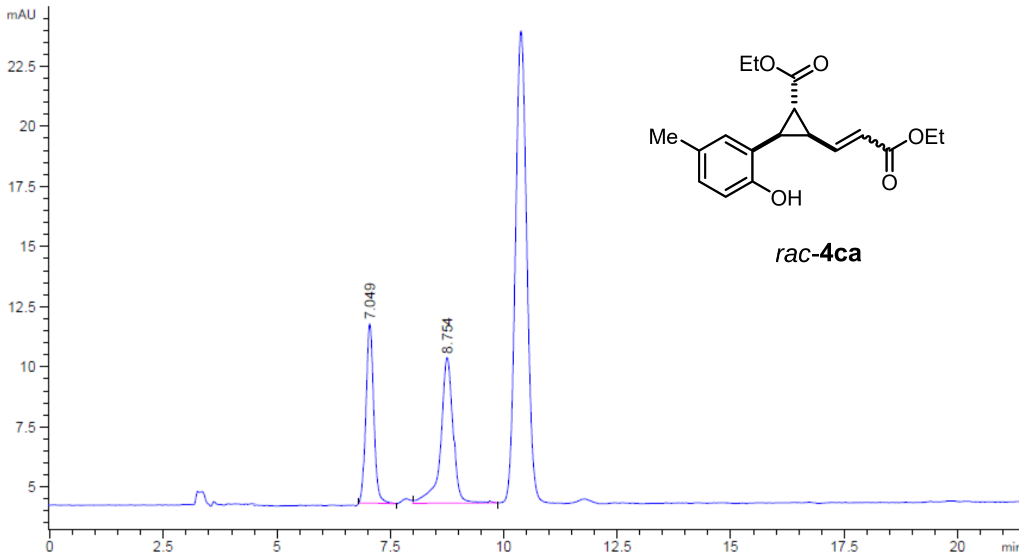


**Ethyl (1R,2S,3R)-2-(3-ethoxy-3-oxoprop-1-en-1-yl)-3-(2-hydroxy-5-methylphenyl)cyclopropane-1-carboxylate 4ca**

| Peak # | RetTime [min] | Type | Width [min] | Area [mAU*s] | Height [mAU] | Area % |
|---|---|---|---|---|---|---|
| 1 | 6.946 | BV | 0.1662 | 60.97028 | 5.63460 | 2.5248 |
| 2 | 7.196 | VB | 0.1635 | 23.43864 | 2.21344 | 0.9706 |
| 3 | 8.603 | BV | 0.2189 | 2330.41064 | 166.94904 | 96.5045 |
| Totals : | | | | 2414.81957 | 174.79707 | |

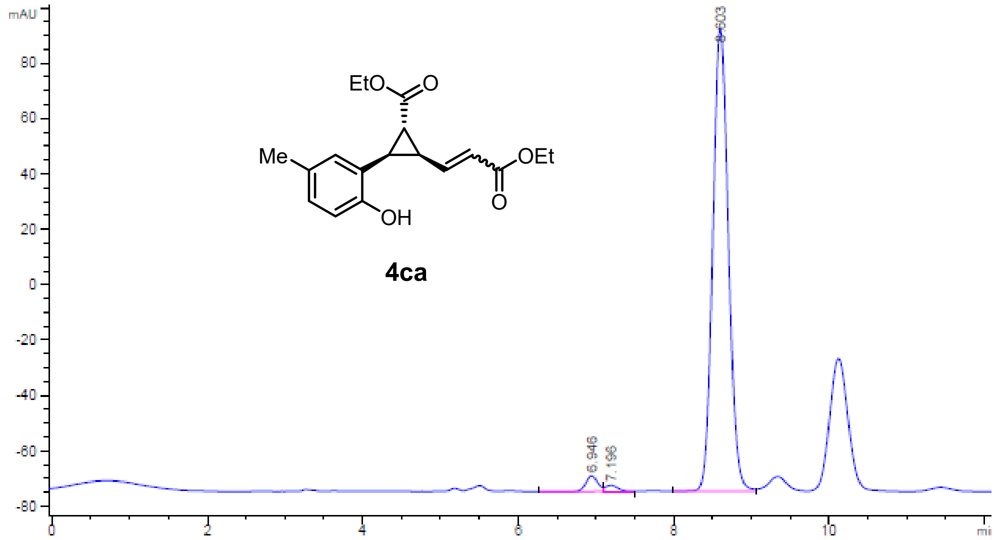

**Ethyl(1R*,2S*,3R)-2-(-3-ethoxy-3-oxoprop-1-en-1-yl)-3-(2-hydroxy-5-methoxyphenyl)cyclopropane -1-carboxylate 4da**

Signal 1: DAD1 A, Sig=254,4 Ref=360,100

| Peak # | RetTime [min] | Type | Width [min] | Area [mAU*s] | Height [mAU] | Area % |
|---|---|---|---|---|---|---|
| 1 | 10.141 | BB | 0.2583 | 319.48050 | 18.98366 | 56.7679 |
| 2 | 11.735 | BB | 0.2929 | 243.30342 | 12.96890 | 43.2321 |

Totals : 562.78392 31.95257

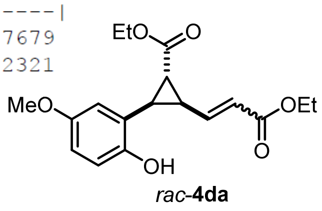


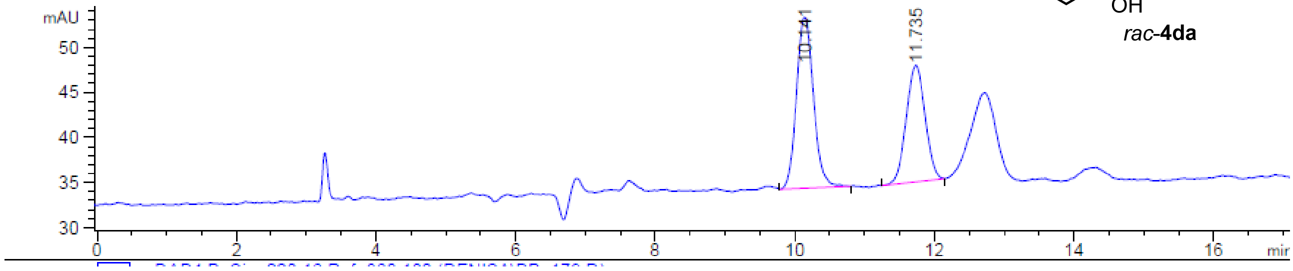


**Ethyl(1R,2S,3R)-2-(-3-ethoxy-3-oxoprop-1-en-1-yl)-3-(2-hydroxy-5-methoxyphenyl)cyclopropane -1-carboxylate 4ba**

| Peak # | RetTime [min] | Type | Width [min] | Area [mAU*s] | Height [mAU] | Area % |
|---|---|---|---|---|---|---|
| 1 | 10.164 | BB | 0.2538 | 22.87235 | 1.30941 | 1.6635 |
| 2 | 11.723 | BV | 0.3108 | 1170.94824 | 58.19378 | 98.3364 |

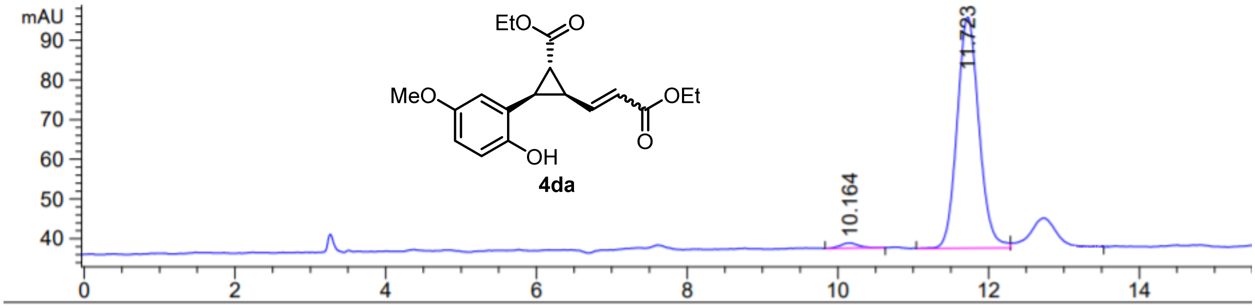

**Ethyl (1R*,2R*,3S*)-2-(5-chloro-2-hydroxyphenyl)-3-(-3-ethoxy-3-oxoprop-1-en-1-yl)cyclopropane-1-carboxylate 4ea**

| Peak # | RetTime [min] | Type | Width [min] | Area [mAU*s] | Height [mAU] | Area % |
|---|---|---|---|---|---|---|
| 1 | 5.089 | BV | 0.1245 | 969.53308 | 122.26378 | 58.7465 |
| 2 | 5.901 | VB | 0.1518 | 680.83551 | 71.04188 | 41.2535 |
| Totals : | | | | 1650.36859 | 193.30566 | |

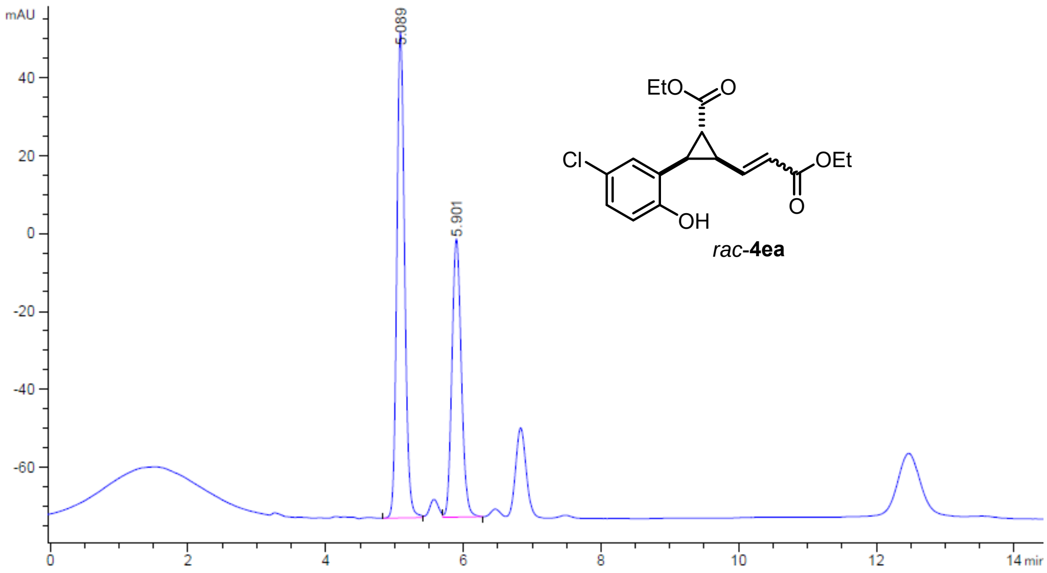


**Ethyl (1R,2R,3S)-2-(5-chloro-2-hydroxyphenyl)-3-(-3-ethoxy-3-oxoprop-1-en-1-yl)cyclopropane-1-carboxylate 4fa**

| Peak # | RetTime [min] | Type | Width [min] | Area [mAU*s] | Height [mAU] | Area % |
|---|---|---|---|---|---|---|
| 1 | 5.102 | BB | 0.1221 | 46.74147 | 5.91772 | 5.2954 |
| 2 | 5.907 | VV | 0.1508 | 835.93378 | 86.48706 | 94.7046 |
| Totals : | | | | 882.67525 | 92.40478 | |

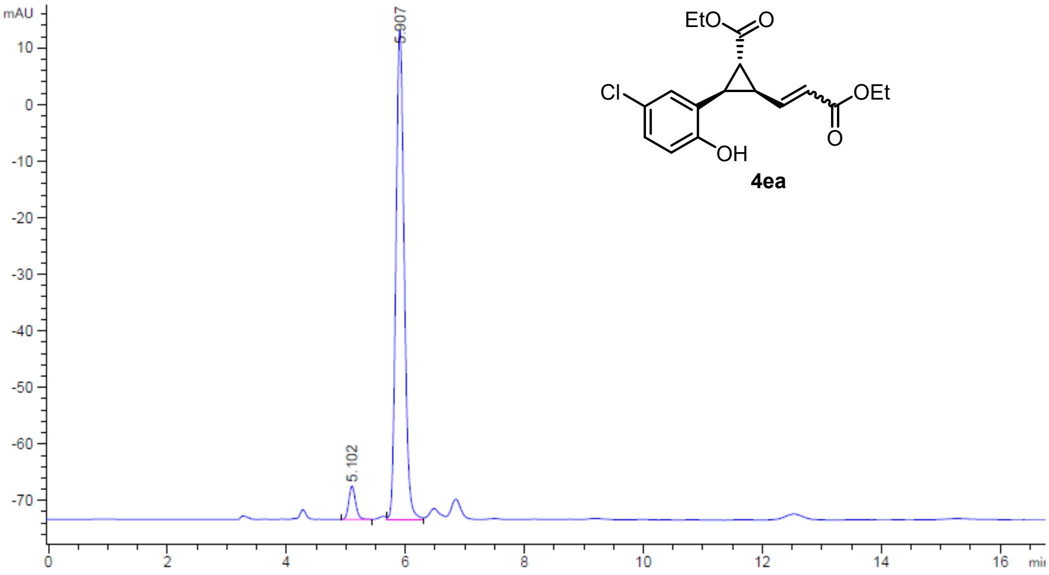

**Ethyl (1R*,2S*,3R*)-2-(3-ethoxy-3-oxoprop-1-en-1-yl)-3-(2-hydroxy-4-methoxyphenyl)cyclopropane-1-carboxylate 4fa**

Signal 1: DAD1 A, Sig=254,4 Ref=360,100

| Peak # | RetTime [min] | Type | Width [min] | Area [mAU*s] | Height [mAU] | Area % |
|---|---|---|---|---|---|---|
| 1 | 15.726 | BB | 0.8584 | 545.57031 | 9.72621 | 48.1374 |
| 2 | 20.915 | BB | 1.3468 | 587.78912 | 6.55085 | 51.8626 |
| Totals : | | | | 1133.35944 | 16.27706 | |

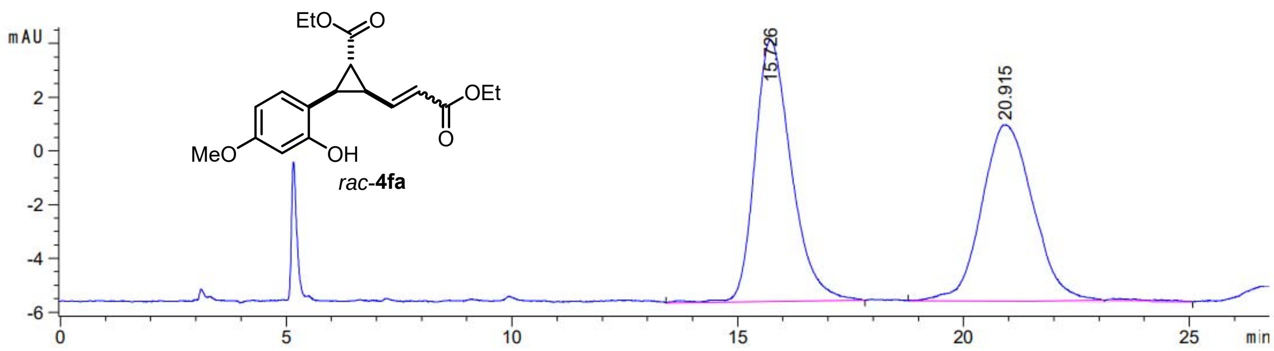


**Ethyl (1R,2S,3R)-2-(3-ethoxy-3-oxoprop-1-en-1-yl)-3-(2-hydroxy-4-methoxyphenyl)cyclopropane-1-carboxylate 4fa**

Signal 1: DAD1 A, Sig=254,4 Ref=360,100

| Peak # | RetTime [min] | Type | Width [min] | Area [mAU*s] | Height [mAU] | Area % |
|---|---|---|---|---|---|---|
| 1 | 16.713 | BB | 0.8369 | 66.84313 | 1.10624 | 7.3784 |
| 2 | 22.803 | VB | 1.3048 | 839.09265 | 9.66531 | 92.6216 |
| Totals : | | | | 905.93578 | 10.77155 | |

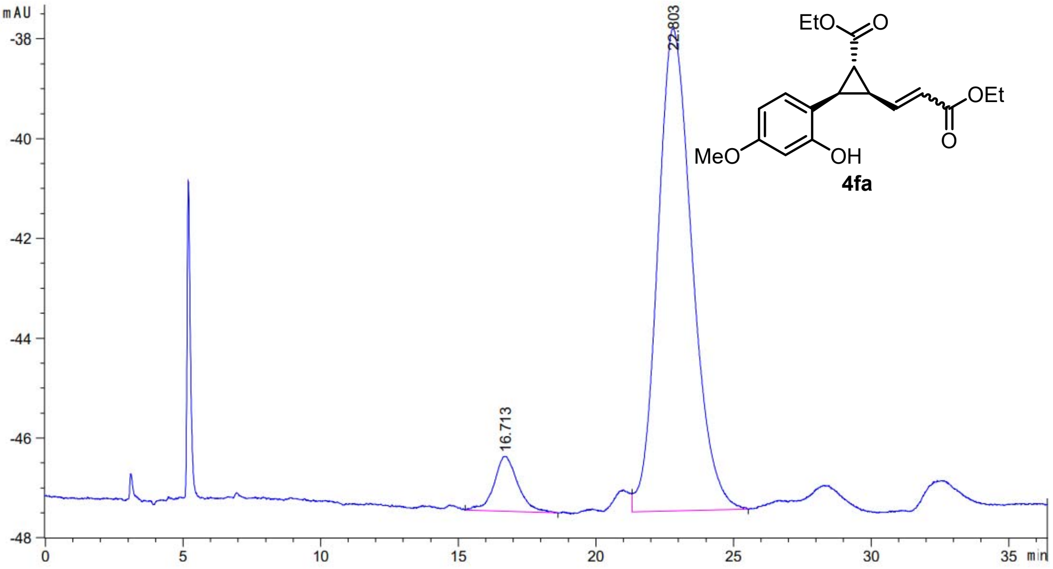

**Ethyl (1R*,2S*,3R*)-2-(3-ethoxy-3-oxoprop-1-en-1-yl)-3-(2-hydroxy-3-methoxyphenyl)cyclopropane-1-carboxylate 4ga**

| Peak # | RetTime [min] | Type | Width [min] | Area [mAU*s] | Height [mAU] | Area % |
|---|---|---|---|---|---|---|
| 1 | 12.596 | BV | 0.3021 | 1928.07642 | 98.59661 | 48.9097 |
| 2 | 15.303 | BV | 0.3678 | 2014.03845 | 85.19571 | 51.0903 |
| Totals : | | | | 3942.11487 | 183.79232 | |

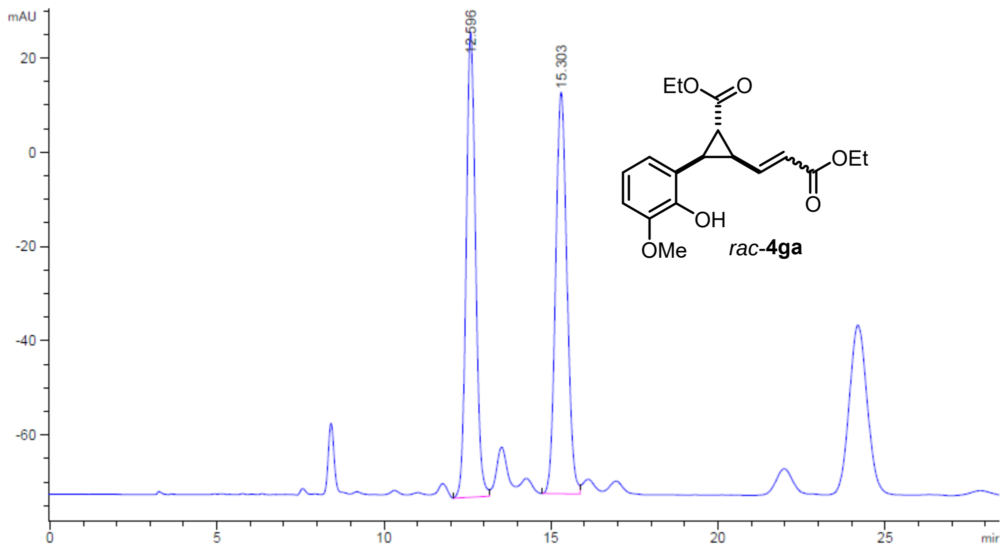


**Ethyl (1R,2S,3R)-2-(3-ethoxy-3-oxoprop-1-en-1-yl)-3-(2-hydroxy-3-methoxyphenyl)cyclopropane-1-carboxylate 4ea**

| Peak # | RetTime [min] | Type | Width [min] | Area [mAU*s] | Height [mAU] | Area % |
|---|---|---|---|---|---|---|
| 1 | 12.539 | BB | 0.2985 | 482.45602 | 25.07698 | 93.7354 |
| 2 | 15.248 | BV | 0.3654 | 32.24416 | 1.31818 | 6.2646 |
| Totals : | | | | 514.70019 | 26.39517 | |

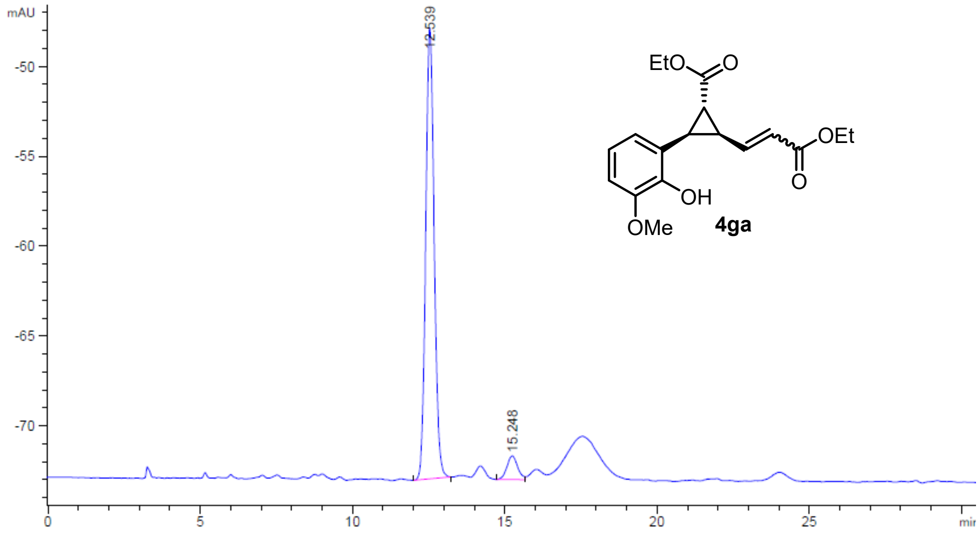

**Ethyl (1S*,1aS*,7bS*)-2-oxo-1,1a,2,7b-tetrahydrocyclopropa[*c*]chromene-1-carboxylate 5aa**

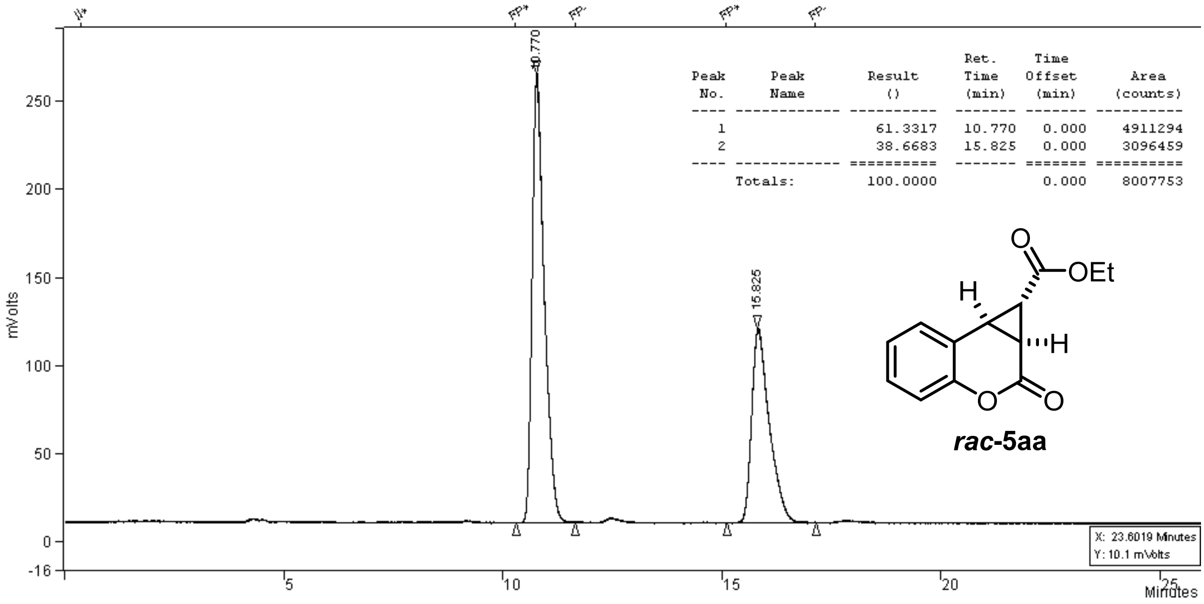


| Peak No. | Peak Name | Result () | Ret. Time (min) | Time Offset (min) | Area (counts) |
|---|---|---|---|---|---|
| 1 | | 61.3317 | 10.770 | 0.000 | 4911294 |
| 2 | | 38.6683 | 15.825 | 0.000 | 3096459 |
| | Totals: | 100.0000 | | 0.000 | 8007753 |

**Ethyl (1S*,1aS*,7bS*)-2-oxo-1,1a,2,7b-tetrahydrocyclopropa[*c*]chromene-1-carboxylate 5aa**

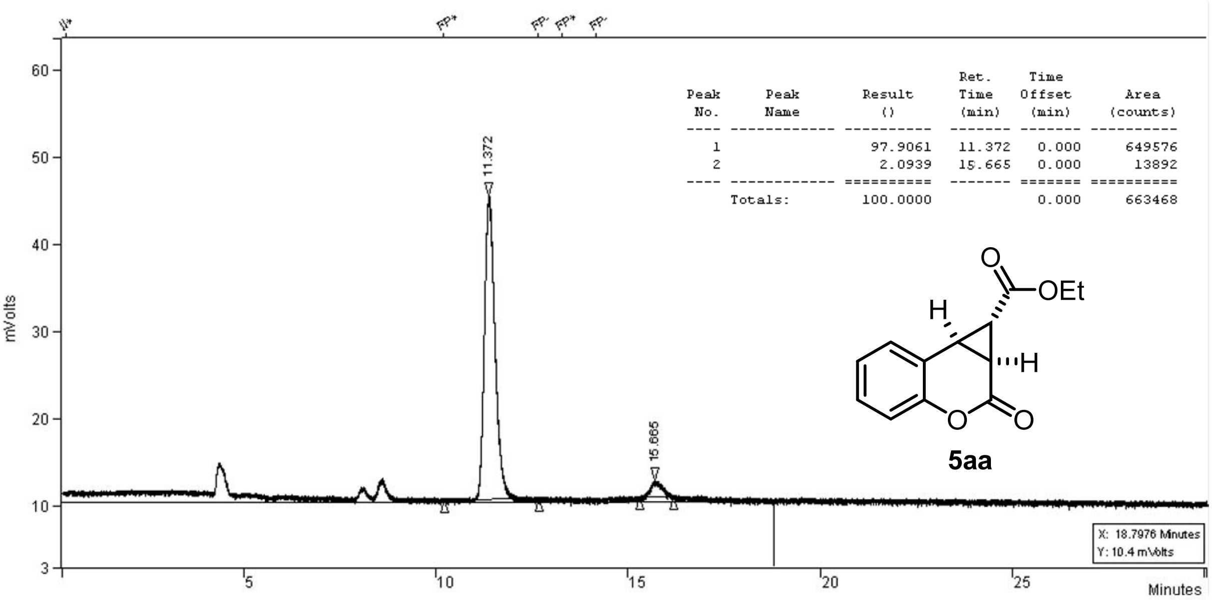


| Peak No. | Peak Name | Result () | Ret. Time (min) | Time Offset (min) | Area (counts) |
|---|---|---|---|---|---|
| 1 | | 97.9061 | 11.372 | 0.000 | 649576 |
| 2 | | 2.0939 | 15.665 | 0.000 | 13892 |
| | Totals: | 100.0000 | | 0.000 | 663468 |

**Ethyl (1S*,1aS*,2R*,7bR*)-2-(2-ethoxy-2-oxoethyl)-1,1a,2,7b-tetrahydrocyclopropa[*c*] chromene-1-carboxylate *cis*-8aa and Ethyl (1S*,1aS*,2S*,7bR*)-2-(2-ethoxy-2-oxoethyl)-1,1a,2,7b-tetrahydro cyclopropa[*c*]chromene-1-carboxylate *trans*-8aa (*trans*:*cis* ca. 9:1 favoring the *trans*-8aa isomer).**

| Peak # | RetTime [min] | Type | Width [min] | Area [mAU*s] | Height [mAU] | Area % |
|---|---|---|---|---|---|---|
| 1 | 16.981 | BB | 0.3233 | 237.06837 | 11.18567 | 4.6550 |
| 2 | 21.612 | BB | 0.4197 | 2269.65967 | 82.83414 | 44.5668 |
| 3 | 29.128 | BB | 0.5896 | 215.58414 | 5.74745 | 4.2332 |
| 4 | 31.966 | BB | 0.6643 | 2370.40747 | 54.40604 | 46.5450 |

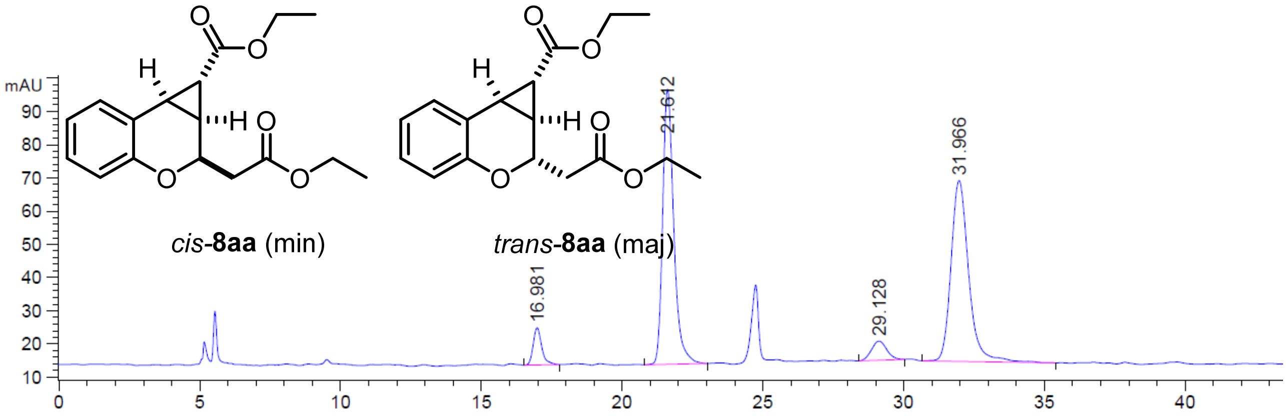


**Ethyl (1S,1aS,2R,7bR)-2-(2-ethoxy-2-oxoethyl)-1,1a,2,7b-tetrahydrocyclopropa[*c*] chromene-1-carboxylate *cis*-8aa**

Signal 1: DAD1 A, Sig=254,4 Ref=360,100

| Peak # | RetTime [min] | Type | Width [min] | Area [mAU*s] | Height [mAU] | Area % |
|---|---|---|---|---|---|---|
| 1 | 18.460 | BB | 0.2603 | 4.11539 | 2.32698e-1 | 0.3436 |
| 2 | 30.866 | BB | 0.6321 | 1193.65674 | 28.88521 | 99.6564 |

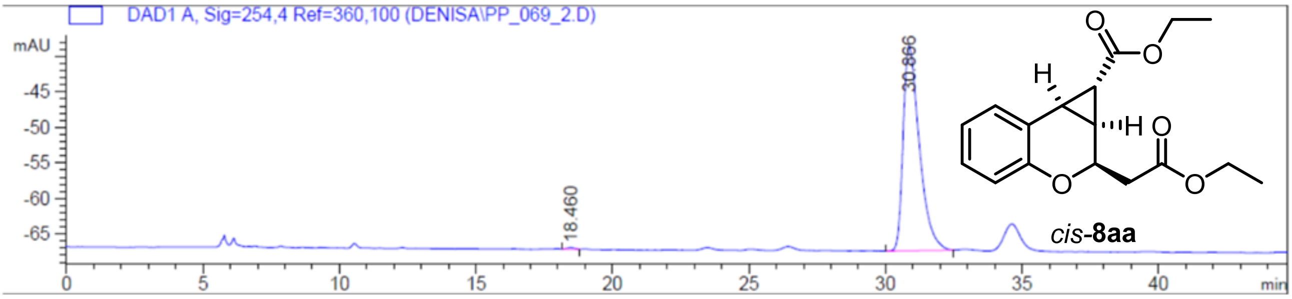

**Ethyl (1S,1aS,2S,7bR)-2-(2-ethoxy-2-oxoethyl)-1,1a,2,7b-tetrahydro cyclopropa[*c*]chromene-1-carboxylate *trans*-8aa**

Signal 1: DAD1 A, Sig=254,4 Ref=360,100

| Peak # | RetTime [min] | Type | Width [min] | Area [mAU*s] | Height [mAU] | Area % |
|---|---|---|---|---|---|---|
| 1 | 23.210 | BB | 0.4817 | 14.20832 | 4.53328e-1 | 0.6869 |
| 2 | 34.059 | BB | 0.6599 | 2054.15625 | 47.94344 | 99.3131 |

Totals : 2068.36457 48.39677

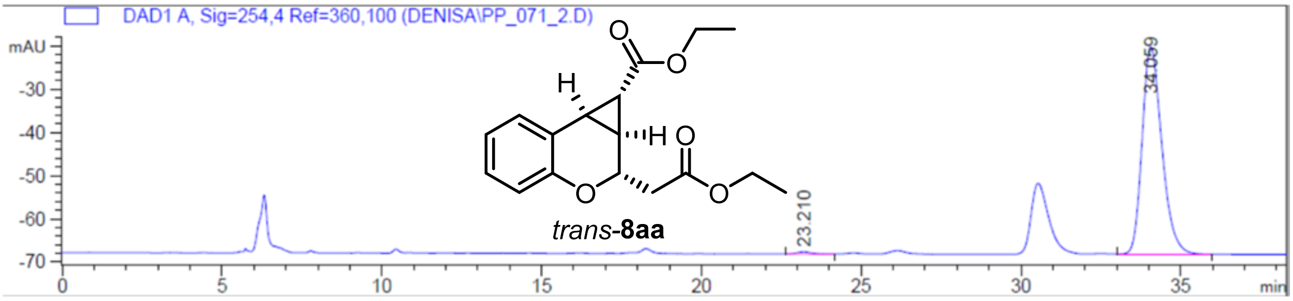

# Copies of IR spectra of products 4-8

**Ethyl (1R,2S,3R)-2-(3-ethoxy-3-oxoprop-1-en-1-yl)-3-(2-hydroxyphenyl)cyclopropane-1-carboxylate 4aa**

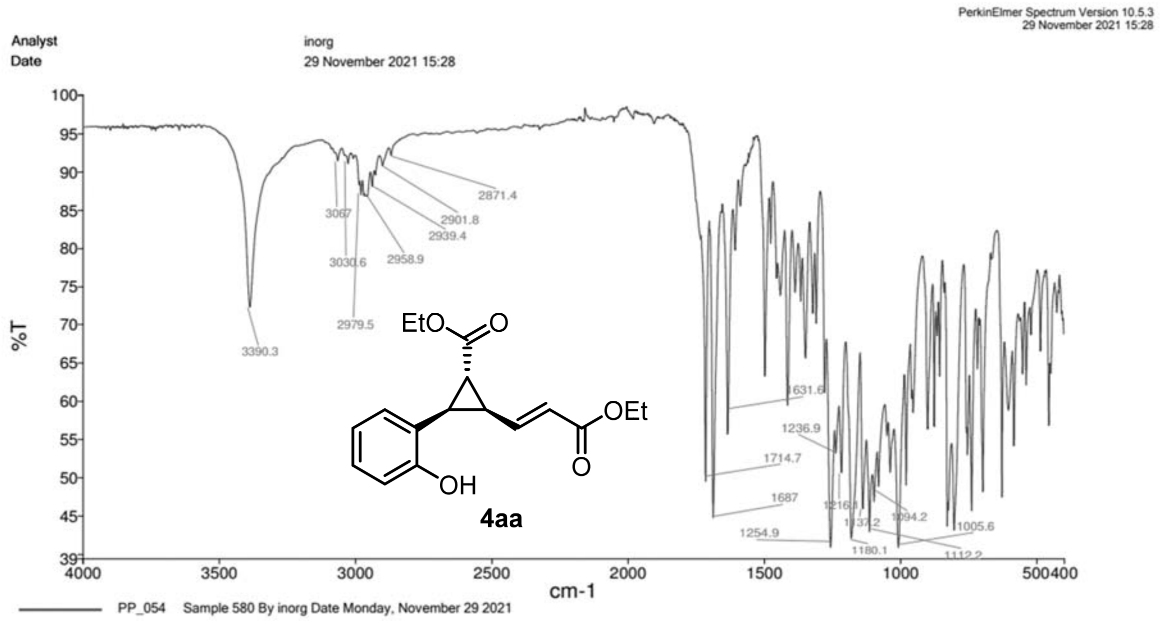


**Methyl (1R,2S,3R)-2-(3-ethoxy-3-oxoprop-1-en-1-yl)-3-(2-hydroxyphenyl)cyclopropane-1-carboxylate 4ab**

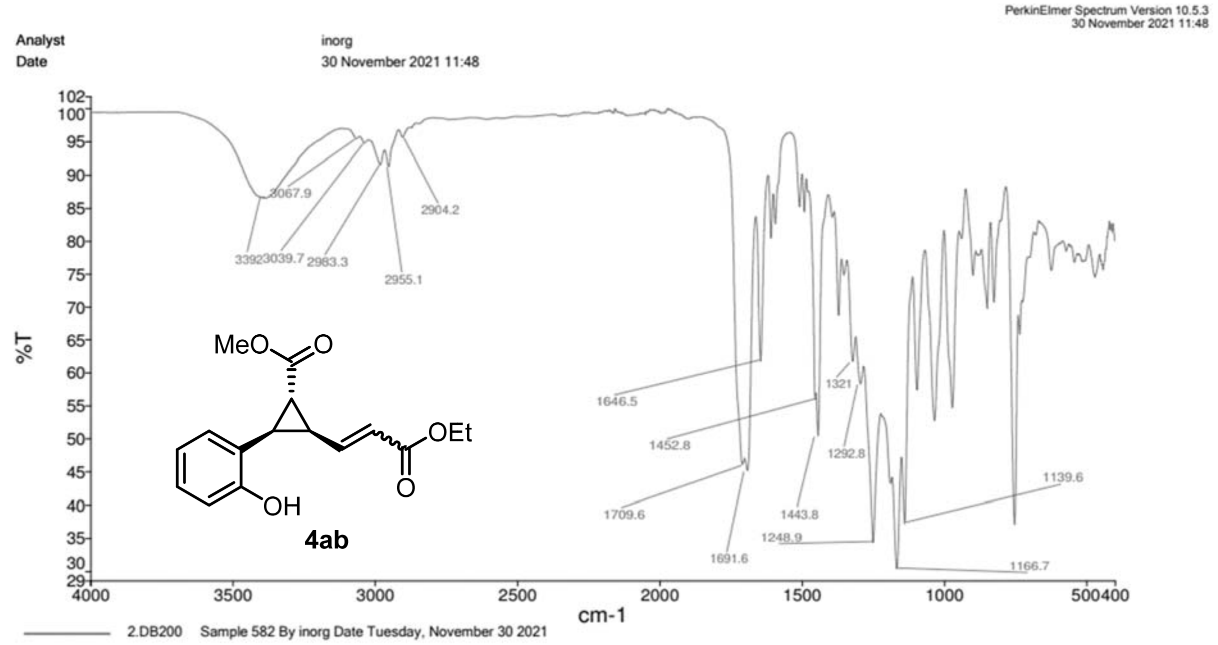

**Methyl (1R,2R,3S)-2-(2-hydroxyphenyl)-3-((E)-3-methoxy-3-oxoprop-1-en-1-yl)cyclopropane-1-carboxylate 4’ab**

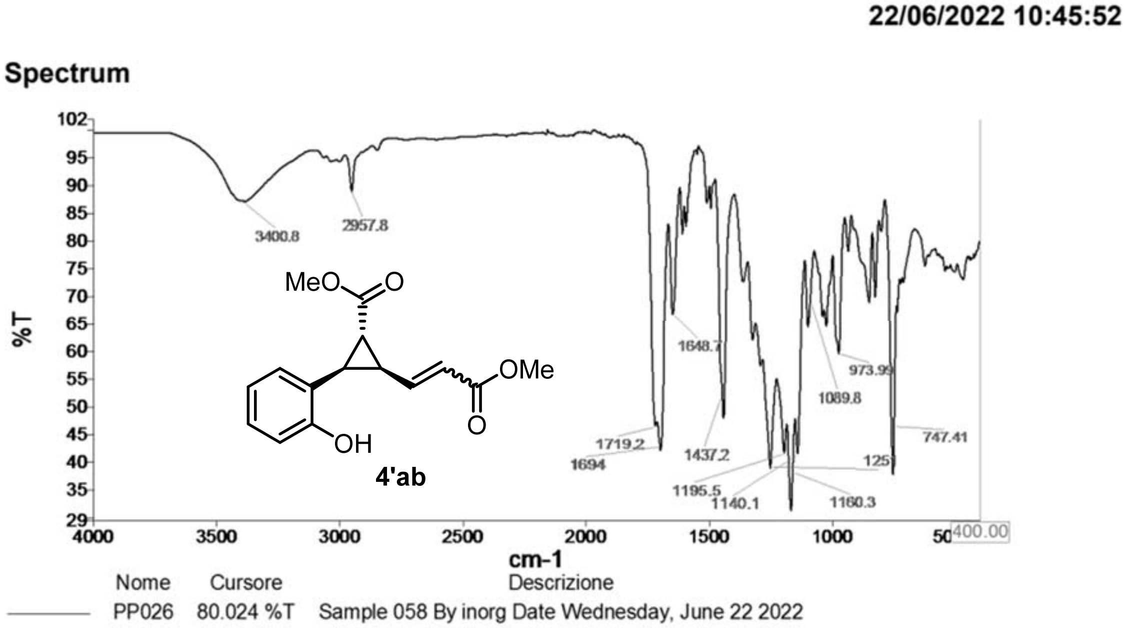


**Butyl (1R,2S,3R)-2-(3-ethoxy-3-oxoprop-1-en-1-yl)-3-(2-hydroxyphenyl)cyclopropane-1-carboxylatecarboxylate 4ac**

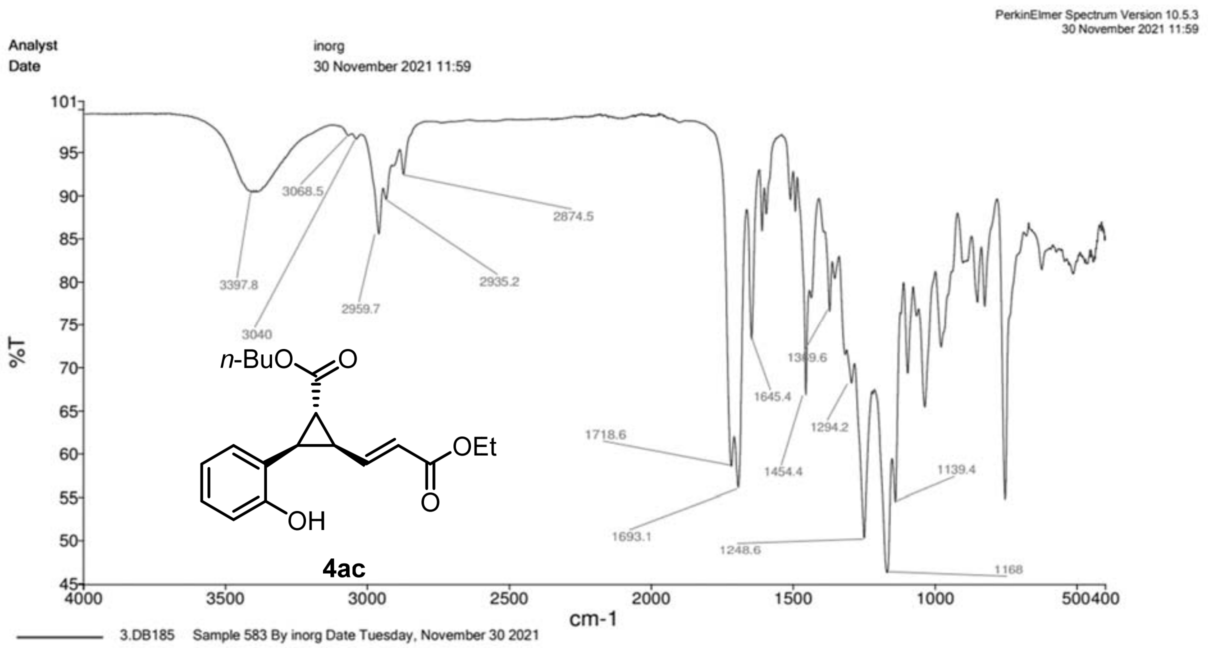

**Isobutyl (1R,2S,3R)-2-(-3-ethoxy-3-oxoprop-1-en-1-yl)-3-(2-hydroxyphenyl)cyclopropane-1-carboxylate 4ad**

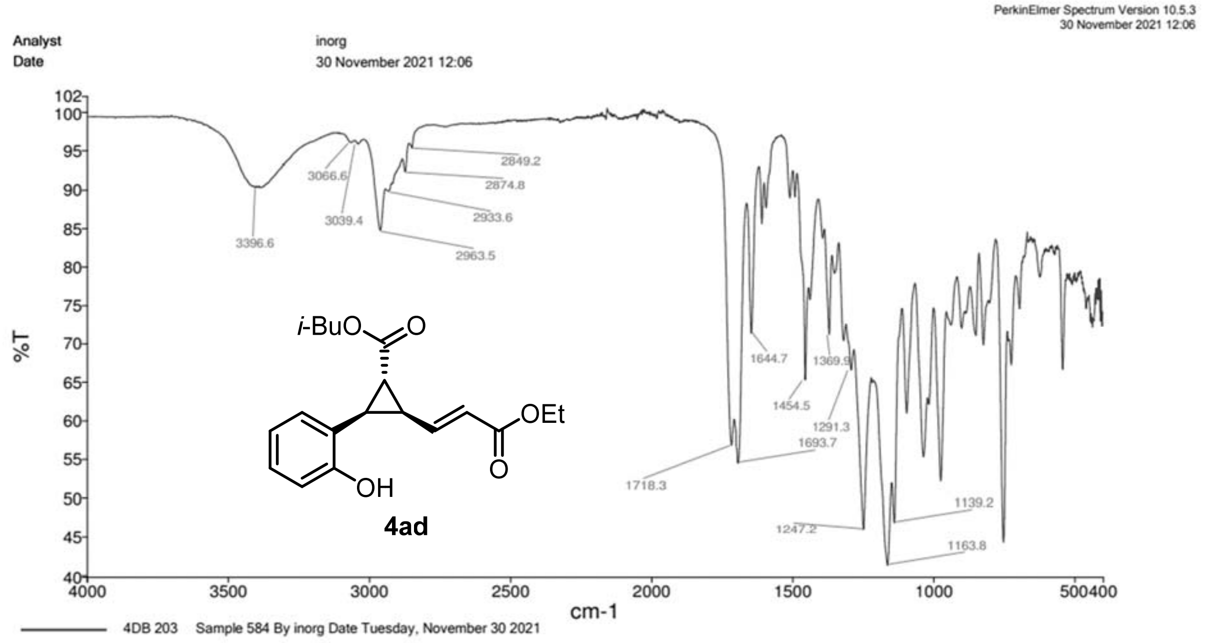


**Tert-butyl (1R,2S,3R)-2-(-3-ethoxy-3-oxoprop-1-en-1-yl)-3-(2-hydroxyphenyl)cyclopropane-1-carboxylate 4ae**

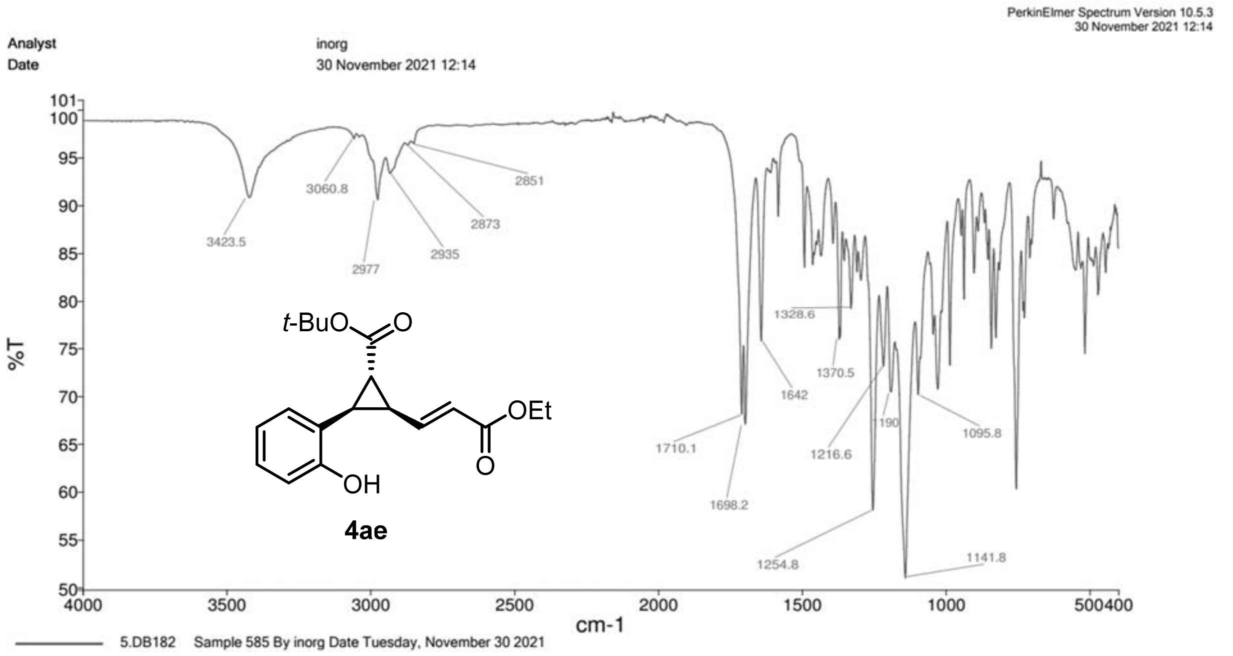

**Allyl (1R,2S,3R)-2-(3-ethoxy-3-oxoprop-1-en-1-yl)-3-(2-hydroxyphenyl)cyclopropane-1-carboxylate 4af**

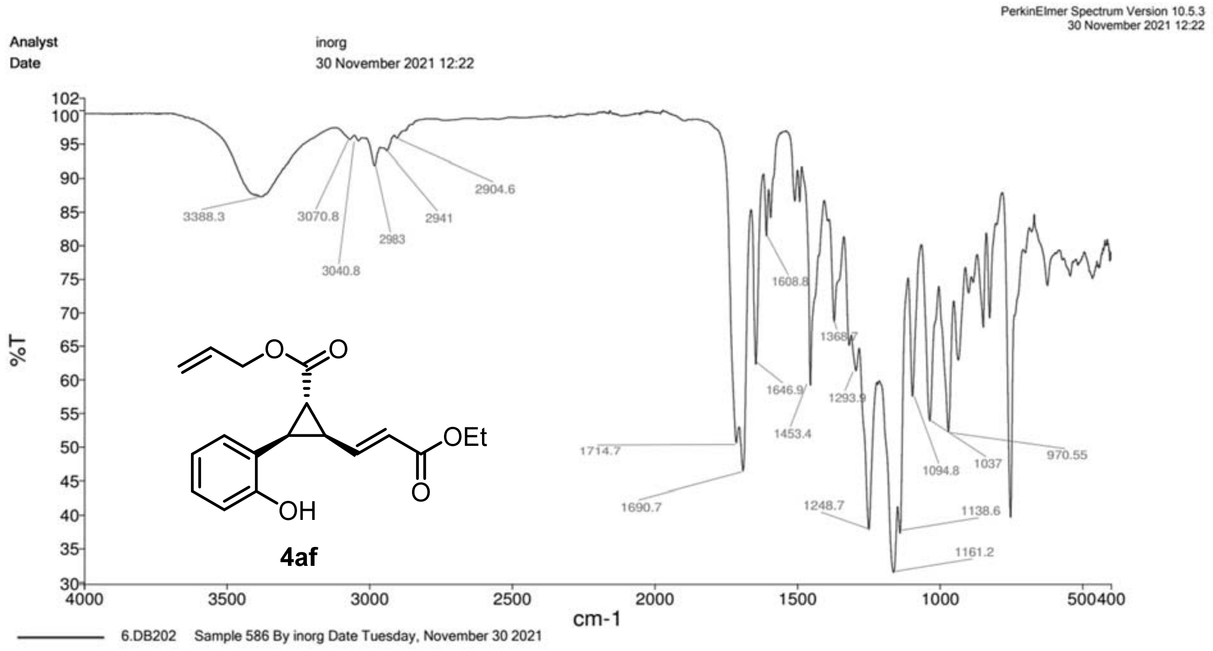


**Benzyl (1R,2S,3R)-2-(3-ethoxy-3-oxoprop-1-en-1-yl)-3-(2-hydroxyphenyl)cyclopropane-1-carboxylate 4ag**

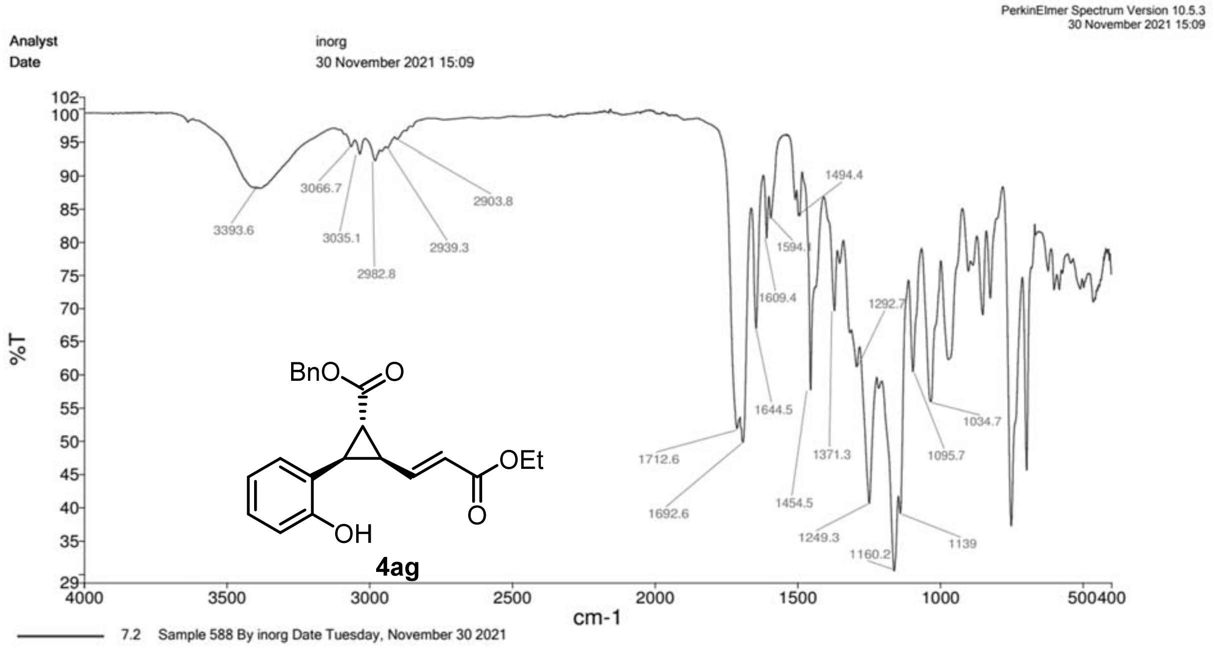

**Ethyl--3-((1S,2R,3R)-2-benzoyl-3-(2-hydroxyphenyl)cyclopropyl)acrylate 4ah**

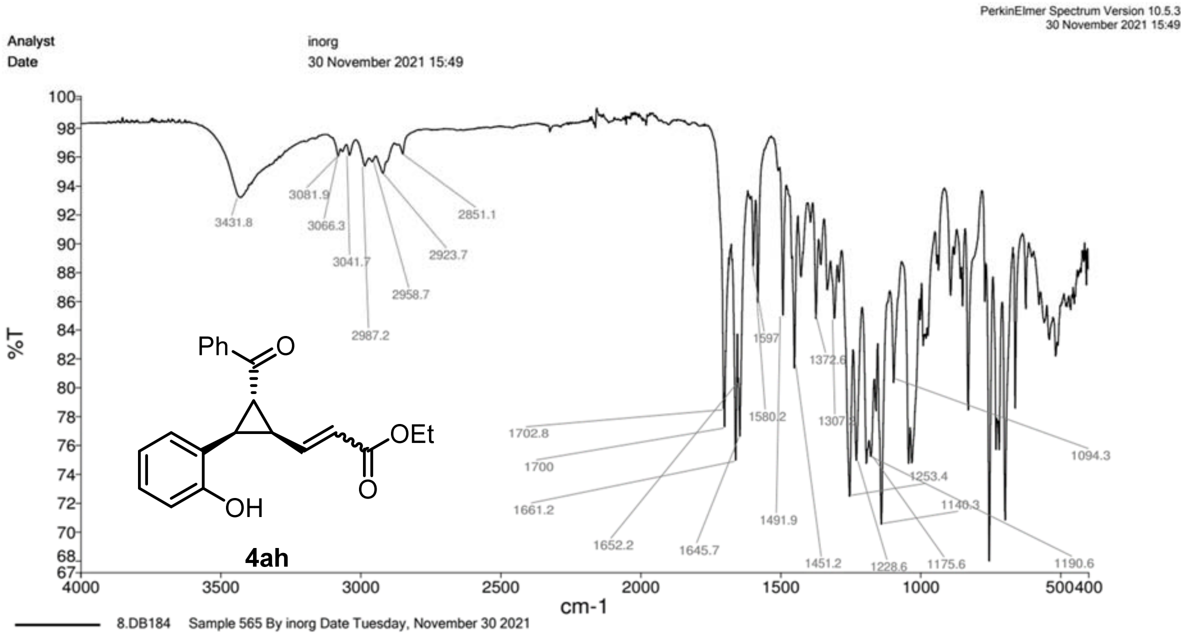


**Ethyl (1R,2S,3R)-2-(3-ethoxy-3-oxoprop-1-en-1-yl)-3-(2-hydroxy-4-methylphenyl)cyclopropane-1-carboxylate 4ba**

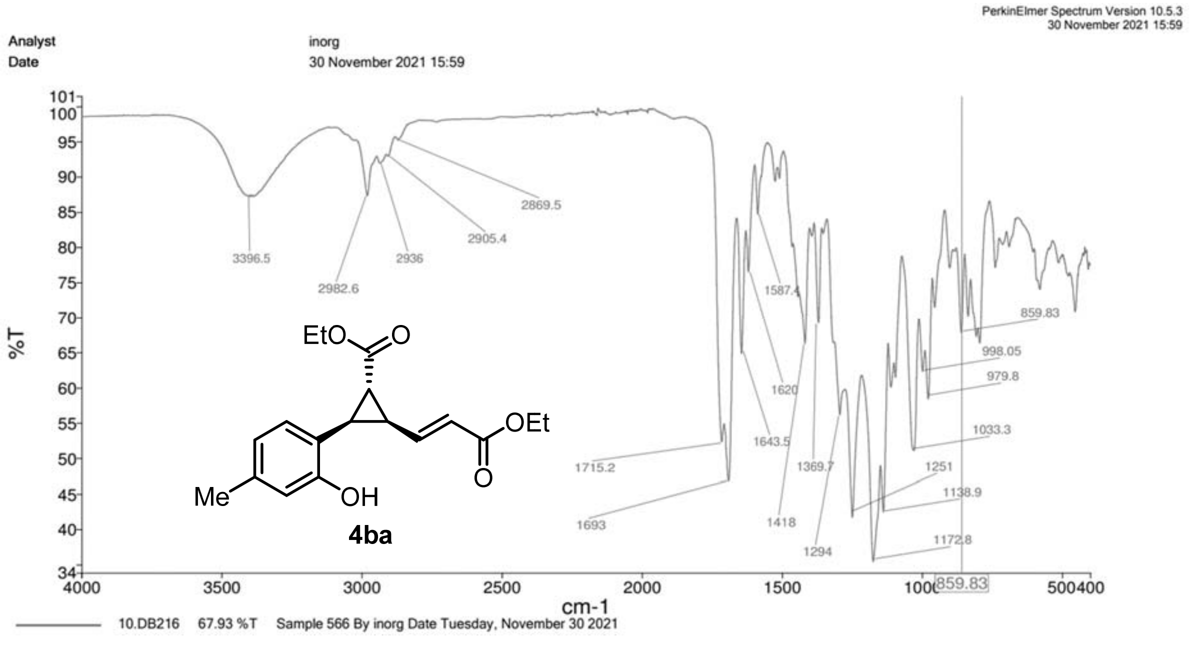

**Ethyl (1R,2S,3R)-2-(3-ethoxy-3-oxoprop-1-en-1-yl)-3-(2-hydroxy-5-methylphenyl)cyclopropane-1-carboxylate 4ca**

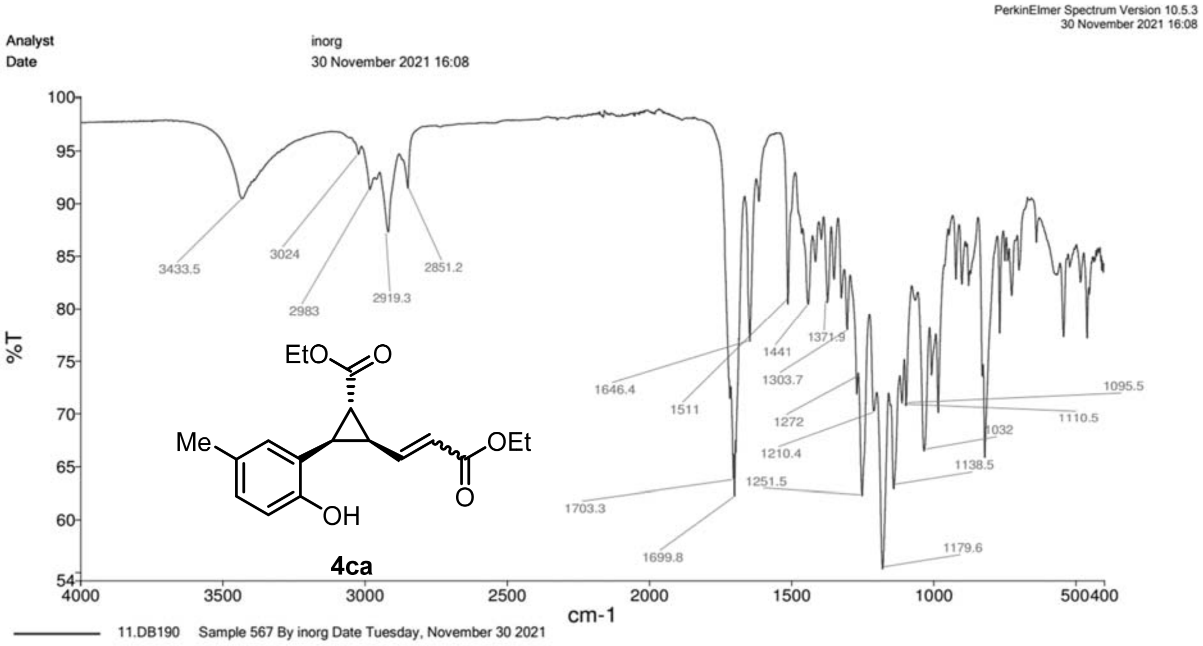


**Ethyl (1R,2S,3R)-2-(-3-ethoxy-3-oxoprop-1-en-1-yl)-3-(2-hydroxy-5-methoxyphenyl)cyclopropane -1-carboxylate 4da**

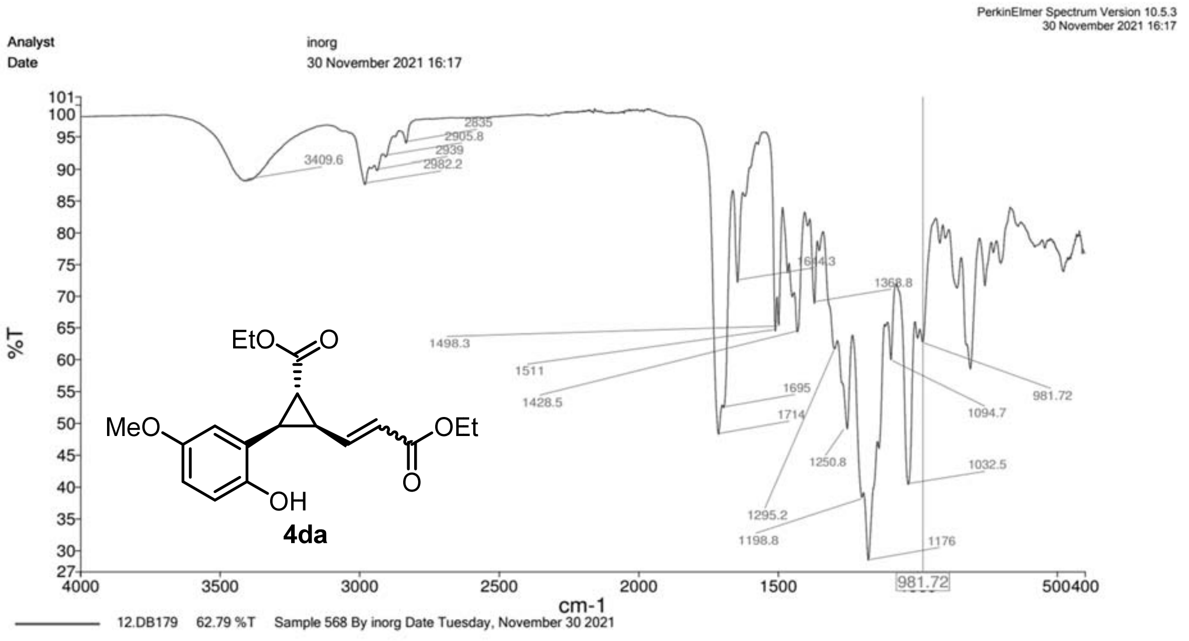

**Ethyl (1R,2R,3S)-2-(5-chloro-2-hydroxyphenyl)-3-(-3-ethoxy-3-oxoprop-1-en-1-yl)cyclopropane-1-carboxylate 4ea**

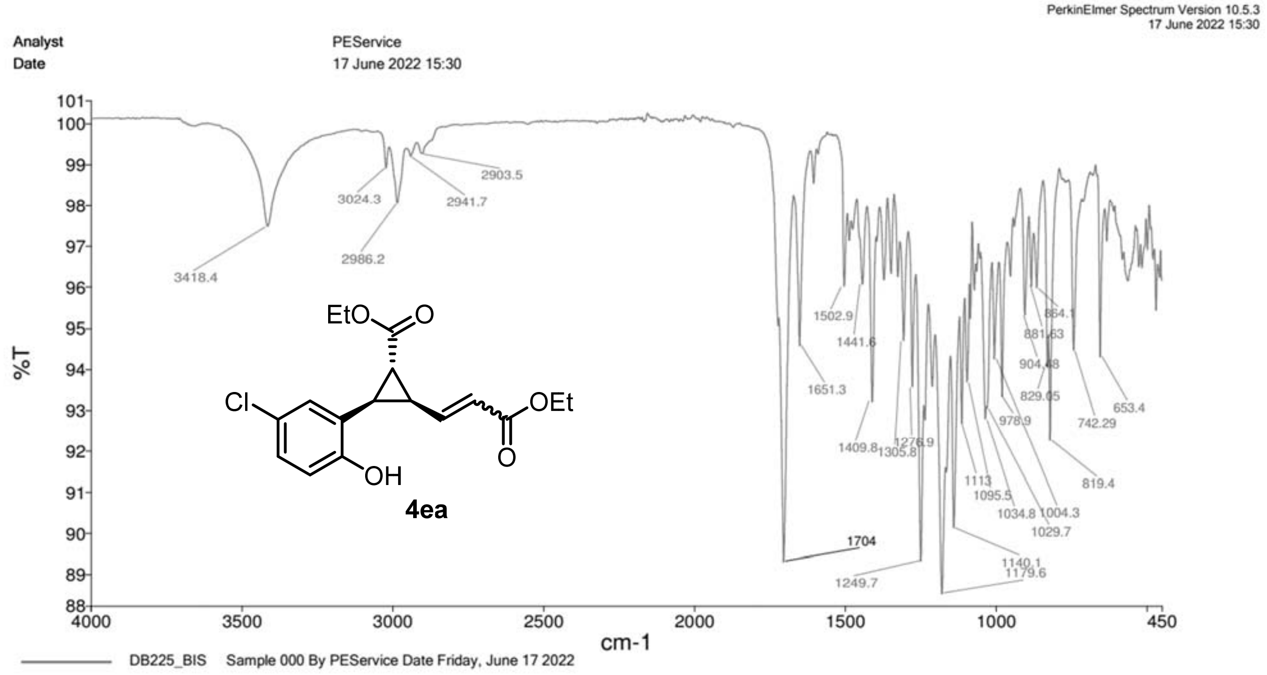


**ethyl (1R,2S,3R)-2-(3-ethoxy-3-oxoprop-1-en-1-yl)-3-(2-hydroxy-4-methoxyphenyl)cyclopropane-1-carboxylate 4fa**

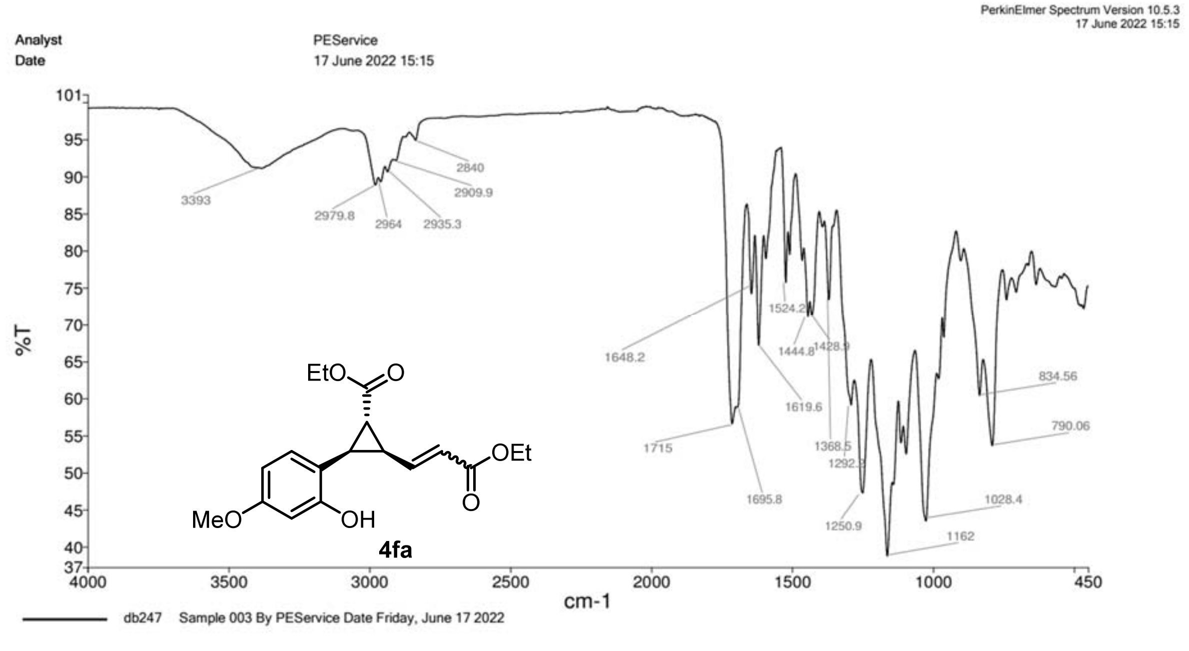

**Ethyl (1R,2S,3R)-2-(3-ethoxy-3-oxoprop-1-en-1-yl)-3-(2-hydroxy-3-methoxyphenyl)cyclopropane-1-carboxylate 4ga**

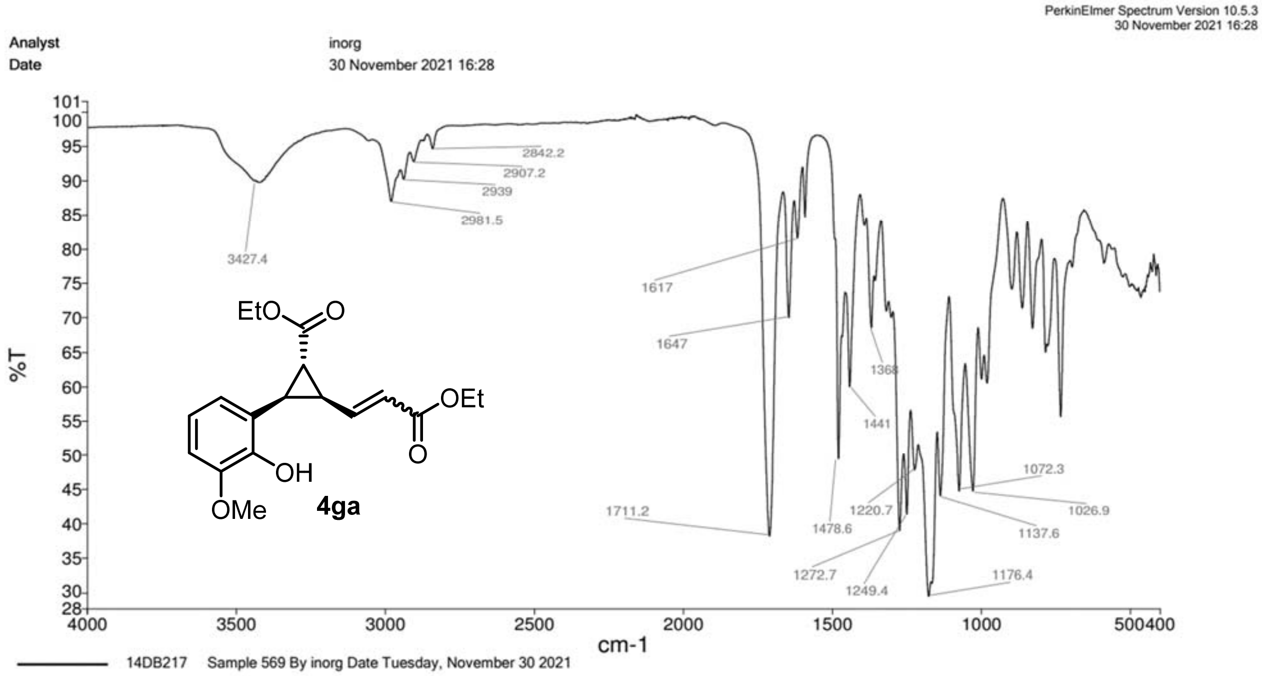


**Ethyl (1S,1aS,7bS)-2-oxo-1,1a,2,7b-tetrahydrocyclopropa[*c*]chromene-1-carboxylate 5aa**

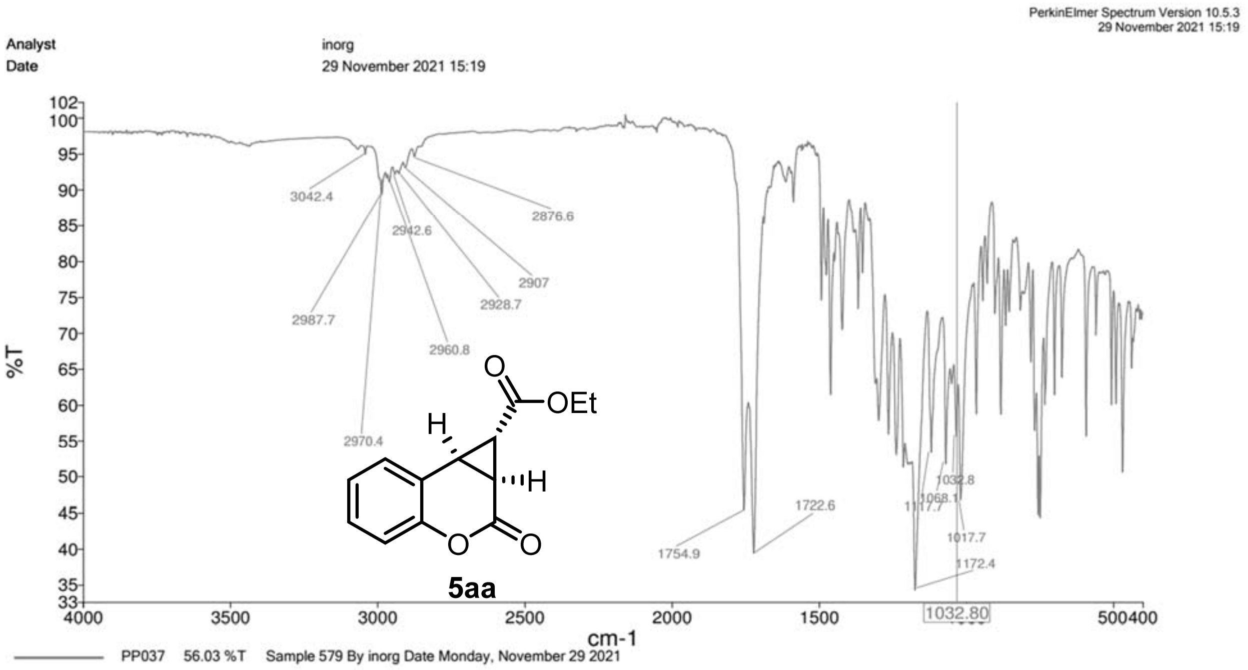

**Ethyl (1S,1aS,7bR)-1,1a,2,7b-tetrahydrocyclopropa[*c*]chromene-1-carboxylate 6aa**

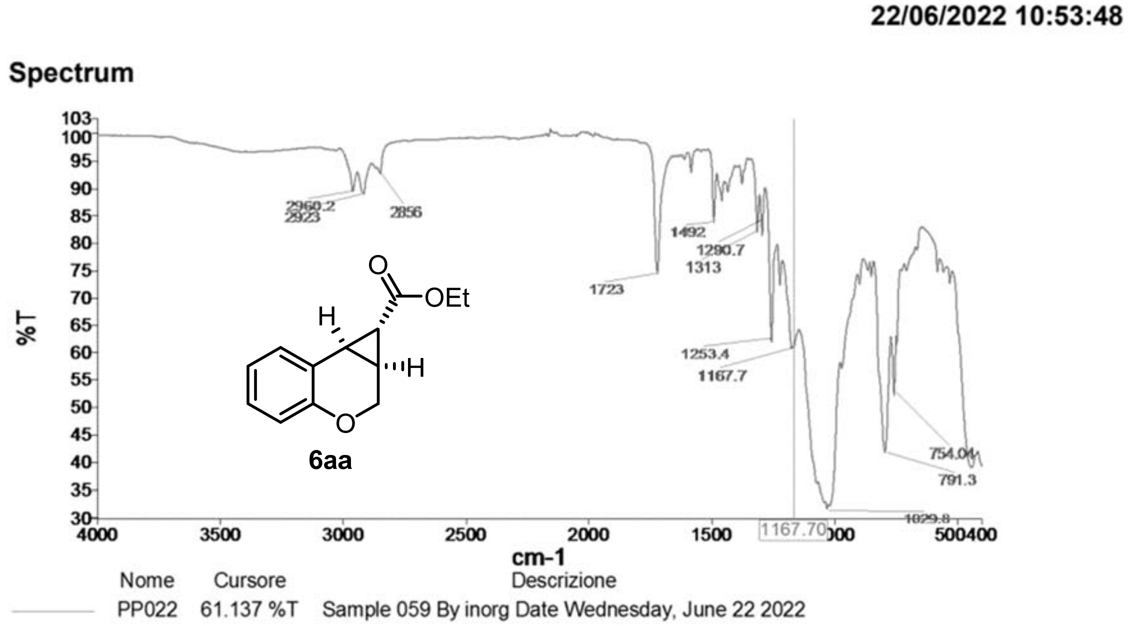


**Ethyl (1S,2S,3R)-2-(hydroxymethyl)-3-(2-hydroxyphenyl)cyclopropane-1-carboxylate 7aa**

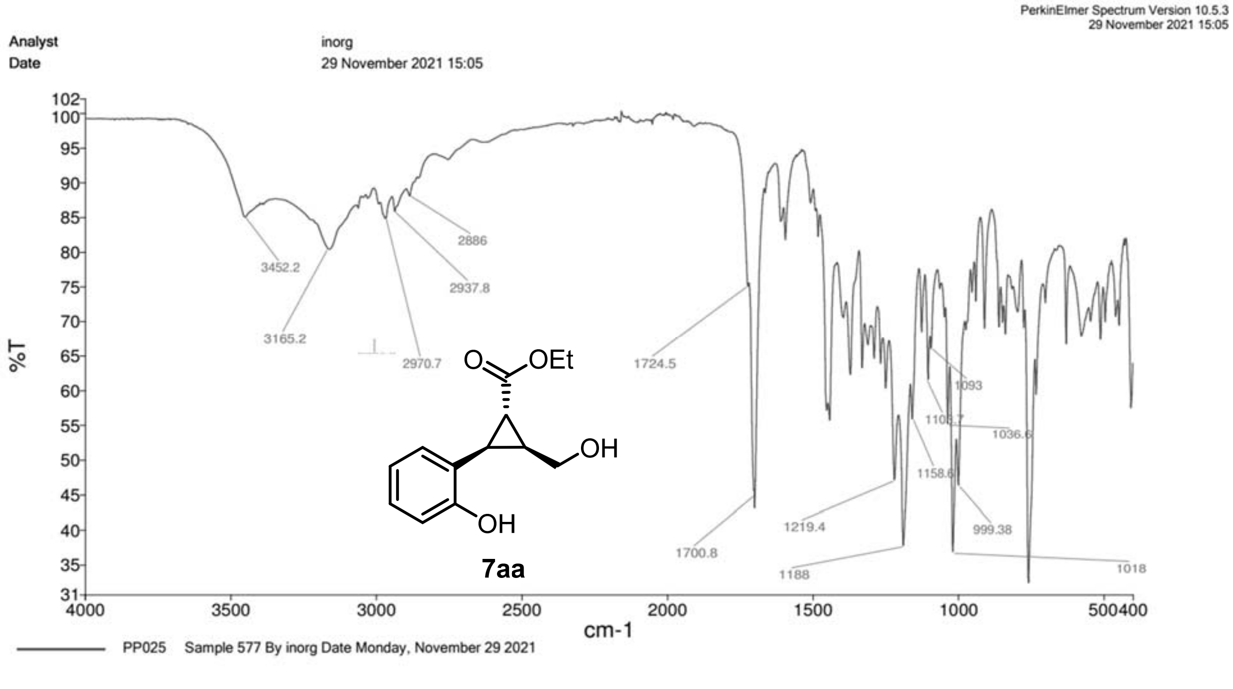

**Ethyl (1S,1aS,2R,7bR)-2-(2-ethoxy-2-oxoethyl)-1,1a,2,7b-tetrahydrocyclopropa[c]chromene-1-carboxylate *cis*-8aa**

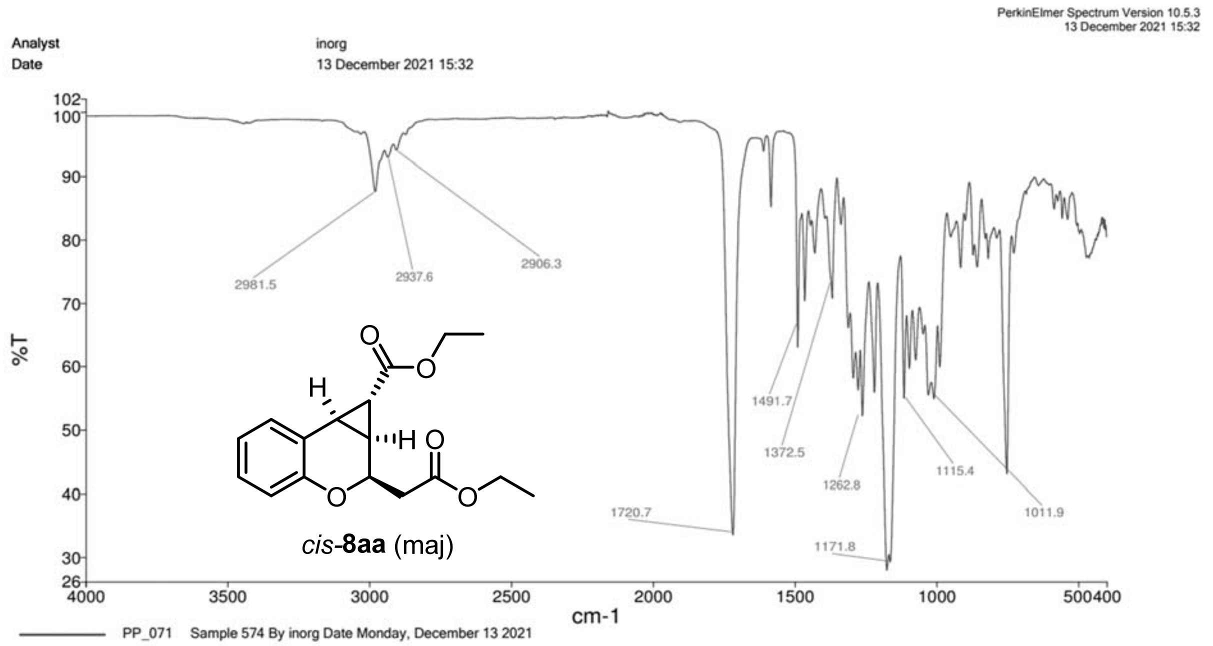


**Ethyl (1S,1aS,2S,7bR)-2-(2-ethoxy-2-oxoethyl)-1,1a,2,7b-tetrahydrocyclopropa[c]chromene-1-carboxylate *trans*-8aa**

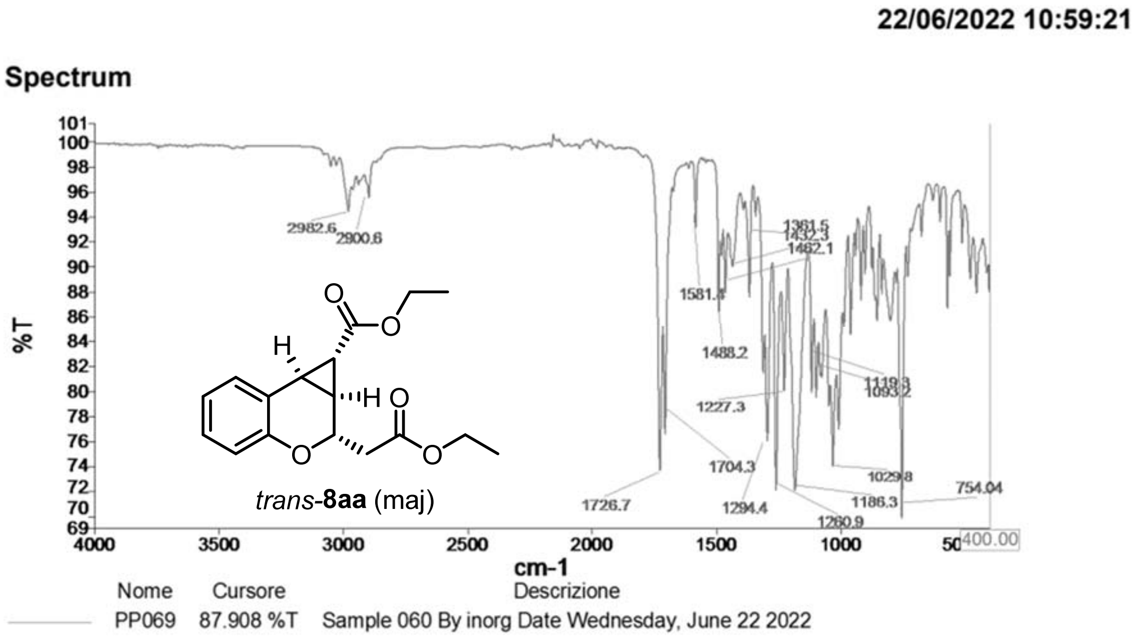

Supplement: Supplementary file 1 — ol2c02204_si_001.pdf [file ol2c02204_si_001.pdf]
